# Orchestrating Spatial Transcriptomics Analysis with Bioconductor

**DOI:** 10.1101/2025.11.20.688607

**Published:** 2025-11-21

**Authors:** Helena L. Crowell, Yixing Dong, Ilaria Billato, Peiying Cai, Martin Emons, Samuel Gunz, Boyi Guo, Mengbo Li, Alexandru Mahmoud, Artür Manukyan, Hervé Pagès, Pratibha Panwar, Shreya Rao, Callum J. Sargeant, Lori Shepherd Kern, Marcel Ramos, Jieran Sun, Michael Totty, Vincent J. Carey, Yunshun Chen, Leonardo Collado-Torres, Shila Ghazanfar, Kasper D. Hansen, Keri Martinowich, Kristen R. Maynard, Ellis Patrick, Dario Righelli, Davide Risso, Simone Tiberi, Levi Waldron, Raphael Gottardo, Mark D. Robinson, Stephanie C. Hicks, Lukas M. Weber

**Affiliations:** 1 National Center for Genomic Analysis, Barcelona, Spain.; 2 Biomedical Data Science Center, Lausanne University Hospital, Lausanne, Switzerland.; 3 University of Lausanne, Lausanne, Switzerland.; 4 Department of Biology, University of Padova, Padova, Italy.; 5 Department of Molecular Life Sciences, University of Zurich, Zurich, Switzerland.; 6 Swiss Institute of Bioinformatics, Zurich, Switzerland.; 7 Division of Biostatistics, Department of Population Health Sciences, University of Utah, Salt Lake City, UT, United States.; 8 Bioinformatics and Computational Biology Division, Walter and Eliza Hall Institute of Medical Research, Parkville, VIC, Australia.; 9 ACRF Cancer Biology and Stem Cells Division, Walter and Eliza Hall Institute of Medical Research, Parkville, VIC, Australia.; 10 Department of Medical Biology, The University of Melbourne, Parkville, VIC, Australia.; 11 Channing Division of Network Medicine, Mass General Brigham, Boston, MA, United States.; 12 Berlin Institute for Medical Systems Biology, Max-Delbrück-Center for Molecular Medicine in the Helmholtz Association, Berlin, Germany.; 13 Fred Hutch Cancer Center, Seattle, WA, United States.; 14 School of Mathematics and Statistics, The University of Sydney, Camperdown, NSW, Australia.; 15 Sydney Precision Data Science Centre, The University of Sydney, Camperdown, NSW, Australia.; 16 Charles Perkins Centre, The University of Sydney, Camperdown, NSW, Australia.; 17 Centre for Cancer Research, The Westmead Institute for Medical Research, The University of Sydney, Camperdown, NSW, Australia.; 18 Roswell Park Comprehensive Cancer Center, Buffalo, NY, United States.; 19 Institute for Implementation Science in Population Health, City University of New York Graduate School of Public Health and Health Policy, New York, NY, United States.; 20 Department of Epidemiology and Biostatistics, City University of New York Graduate School of Public Health and Health Policy, New York, NY, United States.; 21 Department of Biostatistics, Johns Hopkins Bloomberg School of Public Health, Baltimore, MD, United States.; 22 Lieber Institute for Brain Development, Johns Hopkins Medical Campus, Baltimore, MD, United States.; 23 Center for Computational Biology, Johns Hopkins University, Baltimore, MD, United States.; 24 Department of Genetic Medicine, Johns Hopkins School of Medicine, Baltimore, United States.; 25 Department of Biomedical Engineering, Johns Hopkins University, Baltimore, MD, United States.; 26 Department of Psychiatry and Behavioral Sciences, Johns Hopkins School of Medicine, Baltimore, MD, United States.; 27 Solomon H. Snyder Department of Neuroscience, Johns Hopkins School of Medicine, Baltimore, MD, United States.; 28 Johns Hopkins Kavli Neuroscience Discovery Institute, Baltimore, MD, United States.; 29 Department of Electrical Engineer and Information Technology, University of Naples “Federico II”, Naples, Italy.; 30 Department of Statistical Sciences, University of Padova, Padova, Italy.; 31 Padua Center for Network Medicine, University of Padova, Padova, Italy.; 32 Department of Statistical Sciences, University of Bologna, Bologna, Italy.; 33 School of Life Sciences, Ecole Polytechnique Fédérale de Lausanne, Lausanne, Switzerland.; 34 Center for Computational Biology, Johns Hopkins University, Baltimore, MD, United States.; 35 Malone Center for Engineering in Healthcare, Johns Hopkins University, Baltimore, MD, United States.; 36 Department of Biostatistics, Boston University School of Public Health, Boston, MA, United States.

**Keywords:** spatial transcriptomics, spatially-resolved transcriptomics, spatial omics, gene expression, high-dimensional data, computational biology, data analysis, workflow, open-source, reproducible research, interoperability, R, Bioconductor

## Abstract

Spatial transcriptomics technologies provide spatially-resolved measurements of gene expression through assays that can either target selected genes or capture transcriptome-wide expression profiles. The complexity and variability of these technologies and their associated data necessitate multi-step workflows integrating diverse computational methods and software packages. We provide a freely accessible, open-source, continuously updated and tested online book containing reproducible code examples, datasets, and discussion about data analysis workflows for spatial omics data using Bioconductor in R, including interoperability with Python.

Spatial transcriptomics technologies are widely used in biomedical research including cancer biology, neuro-science, immunology, and developmental biology [1, 2, 3, 4, 5]. These technologies enable the quantification of spatially-resolved gene expression within tissue sections, providing powerful information on tissue organization and interactions between cells. A number of protocols and technologies are available, which differ in their spatial resolution, the number of genes that can be detected, and sensitivity and specificity. Spatial transcriptomics technologies may be grouped into sequencing-based, which capture RNA from an untargeted or transcriptome-scale set of transcripts at barcoded spatial locations using a sequencing-based readout, and imaging-based, which use a fluorescent readout to identify individual RNA molecules from a typically targeted set of transcripts at subcellular spatial resolution and can be aggregated to cellular resolution [2, 3, 4, 5]. Technologies for other modalities, including spatial proteomics [6, 7, 8] and spatial multi-omics [9], provide further views of spatially-resolved molecular and histological features within cells and tissues.

Computational analyses of spatial transcriptomics data consist of a complex sequence of analysis steps, including preprocessing, quality control, intermediate processing, and downstream analyses, which are connected into workflows. Numerous methods are available for each analysis step (e.g. see [4] for a review). A crucial task for data analysts is to select appropriate computational methods for each step given the data type and experimental context, and to connect the inputs and outputs of different methods in a modular manner to build a complete workflow. Most methods are implemented as R or Python software packages. Standardized data structures, such as *SpatialExperiment* [10] (R/Bioconductor), *AnnData* [11] and *SpatialData* [12] (Python), and structures in the *Seurat* [13] and *Giotto Suite* [14] frameworks (R), facilitate connections between methods. Extensions provide additional capacity for data types from specific technologies (e.g. [15, 16]), or to convert data structures between R and Python (e.g. [17, 18]). Preprocessing steps are generally platform-specific, depending on the format of the raw data (e.g. read alignment or cell segmentation). After preprocessing, the data are usually summarized as a gene expression count table, aggregated at the level of spatial locations (e.g. spots, beads, or bins) or single cells. Subsequent analysis steps use the gene expression count table together with the spatial information as the starting point, e.g. for quality control, feature selection, dimensionality reduction, clustering, and differential testing. Many of these analysis steps are adapted from single-cell RNA sequencing analysis workflows (e.g. [19]), with adaptations to the properties of spatial data such as taking into account distances between observations and considering the number of cells per spatial location. Various downstream analyses, e.g. spatially-aware cell type compositional and interaction analyses, are also applicable to spatial proteomics and other spatial omics data [7, 8, 9].

Bioconductor is a long-standing community-based pro ject that aims to develop and share open-source R-based software for high-throughput biological data analysis [20, 21, 22]. The Bioconductor project started in 2001, and has grown to include more than 2,300 software packages (as of the October 2025 release; Figure S1). Software packages are contributed by numerous research groups around the world, while the overall project and core infrastructure are coordinated and maintained by the Bioconductor Core Team, advised by Community, Technical, and Scientific Advisory Boards. Bioconductor components are primarily developed as R packages, with extensions facilitating interoperability with Python. Since software packages and infrastructure are developed by various research groups, these can include the latest state-of-the-art methods and tools, thus providing a rich, flexible, and modular analysis framework for end users. Bioconductor packages undergo continuous build testing, which notifies package maintainers of any installation or runtime errors. Notably, users and developers benefit from documentation requirements and code review, community-based forums, and educational resources [23]. Bioconductor-based workflows can also incorporate R packages from the Comprehensive R Archive Network (CRAN), providing access to an extensive history of R packages implementing advanced statistical methods in areas including (generalized) linear modeling and spatial statistics, machine learning tools, and sophisticated graphical visualization tools.

Here, we provide a freely accessible, open-source resource consisting of an online book containing reproducible code examples, datasets, and discussion about analyses of spatial transcriptomics data using Bioconductor. Chapters cover individual analysis steps as well as extended workflows, using downloadable datasets from several commercially available technologies. The book is hosted on the Bioconductor website, and the code examples are regularly tested on the Bioconductor build system on several operating systems, ensuring reliability, stability, and long-term accessibility for users. The code examples use R packages from either Bioconductor or CRAN, and some chapters further demonstrate interoperability with Python packages from PyPI using *reticulate* [24] and/or *basilisk* [25]. Datasets used in the examples are stored remotely and can be downloaded using functions provided in a companion Bioconductor package *OSTA.data* [26]. Figure 1 provides a schematic overview of the book content (additional details in Online methods), and Figure 2 illustrates how the resource fits within the Bioconductor and wider R and Python analysis ecosystems. Table S1 summarizes technologies, datasets, and methods covered in the book.

Existing frameworks and tutorials for data analysis workflows for spatial transcriptomics include *Seurat* [13], *Giotto Suite* [14], *Museum of spatial transcriptomics* [4], and *Voyager* [15] (in R), and *Squidpy* [27] (in Python) (Figure S2). A key advantage of our approach is that both the overall resource and the included methods and tools are developed by various research groups from multiple institutions and countries, thus ensuring that we have included a wide range of representative state-of-the-art scientific methods and analysis approaches. We also emphasize R-Python interoperability with examples in several chapters. In addition, the modularity of the Bioconductor ecosystem allows users to easily adapt our workflows to include new methods, and the continuous Bioconductor build testing ensures that examples remain error-free, while the Bioconductor support site and community forums provide accessible venues for users to ask questions. The development version of the book is hosted on GitHub, which enables additional continuous testing using a GitHub Actions workflow, and provides an additional interface for users to submit issues, provide suggestions and feedback, and contribute content.

Other existing resources provide code examples, tutorials, and discussion on guidelines for analyses of single-cell RNA sequencing data, with extensions to spatially-resolved data, including *Orchestrating Single-Cell Analysis with Bioconductor* (OSCA) [19] in R using Bioconductor, and *Single-Cell Best Practices* [28] in Python using the *scverse* project [29]. By contrast, our book focuses on spatially-resolved omics data, beginning with introductory discussion on data types and using example datasets from several technologies. This allows us to focus in more detail on the methodological issues and available methods for spatially-resolved data. For some analyses, single-cell methods can be repurposed to provide a computationally efficient baseline for method comparisons, which we discuss in the relevant sections. One limitation is that we restrict code examples to methods available as R packages from Bioconductor or CRAN, or Python packages from PyPI. We also discuss several key methods available from other sources (e.g. packages from GitHub or other non-package code repositories), but do not include these within the reproducible code examples. This restriction is intended to facilitate long-term accessibility and maintenance. We also do not provide a complete listing of all available methods for each analysis step, instead focusing on widely used methods and those that we have found to be well documented, accessible, and high-performing. For readers interested in exploring the literature in more detail, we include references to benchmark evaluation papers and reviews comparing additional available methods; selected related resources are also listed in the book appendix. Our resource is intended as a community-driven, living document that will be updated and extended to cover new methods, data types, and technologies as they become available. The book is available from Bioconductor at https://bioconductor.org/books/OSTA/, and we welcome suggestions, feedback, and contributions from the spatial omics research community.

## Online methods

### OSTA book infrastructure, hosting, and testing

The *Orchestrating Spatial Transcriptomics Analysis with Bioconductor* (OSTA) book is built using open-source publishing tools including *Quarto*, *R Markdown*, *bookdown*, and the *BiocBook* [30] Bioconductor package. The rendered version of the book is hosted on the Bioconductor website at https://bioconductor.org/books/OSTA/, and the source code is publicly accessible from Bioconductor and GitHub. The initial version of the book was released as part of Bioconductor version 3.22. The book infrastructure and overall approach are based on and extend previous related Bioconductor resources including *Orchestrating Single-Cell Analysis with Bioconductor* (OSCA) [19]. Consistent with standard Bioconductor package development guidelines, we maintain separate *release* and development (*devel*) versions, where the release version is relatively stable and intended for use by most readers, and the development version incorporates latest updates and extensions. The release version is updated to match the development version approximately every six months. Both versions are regularly tested on several operating systems using the Bioconductor Build System infrastructure (up to three times per week), ensuring that all code examples run error-free, and dependency packages are accessible. In addition, we maintain a GitHub Actions workflow in the GitHub repository to run tests when updates are made to the source code. The GitHub page also facilitates interaction with the wider community, allowing users to submit issues to provide suggestions and feedback, as well as code contributions.

### OSTA book structure and content

The OSTA book is structured as a series of parts and chapters containing reproducible code examples and text discussion on analyses for spatial omics data. The chapters include introductory and background chapters, “analysis” chapters that each cover a specific analysis step, and “workflow” chapters that contain extended workflows for datasets from several major technological platforms. The chapters are organized into parts relating to certain types of technologies (e.g. sequencing-based and imaging-based platforms) or types of analyses (e.g. platform-independent, multiple-sample, and cross-platform analyses). The reproducible code examples make use of downloadable datasets stored in an online repository, which can be accessed programmatically using functions provided in the companion Bioconductor package *OSTA.data* [26].

**Figure 1:**
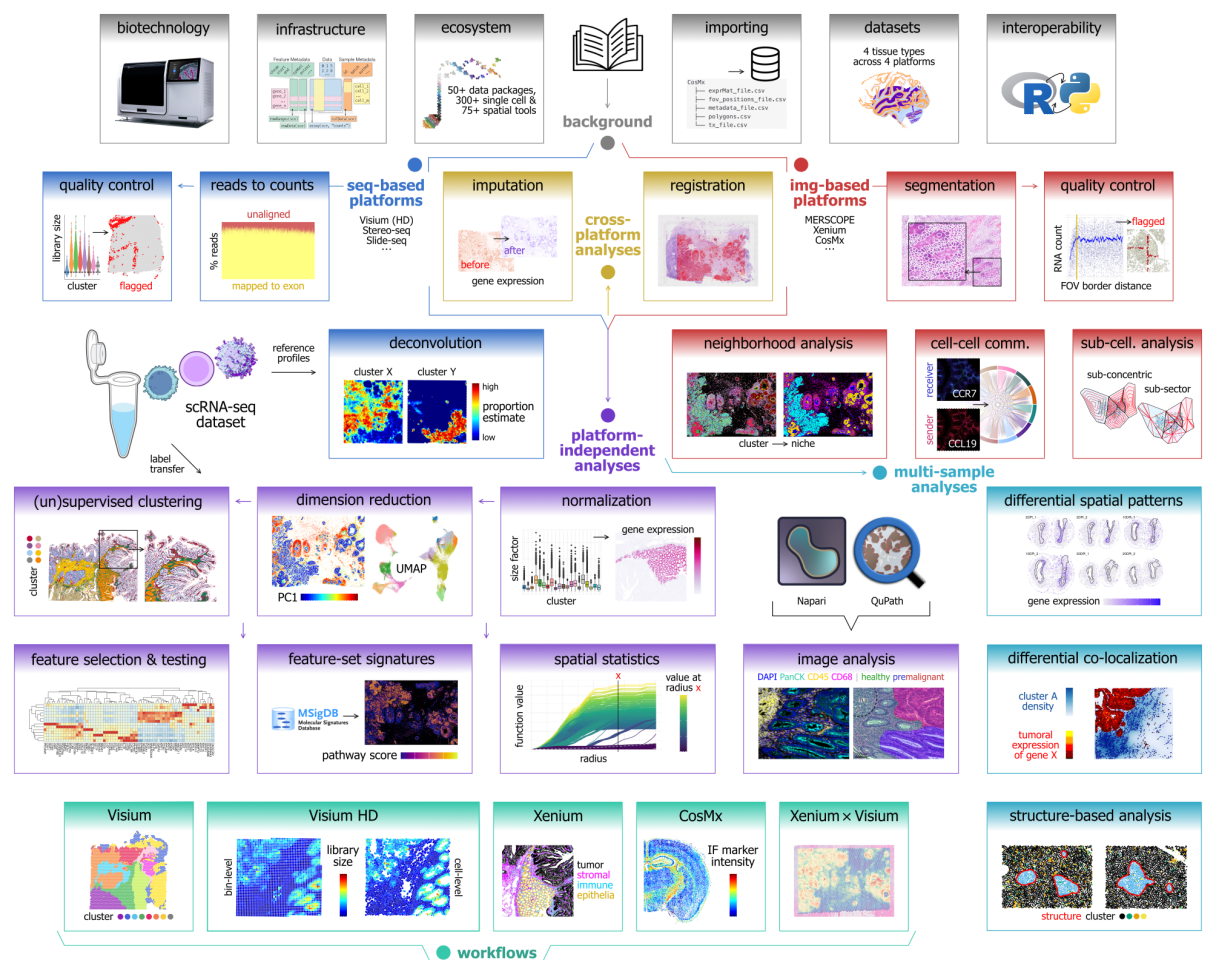
Schematic overview of Orchestrating Spatial Transcriptomics Analysis with Bioconductor (OSTA) book content. The OSTA book consists of a series of chapters grouped into parts, including introduction and background (gray), analyses applicable to sequencing-based technologies (dark blue) and imaging-based technologies (red), platform-independent analyses (purple), multiple-sample analyses (light blue), and cross-platform analyses (yellow). Individual chapters cover individual analysis steps as well as extended workflows for datasets from several major technologies (cyan). Reference single-cell RNA sequencing data may be used for deconvolution of sequencing-based data, and (semi-)supervised clustering of any data; image features may be extracted from, for instance, immunofluorescence or hematoxylin and eosin (H&E) stains using Napari and QuPath, respectively. Arrows indicate an approximate order for a computational data analysis workflow, however, numerous alternative methods are available at each step and may require different processing of data. In summary, OSTA offers the building blocks needed to construct modular data analysis workflows that require careful selection of methods by analysts, depending on the data type, experimental design, and biological question.

This section provides an overview of the parts and chapters in the current version of the OSTA book (as of November 2025). The chapters are organized into the following parts, listed below. The complete content, including recent updates, can be viewed in the online version of the book. For a schematic overview, see Figure 1. Table S1 provides a summary of the technologies, datasets, and methods covered in the book. Workflow chapters for a representative selection of technologies (Visium, Visium HD, Xenium, and CosMx) are additionally provided as Supplementary Documents.

- *Background*: introductory material on spatial omics, file formats, data representations, infrastructure and analysis frameworks (in R/Bioconductor and Python, commercial solutions), and interoperability with Python, including the following chapters: *Introduction*, *Spatial omics*, *Infrastructure*, *Ecosystem*, *Importing data*, *Example datasets*, and *Python interoperability*.
- *Sequencing-based platforms*: analyses and workflows for data from sequencing-based platforms, including the following chapters: *Introduction*, *Reads to counts*, *Quality control*, *Intermediate processing*, *Deconvolution*, *Workflow: Visium DLPFC*, *Workflow: Visium CRC*, and *Workflow: Visium HD*.
- *Imaging-based platforms*: analyses and workflows for data from imaging-based platforms, including the following chapters: *Introduction*, *Segmentation*, *Quality control*, *Intermediate processing*, *Neighborhood analysis*, *Cell-cell communication*, *Sub-cellular analysis*, *Workflow: Xenium*, and *Workflow: CosMx*.
- *Platform-independent analyses*: downstream analyses and workflows that are applicable to data from both types of platforms, including the following chapters: *Normalization*, *Dimensionality reduction*, *Clustering*, *Feature selection & testing*, *Feature-set signatures*, *Spatial statistics*, and *Image analysis*.
- *Multiple-sample analyses*: analyses and workflows applicable to datasets consisting of multiple samples (e.g. multiple tissue sections), including the following chapters: *Differential spatial patterns*, *Differential colocalization*, and *Structure-based analysis*.
- *Cross-platform analyses*: downstream analyses and workflows to integrate or combine information across platforms, including the following chapters: *Spatial registration*, *Imputation*, and *Workflow: Xenium × Visium*.
- *Appendices*: including *Acknowledgments*, *Related resources*, *Session information*, and *Citation*.

### R-Python interoperability

The OSTA book is developed primarily using the R programming language, and the majority of the reproducible code examples are run in R, using R packages available from either Bioconductor or CRAN. However, we also include several chapters demonstrating interoperability with Python, in order to demonstrate how to include methods available as Python packages within primarily R-based workflows. These chapters use *reticulate* [24] and/or *basilisk* [25] to set up and manage Python environments, and use Python packages available from PyPI for the analyses. The code examples demonstrate how to convert data objects between R (*SpatialExperiment* [10]) and Python (*AnnData* [11]) using the *zellkonverter* [18] package; alternatively, *anndataR* [17] could also be used. These examples are intended to give readers a starting point for building extended workflows that seamlessly integrate both R and Python-based tools.

### Code and software availability

The OSTA book is freely accessible from the Bioconductor website at https://bioconductor.org/books/OSTA/, and the source code is available from Bioconductor as well as GitHub at https://github.com/lmweber/OSTA. Software packages used for data analyses within the reproducible code examples are available from Bioconductor, CRAN, and PyPI.

## Supplementary Material

Supplement 1

## Figures and Tables

**Figure 2:**
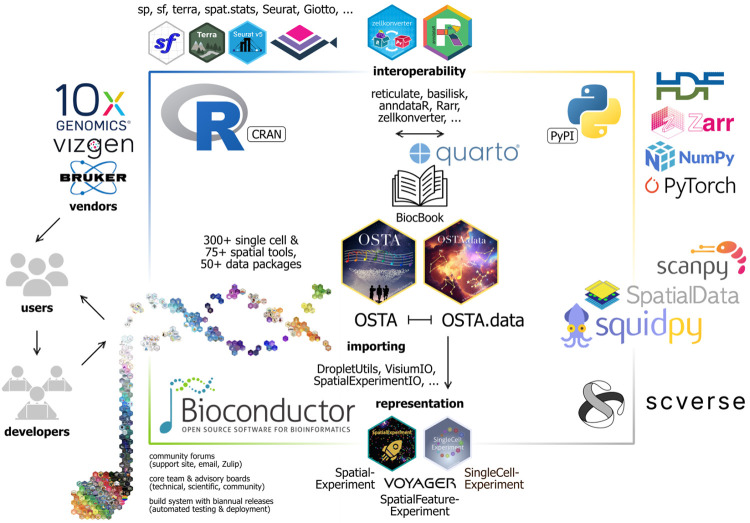
Schematic illustrating how OSTA fits within the Bioconductor and wider analysis frameworks and ecosystems in R and Python for spatial transcriptomics data. Data analysis ecosystems comprise tools from many developers and aim to be interoperable, extensible, and adaptable as biological data and computational methods evolve, in addition to offering higher-level supporting infrastructure. Bioconductor offers a suite of software and data packages for single-cell and spatial omics data analysis; project-wide hallmarks include community forums, the Bioconductor Core Team and advisory boards, and an automated build testing system. OSTA relies on various tools for importing and representing data, for rendering and deployment, as well as software that enables interoperability with Python (e.g. data object conversion and running Python code). R-based frameworks, including additional standalone solutions such as *Seurat* [13] and *Giotto Suite* [14], provide access to extensive R packages from the Comprehensive R Archive Network (CRAN) implementing advanced statistical methods (e.g. spatial statistics and linear modeling) and graphical visualization tools, while Python offers rich infrastructure for, in particular, image analysis and machine learning-based applications, as well as frameworks native to the *scverse* ecosystem such as *Squidpy* [27]. In general, technological vendors act as data generators, while users receive data and aim to output research; users may also become developers who, in turn, contribute to the data analysis ecosystems that supply users with the tools and support needed to analyze their data.

## Data Availability

Datasets used within the reproducible code examples can be freely downloaded and accessed programmatically using functions provided in a companion Bioconductor package *OSTA.data* [26].

## Abbreviations:

SNSF: Swiss National Science Foundation
CoG: Consolidator Grant
DAF: Donor-Advised Fund
DECRA: Discovery Early Career Researcher Award
NIH: National Institutes of Health
NIMH: National Institute of Mental Health
NIGMS: National Institute of General Medical Sciences
NHGRI: National Human Genome Research Institute
NSF: National Science Foundation
