# Supplementary material for: Orchestrating Spatial Transcriptomics Analysis with Bioconductor
Source: bioRxiv. 2025 Nov 21:2025.11.20.688607. Preprint. [Version 1] doi: 10.1101/2025.11.20.688607 (PMC12667768; doi:10.1101/2025.11.20.688607)
Supplement: Supplement 1 [file media-1.pdf]

Supplement to: Orchestrating Spatial Transcriptomics Analysis with Bioconductor

Helena L. Crowell[1,*,✉], Yixing Dong[2,3,*], Ilaria Billato[4], Peiying Cai[5,6], Martin Emons[5,6], Samuel Gunz[5,6], Boyi Guo[7], Mengbo Li[8,9,10], Alexandru Mahmoud[11], Artür Manukyan[12], Hervé Pagès[13], Pratibha Panwar[14,15,16], Shreya Rao[14,15,17], Callum J. Sargeant[8], Lori Shepherd Kern[18], Marcel Ramos[19,20], Jieran Sun[2,3], Michael Totty[21], Vincent J. Carey[11], Yunshun Chen[8,9,10], Leonardo Collado-Torres[21,22,23], Shila Ghazanfar[14,15,16], Kasper D. Hansen[21,24,25], Keri Martinowich[22,26,27,28], Kristen R. Maynard[22,26,27], Ellis Patrick[14,15,16,17], Dario Righelli[29], Davide Risso[30,31], Simone Tiberi[32], Levi Waldron[19,20], Raphael Gottardo[2,3,33,†,✉], Mark D. Robinson[5,6,†,✉], Stephanie C. Hicks[21,25,34,35,†,✉], and Lukas M. Weber[36,†,✉]

[1]National Center for Genomic Analysis, Barcelona, Spain. [2]Biomedical Data Science Center, Lausanne University Hospital, Lausanne, Switzerland. [3]University of Lausanne, Lausanne, Switzerland. [4]Department of Biology, University of Padova, Padova, Italy. [5]Department of Molecular Life Sciences, University of Zurich, Zurich, Switzerland. [6]Swiss Institute of Bioinformatics, Zurich, Switzerland. [7]Division of Biostatistics, Department of Population Health Sciences, University of Utah, Salt Lake City, UT, United States. [8]Bioinformatics and Computational Biology Division, Walter and Eliza Hall Institute of Medical Research, Parkville, VIC, Australia. [9]ACRF Cancer Biology and Stem Cells Division, Walter and Eliza Hall Institute of Medical Research, Parkville, VIC, Australia. [10]Department of Medical Biology, The University of Melbourne, Parkville, VIC, Australia. [11]Channing Division of Network Medicine, Mass General Brigham, Boston, MA, United States. [12]Berlin Institute for Medical Systems Biology, Max-Delbrück-Center for Molecular Medicine in the Helmholtz Association, Berlin, Germany. [13]Fred Hutch Cancer Center, Seattle, WA, United States. [14]School of Mathematics and Statistics, The University of Sydney, Camperdown, NSW, Australia. [15]Sydney Precision Data Science Centre, The University of Sydney, Camperdown, NSW, Australia. [16]Charles Perkins Centre, The University of Sydney, Camperdown, NSW, Australia. [17]Centre for Cancer Research, The Westmead Institute for Medical Research, The University of Sydney, Camperdown, NSW, Australia. [18]Roswell Park Comprehensive Cancer Center, Buffalo, NY, United States. [19]Institute for Implementation Science in Population Health, City University of New York Graduate School of Public Health and Health Policy, New York, NY, United States. [20]Department of Epidemiology and Biostatistics, City University of New York Graduate School of Public Health and Health Policy, New York, NY, United States. [21]Department of Biostatistics, Johns Hopkins Bloomberg School of Public Health, Baltimore, MD, United States. [22]Lieber Institute for Brain Development, Johns Hopkins Medical Campus, Baltimore, MD, United States. [23]Center for Computational Biology, Johns Hopkins University, Baltimore, MD, United States. [24]Department of Genetic Medicine, Johns Hopkins School of Medicine, Baltimore, United States. [25]Department of Biomedical Engineering, Johns Hopkins University, Baltimore, MD, United States. [26]Department of Psychiatry and Behavioral Sciences, Johns Hopkins School of Medicine, Baltimore, MD, United States. [27]Solomon H. Snyder Department of Neuroscience, Johns Hopkins School of Medicine, Baltimore, MD, United States. [28]Johns Hopkins Kavli Neuroscience Discovery Institute, Baltimore, MD, United States. [29]Department of Electrical Engineer and Information Technology, University of Naples "Federico II", Naples, Italy. [30]Department of Statistical Sciences, University of Padova, Padova, Italy. [31]Padua Center for Network Medicine, University of Padova, Padova, Italy. [32]Department of Statistical Sciences, University of Bologna, Bologna, Italy. [33]School of Life Sciences, Ecole Polytechnique Fédérale de Lausanne, Lausanne, Switzerland. [34]Center for Computational Biology, Johns Hopkins University, Baltimore, MD, United States. [35]Malone Center for Engineering in Healthcare, Johns Hopkins University, Baltimore, MD, United States. [36]Department of Biostatistics, Boston University School of Public Health, Boston, MA, United States. * These authors share first authorship. † These authors share senior authorship. *Additional details on author contributions and order are provided under Author Contributions.* ✉ Correspondence: `helena@crowell.eu`, `Raphael.Gottardo@chuv.ch`, `mark.robinson@mls.uzh.ch`, `shicks19@jhu.edu`, `lmweber@bu.edu`

November 20, 2025

Contents

Supplementary Figures 3
- Figure S1: Software dynamics . . . 3
- Figure S2: Publication dynamics . . . 4

Supplementary Tables 5
- Table S1: OSTA references . . . 5

References 6

Supplementary Documents 10
- Workflow: Visium CRC . . . 10
- Workflow: Visium HD . . . 24
- Workflow: Xenium . . . 48
- Workflow: CosMx . . . 58

Supplementary Documents were built separately, without rendering the book in its entirety. Cross-chapter references in individual workflow chapters are therefore inaccurate.

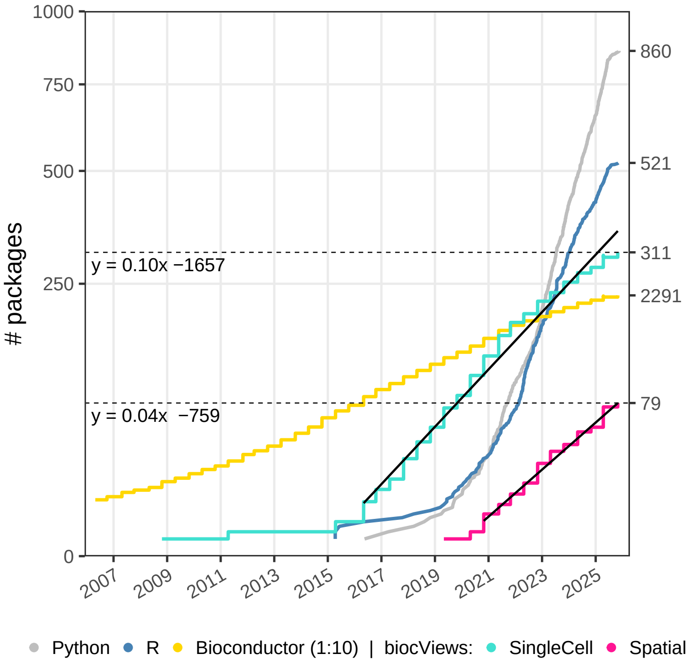

Figure S1: Dynamics of spatial transcriptomics analysis methods in R and Python, and Bioconductor software. Cumulative number of methods published for spatial transcriptomics data analysis, in R (blue) and Python (gray), sourced from [1]; and number of Bioconductor software packages, stratified by *biocViews* categories [2] specified by their authors, available for *SingleCell* (cyan) and *Spatial* (pink) analyses, and all categories (yellow; scaled by a factor of 0.1 for clearer visualization). Right-hand side tick marks indicate the number of methods/packages available at the snapshot date. Black lines and formulas indicate linear model fits for *SingleCell* and *Spatial* *biocViews*, with slopes corresponding to the average number of packages added per day. Dashed lines indicate the current number of packages available for *SingleCell* and *Spatial* *biocViews*, as of Bioconductor release version 3.22. Approximately 36.5 *SingleCell* and 14.6 *Spatial* packages are added to Bioconductor each year (slope $\times$ 365.25), with a delay in growth onset of around 4.5 years (difference in x-axis intercepts). The number of R/Python methods corresponds to publications, and Bioconductor statistics were retrieved using *BiocPkgTools* [3], representing a live indication of usable software. [*Data points are 6-monthly; y-scale is square root transformed; snapshot date: November 20, 2025.*]

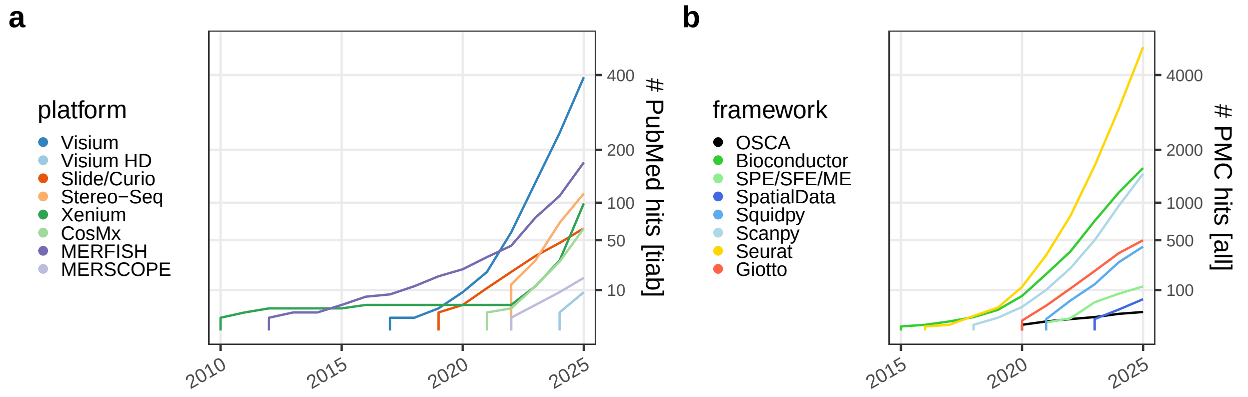

Figure S2: Dynamics of different spatial transcriptomics technological platform and analysis framework mentions in publications. **(a)** Cumulative number of PubMed publication entries that mention specific technological platforms in their title or abstract (*tiab*). Slide/Curio includes Slide-seq, Slide-seqV2, and Curio Seeker. **(b)** Cumulative number of PubMed Central (PMC) publication entries that mention specific analysis frameworks, in addition to the terms "spatial transcriptomics" or "spatially-resolved transcriptomics", in their full text (*all*). SPE/SFE/ME includes *SpatialExperiment*, *SpatialFeatureExperiment*/*Voyager*, and *MoleculeExperiment*. OSCA = *Orchestrating Single-Cell Analysis with Bioconductor*. [*Data points are yearly; y-scale is square root transformed; searches were carried out case-insensitive; snapshot date: November 20, 2025.*]

**Biotechnology**		
ST		[4]
Stereo-seq	STOmics	
Slide-seqV2		[5]
Visium	10x	
Visium HD	10x	
Xenium	10x	
CosMx	Bruker	[6]
MERSCOPE	Vizgen	
MERFISH		[7]
**Infrastructure**		
SpatialExperiment	Bioc	[8]
MoleculeExperiment	Bioc	[9]
SingleCellExperiment	Bioc	[10]
SpatialFeatureExperiment	Bioc	[11]
iSEE	Bioc	[12]
Seurat	GitHub	[13]
VoltRon	GitHub	[14]
spatialGE	GitHub	[15]
Giotto Suite	GitHub	[16]
**Interoperability**		
Rarr	Bioc	[17]
alabaster	Bioc	[18]
basilisk	Bioc	[19]
anndataR	Bioc	[20]
zellkonverter	Bioc	[21]
reticulate	CRAN	[22]
**Datasets**		
mouse brain (Cos)	Bruker	
human brain (Vis)	10x	[23]
human breast cancer (Vis, Xen)	10x	[24]
human CRC (Vis/HD, Xen)	10x	[25]
axolotl brain (Stereo-seq)	STOmics	[26]
human type I diabetes (IMC)		[27]
STexampleData	Bioc	[8]
spatialLIBD	Bioc	[28]
OSTA.data	Bioc	[29]

**Importing**		
VisiumIO	Bioc	
XeniumIO	Bioc	
SpatialExperimentIO	Bioc	
**Preprocessing & quality control**		
stPipe	Bioc	[30]
Rsubread	Bioc	[31]
scater	Bioc	[32]
SpaNorm	Bioc	[33]
SpotSweeper	Bioc	[34]
SpaceTrooper	Bioc	
**Decon., dim. reduction & clustering**		
SpatialDecon	Bioc	
CARDspa	Bioc	[35]
spacexr	Bioc	[36]
BANKSY	Bioc	[37]
BayesSpace	Bioc	[38]
**Neighborhoods & cell-cell comm.**		
RANN	CRAN	
scider	Bioc	[39]
hoodscanR	Bioc	[40]
imcRtools	Bioc	[41]
mistyR	Bioc	[42]
CCPlotR	Bioc	[43]
**Feature sets, selection & testing**		
msigdbr	Bioc	
AUCell	Bioc	[44]
scran	Bioc	[45]
nnSVG	Bioc	[46]
DESpace	Bioc	[47]
spatialDE	Bioc	[48]
**Advanced topics***		
sf	CRAN	[49]
sp	CRAN	[50]
spat.stat	CRAN	[51]
spatialFDA	Bioc	
Statial	Bioc	[52]
spicyR	Bioc	[53]
sosta	Bioc	[54]
pasta	GitHub	[55]

Table S1: Overview of spatial transcriptomics technologies, datasets, and methods that are either mentioned or used throughout the OSTA book. Methods are restricted to software (cursive) with an R implementation; if available, source (company name or code repository) and reference are also listed. *Includes chapters on spatial statistics, differential spatial patterns, differential colocalization, and structure-based analysis. [Bioc = Bioconductor, 10x = 10x Genomics, Vis = Visium, HD = Visium HD, Cos = CosMx, Xen = Xenium, IMC = imaging mass cytometry, CRC = colorectal carcinoma, decon. = deconvolution, dim. reduction = dimensionality reduction, cell-cell comm. = cell-cell communication.]

References

1. Moses L and Pachter L. Museum of spatial transcriptomics. Nature Methods 2022; 19:534–46. DOI: 10.1038/s41592-022-01409-2
2. Carey VJ, Harshfield BJ, Falcon S, Arora S, and Shepherd L. biocViews: Categorized views of R package repositories. R package 2025. DOI: 10.18129/B9.bioc.biocViews
3. Su S, Shepherd L, Ramos M, Ernst FG, Wokaty J, Soneson C, Morgan M, Carey V, and Davis S. BiocPkgTools: Collection of simple tools for learning about Bioconductor Packages. R package 2025. DOI: 10.18129/B9.bioc.BiocPkgTools
4. Ståhl PL, Salmén F, Vickovic S, Lundmark A, Navarro JF, Magnusson J, Giacomello S, Asp M, Westholm JO, Huss M, Mollbrink A, Linnarsson S, Codeluppi S, Borg Å, Pontén F, Costea PI, Sahlén P, Mulder J, Bergmann O, Lundeberg J, and Frisén J. Visualization and analysis of gene expression in tissue sections by spatial transcriptomics. Science 2016; 353(6294):78–82. DOI: 10.1126/science.aaf2403
5. Stickels RR, Murray E, Kumar P, Li J, Marshall JL, Bella DJD, Arlotta P, Macosko EZ, and Chen F. Highly sensitive spatial transcriptomics at near-cellular resolution with Slide-seqV2. Nature Biotechnology 2021; 39:313–9. DOI: 10.1038/s41587-020-0739-1
6. He S, Bhatt R, Brown C, Brown EA, Buhr DL, Chantranuvatana K, Danaher P, Dunaway D, Garrison RG, Geiss G, Gregory MT, Hoang ML, Khafizov R, Killingbeck EE, Kim D, Kim TK, Kim Y, Klock A, Korukonda M, Kutchma A, Lewis ZR, Liang Y, Nelson JS, Ong GT, Perillo EP, Phan JC, Phan-Everson T, Piazza E, Rane T, Reitz Z, Rhodes M, Rosenbloom A, Ross D, Sato H, Wardhani AW, Williams-Wietzikoski CA, Wu L, and Beechem JM. High-plex imaging of RNA and proteins at subcellular resolution in fixed tissue by spatial molecular imaging. Nature Biotechnology 2022; 40:1794–806. DOI: 10.1038/s41587-022-01483-z
7. Chen KH, Boettiger AN, Moffitt JR, Wang S, and Zhuang X. Spatially resolved, highly multiplexed RNA profiling in single cells. Science 2015; 348(6233). DOI: 10.1126/science.aaa6090
8. Righelli D, Weber LM, Crowell HL, Pardo B, Collado-Torres L, Ghazanfar S, Lun ATL, Hicks SC, and Risso D. SpatialExperiment: infrastructure for spatially-resolved transcriptomics data in R using Bioconductor. Bioinformatics 2022; 38(11):3128–31. DOI: 10.1093/bioinformatics/btac299
9. Peters Couto BZ, Robertson N, Patrick E, and Ghazanfar S. MoleculeExperiment enables consistent infrastructure for molecule-resolved spatial omics data in Bioconductor. Bioinformatics 2023; 39(9). DOI: 10.1093/bioinformatics/btad550
10. Amezquita RA, Lun ATL, Becht E, Carey VJ, Carpp LN, Geistlinger L, Marini F, Rue-Albrecht K, Risso D, Soneson C, Waldron L, Pagès H, Smith ML, Huber W, Morgan M, Gottardo R, and Hicks SC. Orchestrating single-cell analysis with Bioconductor. Nature Methods 2020; 17:137–45. DOI: 10.1038/s41592-019-0654-x
11. Moses L, Einarsson PH, Jackson K, Luebbert L, Booeshaghi AS, Antonsson S, Bray N, Melsted P, and Pachter L. Voyager: exploratory single-cell genomics data analysis with geospatial statistics. bioRxiv 2023. DOI: 10.1101/2023.07.20.549945
12. Rue-Albrecht K, Marini F, Soneson C, and Lun AT. iSEE: Interactive SummarizedExperiment Explorer. F1000Research 2018; 7. DOI: 10.12688/f1000research.14966.1
13. Hao Y, Stuart T, Kowalski MH, Choudhary S, Hoffman P, Hartman A, Srivastava A, Molla G, Madad S, Fernandez-Granda C, and Satija R. Dictionary learning for integrative, multimodal and scalable single-cell analysis. Nature Biotechnology 2024; 42:293–304. DOI: 10.1038/s41587-023-01767-y
14. Manukyan A, Bahry E, Wyler E, Becher E, Pascual-Reguant A, Plumbom I, Dikmen HO, Elezkurtaj S, Conrad T, Altmüller J, Hauser AE, Hocke A, Radbruch H, Schmidt D, Landthaler M, and Akalin A. VoltRon: A Spatial Omics Analysis Platform for Multi-Resolution and Multi-omics Integration using Image Registration. bioRxiv 2023. DOI: 10.1101/2023.12.15.571667

15. Ospina OE, Wilson CM, Soupir AC, Berglund A, Smalley I, Tsai KY, and Fridley BL. spatialGE: quantification and visualization of the tumor microenvironment heterogeneity using spatial transcriptomics. Bioinformatics 2022; 38:2645–7. DOI: 10.1093/bioinformatics/btac145

16. Chen JG, Chávez-Fuentes JC, O'Brien M, Xu J, Ruiz EC, Wang W, Amin I, Sheridan JP, Shin SC, Hasyagar SV, Sarfraz I, Guckhool P, Sistig A, Jarzabek V, Yuan GC, and Dries R. Giotto Suite: a multiscale and technology-agnostic spatial multiomics analysis ecosystem. Nature Methods 2025; 22:2052–64. DOI: 10.1038/s41592-025-02817-w

17. Smith M and Gruson H. Rarr: Read Zarr Files in R. R package 2023. DOI: 10.18129/B9.bioc.Rarr

18. Lun A. alabaster.base: Save Bioconductor Objects to File. R package 2023. DOI: 10.18129/B9.bioc.alabaster.base

19. Lun ATL. basilisk: a Bioconductor package for managing Python environments. Journal of Open Source Software 2022; 7(79). DOI: 10.21105/joss.04742

20. Deconinck L, Zappia L, Cannoodt R, Morgan M, scverse core, Virshup I, Sang-Aram C, Bredikhin D, Schilder B, Seurinck R, and Saeys Y. anndataR improves interoperability between R and Python in single-cell transcriptomics. bioRxiv 2025. DOI: 10.1101/2025.08.18.669052

21. Zappia L, Lun A, Kamm J, Cannoodt R, Hoffman G, and Cmero M. zellkonverter. R package version 1.18.0 2025. DOI: 10.18129/B9.bioc.zellkonverter

22. Ushey K, Allaire JJ, and Tang Y. reticulate: Interface to 'Python'. R package 2017. DOI: 10.32614/CRAN.package.reticulate

23. Maynard KR, Collado-Torres L, Weber LM, Uytingco C, Barry BK, Williams SR, II JLC, Tran MN, Besich Z, Tippani M, Chew J, Yin Y, Kleinman JE, Hyde TM, Rao N, Hicks SC, Martinowich K, and Jaffe AE. Transcriptome-scale spatial gene expression in the human dorsolateral prefrontal cortex. Nature Neuroscience 2021; 24:425–36. DOI: 10.1038/s41593-020-00787-0

24. Janesick A, Shelansky R, Gottscho AD, Wagner F, Williams SR, Rouault M, Beliakoff G, Morrison CA, Oliveira MF, Sicherman JT, Kohlway A, Abousoud J, Drennon TY, Mohabbat SH, 10x Development Teams, and Taylor SEB. High resolution mapping of the tumor microenvironment using integrated single-cell, spatial and in situ analysis. Nature Communications 2023; 14. DOI: 10.1038/s41467-023-43458-x

25. de Oliveira MF, Romero JP, Chung M, Williams SR, Gottscho AD, Gupta A, Pilipauskas SE, Mohabbat S, Raman N, Sukovich DJ, Patterson DM, Visium HD Development Team, and Taylor SEB. High-definition spatial transcriptomic profiling of immune cell populations in colorectal cancer. Nature Genetics 2025; 57:1512–23. DOI: 10.1038/s41588-025-02193-3

26. Wei X, Fu S, Li H, Liu Y, Wang S, Feng W, Yang Y, Liu X, Zeng YY, Cheng M, Lai Y, Qiu X, Wu L, Zhang N, Jiang Y, Xu J, Su X, Peng C, Han L, Lou WPK, Liu C, Yuan Y, Ma K, Yang T, Pan X, Gao S, Chen A, Esteban MA, Yang H, Wang J, Fan G, Liu L, Chen L, Xu X, Fei JF, and Gu Y. Single-cell Stereo-seq reveals induced progenitor cells involved in axolotl brain regeneration. Science 2022; 377(6610). DOI: 10.1126/science.abp9444

27. Damond N, Engler S, Zanotelli VR, Schapiro D, Wasserfall CH, Kusmartseva I, Nick HS, Thorel F, Herrera PL, Atkinson MA, and Bodenmiller B. A Map of Human Type 1 Diabetes Progression by Imaging Mass Cytometry. Cell Metabolism 2019; 29(3):755–68. DOI: 10.1016/j.cmet.2018.11.014

28. Pardo B, Spangler A, Weber LM, Page SC, Hicks SC, Jaffe AE, Martinowich K, Maynard KR, and Collado-Torres L. spatialLIBD: an R/Bioconductor package to visualize spatially-resolved transcriptomics data. BMC Genomics 2022; 23. DOI: 10.1186/s12864-022-08601-w

29. Dong YE, Crowell HL, and Carey V. OSTA.data. R package version 1.2.0 2025. DOI: 10.18129/B9.bioc.OSTA.data

30. Xu Y, Sargeant CJ, You Y, You Y, Su S, Wang C, Tian L, Chen Y, and Ritchie ME. stPipe: A flexible and streamlined R/Bioconductor pipeline for preprocessing sequencing-based spatial transcriptomics data. bioRxiv 2025. DOI: 10.1101/2025.04.16.649254

31. Liao Y, Smyth GK, and Shi W. The R package Rsubread is easier, faster, cheaper and better for alignment and quantification of RNA sequencing reads. Nucleic Acids Research 2019; 47(8):e47. DOI: 10.1093/nar/gkz114

32. McCarthy DJ, Campbell KR, Lun ATL, and Wills QF. Scater: pre-processing, quality control, normalization and visualization of single-cell RNA-seq data in R. Bioinformatics 2017; 33(8):1179–86. DOI: 10.1093/bioinformatics/btw777

33. Salim A, Bhuva DD, Chen C, Tan CW, Yang P, Davis MJ, and Yang JYH. SpaNorm: spatially-aware normalization for spatial transcriptomics data. Genome Biology 2025; 26. DOI: 10.1186/s13059-025-03565-y

34. Totty M, Hicks SC, and Guo B. SpotSweeper: spatially aware quality control for spatial transcriptomics. Nature Methods 2025; 22:1520–30. DOI: 10.1038/s41592-025-02713-3

35. Ma Y and Zhou X. Spatially informed cell-type deconvolution for spatial transcriptomics. Nature Biotechnology 2022 Sep; 40:1349–59. DOI: 10.1038/s41587-022-01273-7

36. Cable DM, Murray E, Zou LS, Goeva A, Macosko EZ, Chen F, and Irizarry RA. Robust decomposition of cell type mixtures in spatial transcriptomics. Nature Biotechnology 2022; 40:517–26. DOI: 10.1038/s41587-021-00830-w

37. Singhal V, Chou N, Lee J, Yue Y, Liu J, Chock WK, Lin L, Chang YC, Teo EML, Aow J, Lee HK, Chen KH, and Prabhakar S. BANKSY unifies cell typing and tissue domain segmentation for scalable spatial omics data analysis. Nature Genetics 2024; 56:431–41. DOI: 10.1038/s41588-024-01664-3

38. Zhao E, Stone MR, Ren X, Guenthoer J, Smythe KS, Pulliam T, Williams SR, Uytingco CR, Taylor SEB, Nghiem P, Bielas JH, and Gottardo R. Spatial transcriptomics at subspot resolution with BayesSpace. Nature Biotechnology 2021; 39:1375–84. DOI: 10.1038/s41587-021-00935-2

39. Li M, Liu N, Nguyen QH, and Chen Y. Preserving tissue structure through density-based spatial analysis with scider. bioRxiv 2025. DOI: 10.1101/2025.09.11.675745

40. Liu N, Martin J, Bhuva DD, Chen J, Li M, Lee SC, Kharbanda M, Cheng J, Mohamed A, Kulasinghe A, Polo JM, Chen Y, Tan CW, and Davis MJ. hoodscanR: profiling single-cell neighborhoods in spatial transcriptomics data. bioRxiv 2025. DOI: 10.1101/2024.03.26.586902

41. Windhager J, Zanotelli VRT, Schulz D, Meyer L, Daniel M, Bodenmiller B, and Eling N. An end-to-end workflow for multiplexed image processing and analysis. Nature Protocols 2023; 18:3565–613. DOI: 10.1038/s41596-023-00881-0

42. Tanevski J, Flores ROR, Gabor A, Schapiro D, and Saez-Rodriguez J. Explainable multiview framework for dissecting spatial relationships from highly multiplexed data. Genome Biology 2022; 23:97. DOI: 10.1186/s13059-022-02663-5

43. Ennis S, Ó Broin P, and Szegezdi E. CCPlotR: an R package for the visualization of cell-cell interactions. Bioinformatics Advances 2023; 3:vbad130. DOI: 10.1093/bioadv/vbad130

44. Aibar S, González-Blas CB, Moerman T, Huynh-Thu VA, Imrichova H, Hulselmans G, Rambow F, Marine JC, Geurts P, Aerts J, Oord J van den, Atak ZK, Wouters J, and Aerts S. SCENIC: single-cell regulatory network inference and clustering. Nature Methods 2017; 14:1083–6. DOI: 10.1038/nmeth.4463

45. Lun AT, McCarthy DJ, and Marioni JC. A step-by-step workflow for low-level analysis of single-cell RNA-seq data with Bioconductor. F1000Research 2016; 5. DOI: 10.12688/f1000research.9501.2

46. Weber LM, Saha A, Datta A, Hansen KD, and Hicks SC. nnSVG for the scalable identification of spatially variable genes using nearest-neighbor Gaussian processes. Nature Communications 2023; 14. DOI: 10.1038/s41467-023-39748-z

47. Cai P, Robinson MD, and Tiberi S. DESpace: spatially variable gene detection via differential expression testing of spatial clusters. Bioinformatics 2024; 40(2). DOI: 10.1093/bioinformatics/btae027

48. Svensson V, Teichmann SA, and Stegle O. SpatialDE: identification of spatially variable genes. Nature Methods 2018; 15:343–6. DOI: 10.1038/nmeth.4636

49. Pebesma E. Simple features for R: Standardized support for spatial vector data. The R Journal 2018; 10:439–46. DOI: 10.32614/rj-2018-009

50. Pebesma E and Bivand RS. Classes and Methods for Spatial Data: the sp Package. The Newsletter of the R Project 2005; 5

51. Baddeley A and Turner R. spatstat: An R Package for Analyzing Spatial Point Patterns. Journal of Statistical Software 2005; 12(6):1–42. DOI: 10.18637/jss.v012.i06

52. Ameen F, Robertson N, Lin DM, Ghazanfar S, and Patrick E. Kontextual: Reframing analysis of spatial omics data reveals consistent cell relationships across images. bioRxiv 2024. DOI: 10.1101/2024.09.03.611109

53. Canete NP, Iyengar SS, Ormerod JT, Baharlou H, Harman AN, and Patrick E. spicyR: spatial analysis of in situ cytometry data in R. Bioinformatics 2022; 38(11):3099–105. DOI: 10.1093/bioinformatics/btac268

54. Gunz S, Crowell HL, and Robinson MD. Analysis of anatomical multi-cellular structures from spatial omics data using sosta. bioRxiv 2025. DOI: 10.1101/2025.10.13.682065

55. Emons M, Gunz S, Crowell HL, Mallona I, Furrer R, and Robinson MD. Harnessing the Potential of Spatial Statistics for Spatial Omics Data with pasta. Nucleic Acids Research 2025; 53:gkaf870. DOI: 10.1038/s41467-022-28020-5

Workflow: Visium CRC

Preamble

Introduction

In this demo, we will be analyzing Visium data on a human colorectal cancer biopsy from de Oliveira et al. (2025). Rather than recapitulating all possible analyses, our goal is to highlight those that might be of particular interest in the context of these data.

Dependencies

```
library(osfr)
library(scran)
library(scater)
library(igraph)
library(AUCell)
library(scuttle)
library(spacexr)
library(msigdbr)
library(VisiumIO)
library(jsonlite)
library(ggspavis)
library(pheatmap)
library(patchwork)
library(OSTA.data)
library(BiocParallel)
library(DropletUtils)
library(SpatialExperiment)
# specify whether/how to
# perform parallelization
bp <- MulticoreParam(th <- 4)
# set seed for random number generation
# in order to make results reproducible
set.seed(194849)
```

Data import

```
# retrieve dataset from OSF repo
id <- "Visium_HumanColon_Oliveira"
pa <- OSTA.data_load(id)
```

```r
dir.create(td <- tempfile())
unzip(pa, exdir=td)

# read into 'SpatialExperiment'
obj <- TENxVisium(
    spacerangerOut=file.path(td, "outs"),
    format="h5",
    images="lowres")
(spe <- import(obj))
```

```
## class: SpatialExperiment
## dim: 18085 4269
## metadata(2): resources spatialList
## assays(1): counts
## rownames(18085): ENSG00000187634 ENSG00000188976 ... ENSG00000198695
##   ENSG00000198727
## rowData names(3): ID Symbol Type
## colnames(4269): AACAATGTGCTCCGAG-1 AACACCATTCGCATAC-1 ...
##   TGTTGGTGCGGAATCA-1 TGTTGGTGGACTCAGG-1
## colData names(4): in_tissue array_row array_col sample_id
## reducedDimNames(0):
## mainExpName: Gene Expression
## altExpNames(0):
## spatialCoords names(2) : pxl_col_in_fullres pxl_row_in_fullres
## imgData names(4): sample_id image_id data scaleFactor
```

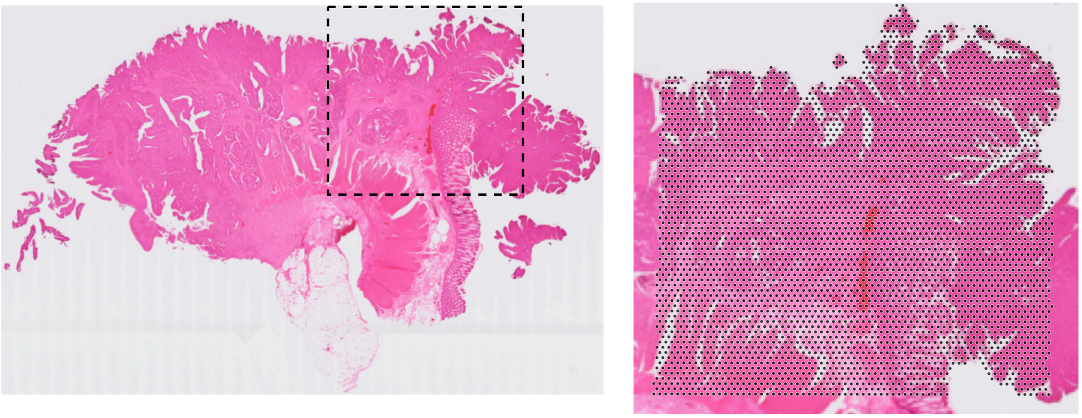

Quality control

```r
# use gene symbols as feature names
rownames(spe) <- make.unique(rowData(spe)$Symbol)
# add per-cell quality control metrics
sub <- list(mt=grep("^MT-", rownames(spe)))
spe <- addPerCellQCMetrics(spe, subsets=sub)
```

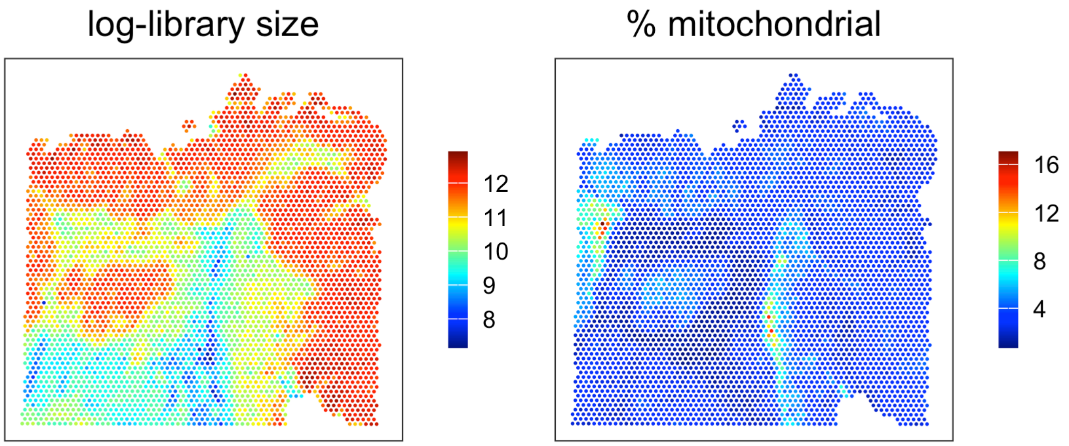


```
# determine outliers via thresholding on MAD from the median
ol <- perCellQCFilters(spe, sub.fields="subsets_mt_percent")
# add results as cell metadata
colData(spe)[names(ol)] <- ol
# tabulate # and % of cells that'd
# be discarded for different reasons
data.frame(
    check.names=FALSE,
    `#`=apply(ol, 2, sum),
    `%`=round(100*apply(ol, 2, mean), 2))
```

```
##                         #     %
## low_lib_size            3  0.07
## low_n_features        636 14.90
## high_subsets_mt_percent 161  3.77
## discard               779 18.25
```

Let's see which spots would be excluded according to the above criteria:

```
lapply(names(ol), \(.)
    plotCoords(spe, annotate=.) + ggtitle(.)) |>
    wrap_plots(nrow=1, guides="collect") &
    guides(col=guide_legend(override.aes=list(size=3))) &
    scale_color_manual("discard", values=c("lavender", "purple")) &
    theme(plot.title=element_text(hjust=0.5), legend.key.size=unit(0, "lines"))
```

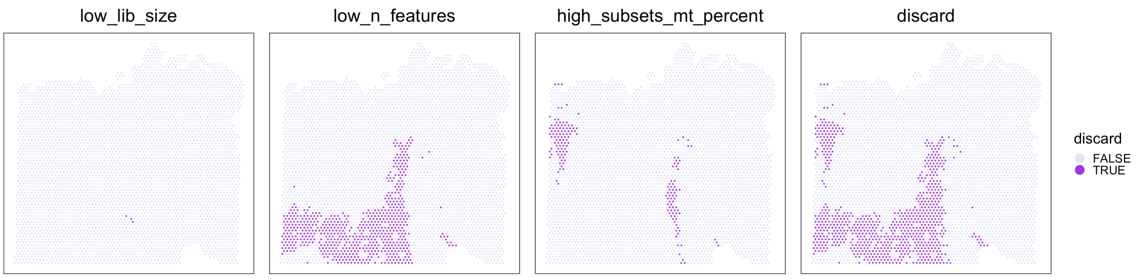

It seems like low-quality spots are highly spatially organized, so that might might encourage us to not remove them, for now. We will see further below how the quality control metrics used here, and spots deemed to be `discarded`, are distributed across (transcription-based) clusters.

Processing

```
# log-library size normalization
spe <- logNormCounts(spe)
# highly variable feature selection
tbl <- modelGeneVar(spe)
sel <- getTopHVGs(tbl, n=2e3)
# principal component analysis
spe <- runPCA(spe, subset_row=sel)
```

Clustering

As an unsupervised approach, we perform shared nearest-neighbor (SNN) graph-based clustering using the Leiden community detection algorithm.

```
# build shared nearest-neighbor (SNN) graph
g <- buildSNNGraph(spe, use.dimred="PCA", type="jaccard")
# cluster via Leiden community detection algorithm
k <- cluster_leiden(g, objective_function="modularity", resolution=0.5)
table(spe$Leiden <- factor(k$membership))
```

```
##
##    1   2   3   4   5   6   7   8   9  10
##  326 504 710 432 732 458 315 171 469 152
```

Deconvolution

In a complementary approach, we deconvolute spot measurements using (annotated) reference single-cell data provided by the authors. Let's first retrieve these data, alongside corresponding cell metadata, which includes low- (`Level1`) and high-resolution (`Level2`) annotations into 10 and 32 subpopulations, respectively.

```
# retrieve dataset from OSF repo
id <- "Chromium_HumanColon_Oliveira"
pa <- OSTA.data_load(id)
dir.create(td <- tempfile())
unzip(pa, exdir=td)
```

```
# read into 'SingleCellExperiment'
fs <- list.files(td, full.names=TRUE)
h5 <- grep("h5$", fs, value=TRUE)
sce <- read10xCounts(h5, col.names=TRUE)
```

```r
# add cell metadata
csv <- grep("csv$", fs, value=TRUE)
cd <- read.csv(csv, row.names=1)
colData(sce)[names(cd)] <- cd[colnames(sce), ]
# use gene symbols as feature names
rownames(sce) <- make.unique(rowData(sce)$Symbol)
# exclude cells deemed to be of low-quality
sce <- sce[, sce$QCFilter == "Keep"]
# tabulate subpopulations
table(sce$Level1)
```

```
##
##               B cells          Endothelial           Fibroblast
##                 33611                 7969                28653
## Intestinal Epithelial              Myeloid             Neuronal
##                 22763                25105                 4199
##         Smooth Muscle              T cells                Tumor
##                 43308                29272                65626
```

Next, we perform deconvolution with *spacexr* (RCTD) (Cable et al. 2022). By default, `runRctd()`'s `rctd_mode="doublet"`, i.e., at most two subpopulations are fit per pixel; here, we set `rctd_mode="full"` in order to allow for an arbitrary number of subpopulations to be fit instead.

Here, we filter the reference data to contain only cells from the same patient, and downsample to retain a limited number of cells per subpopulation. This is not strictly necessary, assuming that clusters are transcriptionally stable across patients, but helps with reducing runtime and memory consumption here.

```r
# prep reference data (Chromium);
# subset cells from same patient
.sce <- sce[, grepl("P2", sce$Patient)]
# downsample to at most 4,000 cells per cluster
cs <- split(seq_len(ncol(.sce)), .sce$Level1)
cs <- lapply(cs, \(.) sample(., min(length(.), 4e3)))
.sce <- .sce[, unlist(cs)]
# run 'RCTD' deconvolution
rctd_data <- createRctd(spe, .sce, cell_type_col="Level1")
(res <- runRctd(rctd_data, max_cores=th, rctd_mode="full"))
```

```
## class: SpatialExperiment
## dim: 9 4269
## metadata(4): spatial_rna config cell_type_info internal_vars
## assays(1): weights
## rownames(9): B cells Endothelial ... T cells Tumor
## rowData names(0):
## colnames(4269): AACAATGTGCTCCGAG-1 AACACCATTCGCATAC-1 ...
##   TGTTGGTGCGGAATCA-1 TGTTGGTGGACTCAGG-1
## colData names(1): sample_id
## reducedDimNames(0):
## mainExpName: NULL
## altExpNames(0):
## spatialCoords names(2) : x y
## imgData names(0):
```

Weights inferred by RCTD should be normalized such that proportions of cell types sum to 1 in each spot:

```r
# scale weights such that they sum to 1
ws <- assay(res)
ws <- sweep(ws, 2, colSums(ws), `/`)
# add proportion estimates as metadata
ws <- data.frame(t(as.matrix(ws)))
colData(spe)[names(ws)] <- ws[colnames(spe), ]
```

For comparison with unsupervised clustering (SNN-based Leiden), we also include assignments we would obtain if we were to assign spots the most frequent label (in terms of deconvolution estimates):

```r
ids <- names(ws)[apply(ws, 1, which.max)]
table(spe$RCTD <- factor(ids), spe$Leiden)
```

```
##
##                          1   2   3   4   5   6   7   8   9  10
##    B.cells               0  42   0   6   0   4   0   0   0   0
##    Endothelial           0 113   0   0   0  23   0   0   0   0
##    Fibroblast            0 271   0   0  11  57 313   0   0   0
##    Intestinal.Epithelial 0   4   0   4   0   0   0   0 466  10
##    Myeloid               0  16   0   0   2   3   0   0   0   0
##    Smooth.Muscle         0   8   0   0   0 362   0   0   0   0
##    T.cells               0  11   0   0   0   3   0   0   0   0
##    Tumor               326  39 710 422 719   6   2 171   3 142
```

We can also compartmentalize the tissue into broad biological compartments; here, by grouping RCTD-based subpopulation assignments into four classes:

```r
lab <- list(
    tum="Tumor",
    epi="Intestinal.Epithelial",
    imm=c("B.cells", "T.cells", "Myeloid"),
    str=c("Endothelial", "Fibroblast", "Smooth.Muscle"))
idx <- match(spe$RCTD, unlist(lab))
lab <- rep.int(names(lab), sapply(lab, length))
table(spe$Domain <- factor(lab[idx]))
```

```
##
##   epi  imm  str  tum
##   484   87 1158 2540
```

Exploratory

Let's visualize deconvolution weights in space, i.e., coloring by the proportion of a given cell type estimated to fall within a given spot:

```r
lapply(names(ws), \(.)
    plotCoords(spe, annotate=.)) |>
    wrap_plots(nrow=3) & theme(
    legend.key.width=unit(0.5, "lines"),
```

```
    legend.key.height=unit(1, "lines")) &
    scale_color_gradientn(colors=pals::jet())
```

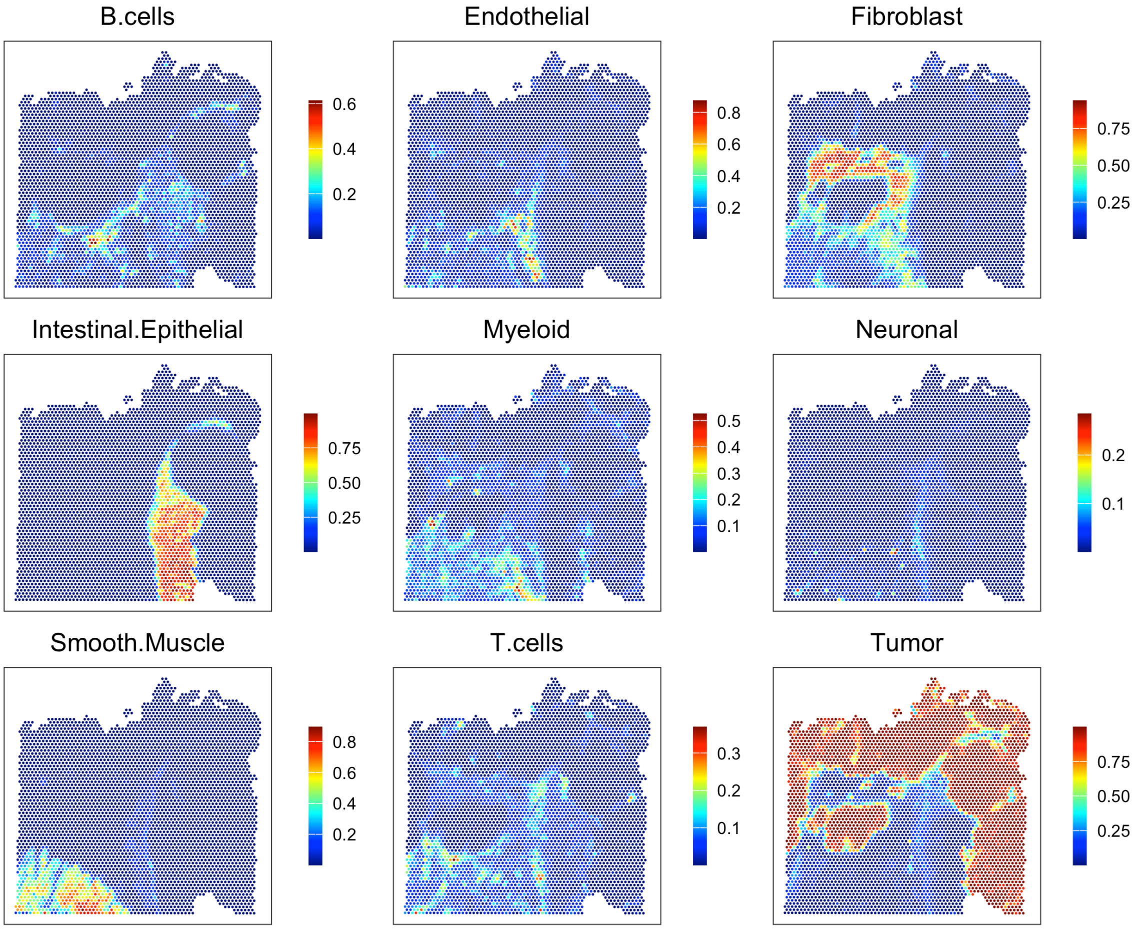


```
lapply(c("Leiden", "Domain", "RCTD"),
    \(.) plotCoords(spe, annotate=.)) |>
    wrap_plots(nrow=1) &
    theme(legend.key.size=unit(0, "lines")) &
    scale_color_manual(values=unname(pals::trubetskoy()))
```

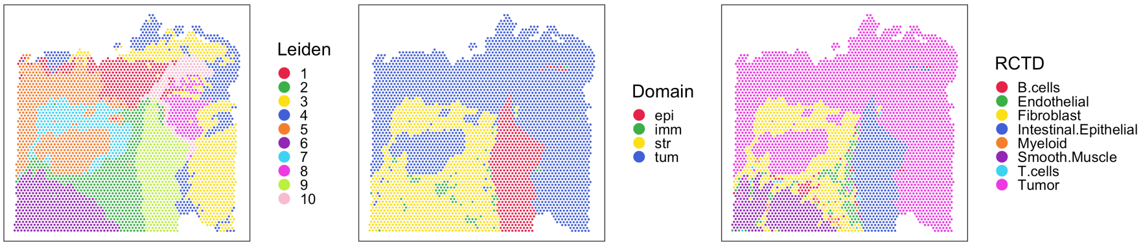

To help characterize subpopulations from unsupervised clustering, we can view their distribution across deconvolution-based clusters and broad domains; e.g., tumor spots are quite

diverse, while smooth muscle spots and (normal) epithelia map almost completely to a single cluster:

```
cd <- data.frame(colData(spe))
df <- as.data.frame(with(cd, table(RCTD, Leiden)))
fd <- as.data.frame(with(cd, table(Domain, Leiden)))
ggplot(df, aes(Freq, RCTD, fill=Leiden)) + ggtitle("RCTD") +
ggplot(fd, aes(Freq, Domain, fill=Leiden)) + ggtitle("Domain") +
plot_layout(nrow=1, guides="collect") &
    labs(x="Proportion", y=NULL) &
    coord_cartesian(expand=FALSE) &
    geom_col(width=1, col="white", position="fill") &
    scale_fill_manual(values=unname(pals::trubetskoy())) &
    theme_minimal() & theme(aspect.ratio=1,
        legend.key.size=unit(2/3, "lines"),
        plot.title=element_text(hjust=0.5))
```

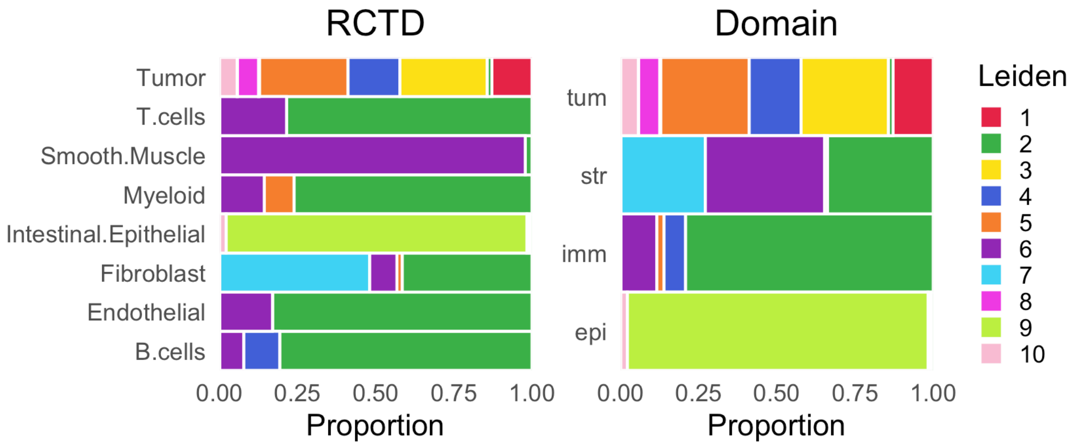

Let's inspect the key drivers of (expression) variability in terms of PCs. Considering clustering and deconvolution results from above, we can see that

- PC1 distinguishes stromal from both normal and malignant epithelia;
- PC2 clearly separates (normal) intestinal epithelium from all else;
- PC3 captures a fibroblast-rich region, and normal epithelia;
- PC5 separates fibroblasts and smooth muscle cells; etc.

```
pcs <- reducedDim(spe, "PCA")
colData(spe)[colnames(pcs)] <- pcs
lapply(colnames(pcs)[seq_len(6)],
    \(.) plotCoords(spe, annotate=.) +
    scale_color_gradientn(., colors=pals::jet())) |>
    wrap_plots(nrow=2) & theme(
        plot.title=element_blank(),
        legend.key.width=unit(0.5, "lines"),
        legend.key.height=unit(1, "lines"))
```

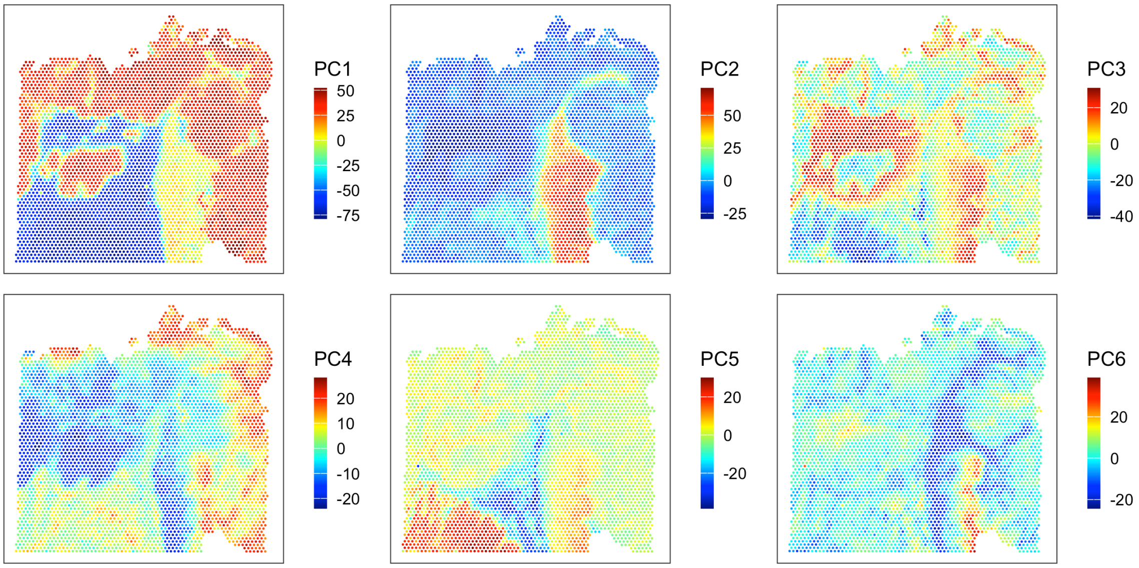

Quality control metrics tend to be low for specific clusters. Their patch-like pattern, in turn, explains the clustering of low-quality spots seen earlier.

```
lapply(c("detected", "log_sum", "subsets_mt_percent"), \(.)
    plotColData(spe, x=., y="Leiden", color_by="discard", point_size=0.1) +
    scale_x_discrete(limits=names(sort(by(spe[[.]], spe$Leiden, median))))) |>
    wrap_plots(nrow=1, guides="collect") &
    scale_color_manual("discard", values=c("lavender", "purple")) &
    guides(col=guide_legend(override.aes=list(alpha=1, size=3))) &
    theme_minimal() & theme(
        panel.grid.minor=element_blank(),
        legend.key.size=unit(0, "lines"))
```

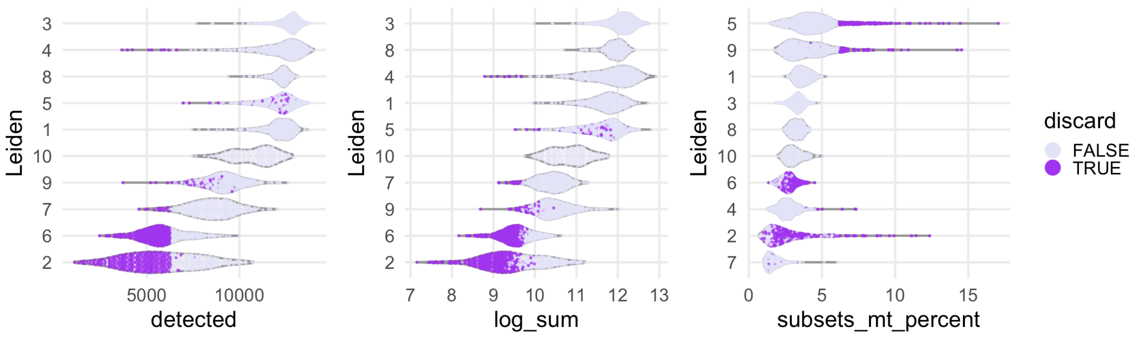

Signatures

Rather than investigating single genes, we can also evaluate the expression of sets of genes (e.g., pathway signatures); e.g., malignant tissue may differ in metabolic activity such as

Note that such gene lists like these may come from many different places - e.g., in-house analyses, other publications matching the research question etc. As such, they may also be read

glycolysis and fatty acid metabolism, or exhibit increased apoptosis (cell death) etc. Here, we retrieve hallmark gene sets for some biological phenomena from MSigDB, using the *msigdbr* package:

```
# retrieve hallmark gene sets from 'MSigDB'
db <- msigdbr(species="Homo sapiens", category="H")
```

```
## Warning: The `category` argument of `msigdbr()` is deprecated as of msigdbr 10.0.0.
## i Please use the `collection` argument instead.
```

```
# get list of gene symbols, one element per set
gs <- split(db$ensembl_gene, db$gs_name)
# simplify set identifiers (drop prefix, use lower case)
names(gs) <- tolower(gsub("HALLMARK_", "", names(gs)))
# how many sets?
length(gs)
```

```
## [1] 50
```

```
# how many genes in each?
range(sapply(gs, length))
```

```
## [1]  32 201
```

Next, we will score these using *AUCell* (Aibar et al. 2017), which works in two steps: (i) rank genes for every observation (here, spots), and (ii) compute AUC values for each gene set. In essence, these represent the fraction of genes (within top-ranked genes; default 5%) that are in a given set; i.e., high values correspond to high activity (in terms of coordinated gene expression).

By definition, `AUCell` yields values in [0,1]. Larger sets (more genes) are more likely to achieve higher scores by chance (e.g., a gene set of *all* genes would score 1 in any dataset). It is thus unfair to compare them directly. However, spatial distribution, correlation, and relative comparisons between subpopulations etc. are still meaningful.

```
# realize (sparse) gene expression matrix
mtx <- as(logcounts(spe), "dgCMatrix")
# use ensembl identifiers as feature names
rownames(mtx) <- rowData(spe)$ID
# build per-spot gene rankings
rnk <- AUCell_buildRankings(mtx, BPPARAM=bp, plotStats=FALSE, verbose=FALSE)
# calculate AUC for each gene set in each spot
auc <- AUCell_calcAUC(geneSets=gs, rankings=rnk, nCores=th, verbose=FALSE)
# add results as spot metadata
colData(spe)[rownames(auc)] <- res <- t(assay(auc))
```

For simplicity, we'll continue investigating only those signatures with the highest score variability across spots:

```
var <- colVars(res) # variance across spots
top <- names(tail(sort(var), 8)) # top sets)
```

To summarize, MYC signalling is absent in stromal regions; the fibroblast ring surrounding a cancerous patch exhibits EMT, angiogenesis, etc.; INFa response and TNFa signalling is patch-like in both stroma and malignant epithelia.

```r
lapply(top, \(.) {
    spe[[.]] <- scale(spe[[.]]) # scaling
    plotCoords(spe, annotate=.) # plotting
}) |>
    # arrange & prettify
    wrap_plots(nrow=2, guides="collect") &
    scale_color_gradientn(
        colors=pals::jet(),
        oob=scales::squish,
        limits=c(-2.5, 2.5)) &
    theme(
        legend.key.width=unit(0.5, "lines"),
        legend.key.height=unit(1, "lines"))
```

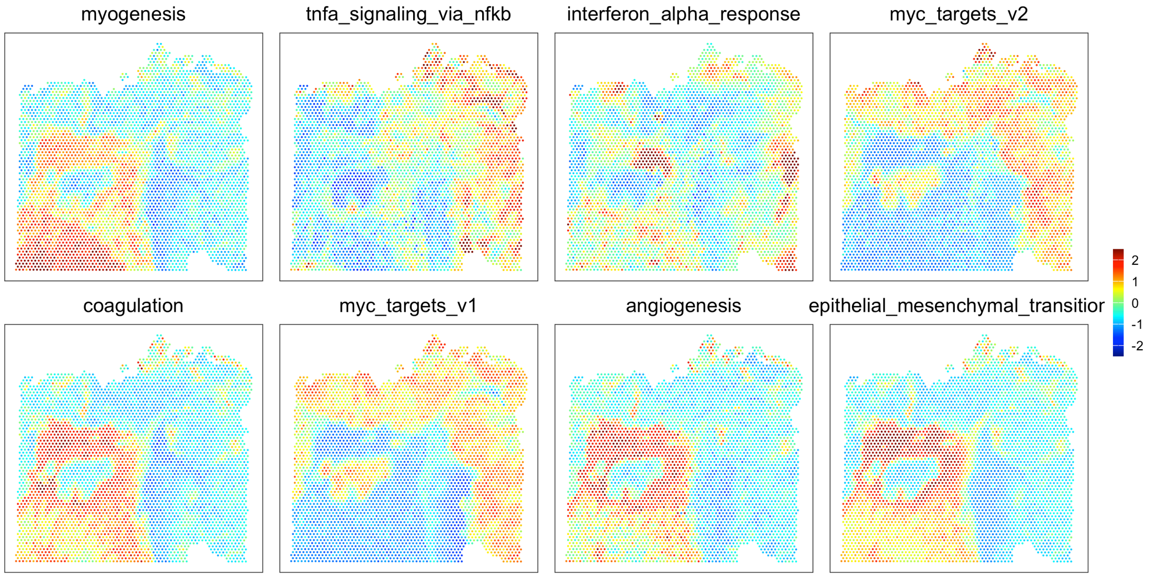

To ease interpretability, we can stratify `AUCell` scores by spot labels; these may stem from an unsupervised or deconvolution-based approach:

```r
for (. in c("Leiden", "RCTD")) {
    # aggregate AUC values by cluster
    mu <- aggregateAcrossCells(auc[top, ], spe[[.]],
        use.assay.type="AUC", statistics="mean")
    # visualize as (cluster x set) heatmap
    pheatmap(
        mat=t(assay(mu)), scale="column", col=pals::coolwarm(), main=.,
        cellwidth=10, cellheight=10, treeheight_row=5, treeheight_col=5)
}
```

For the latter, we may instead correlate set scores with proportion estimates (rather than discretizing labels according to the dominant subpopulation):

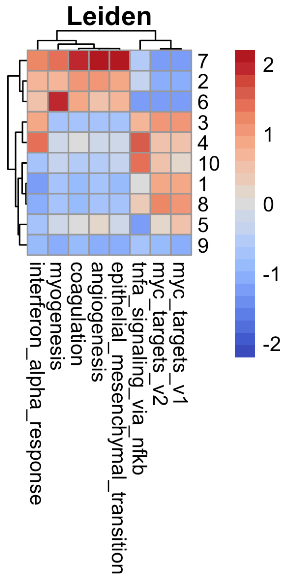

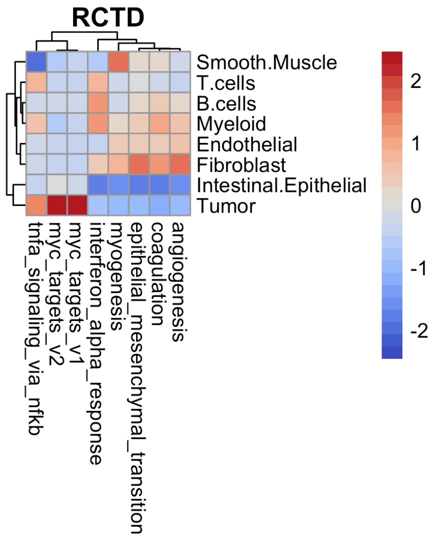


```r
# correlate 'AUCell' signature scores with subpopulation
# proportion estimates from deconvolution with 'RCTD'
cm <- cor(as.matrix(ws), t(assay(auc[top, ])))

pheatmap(cm,
    col=pals::coolwarm(),
    breaks=seq(-1, 1, length=25),
    cellwidth=10, cellheight=10,
    treeheight_row=5, treeheight_col=5)
```

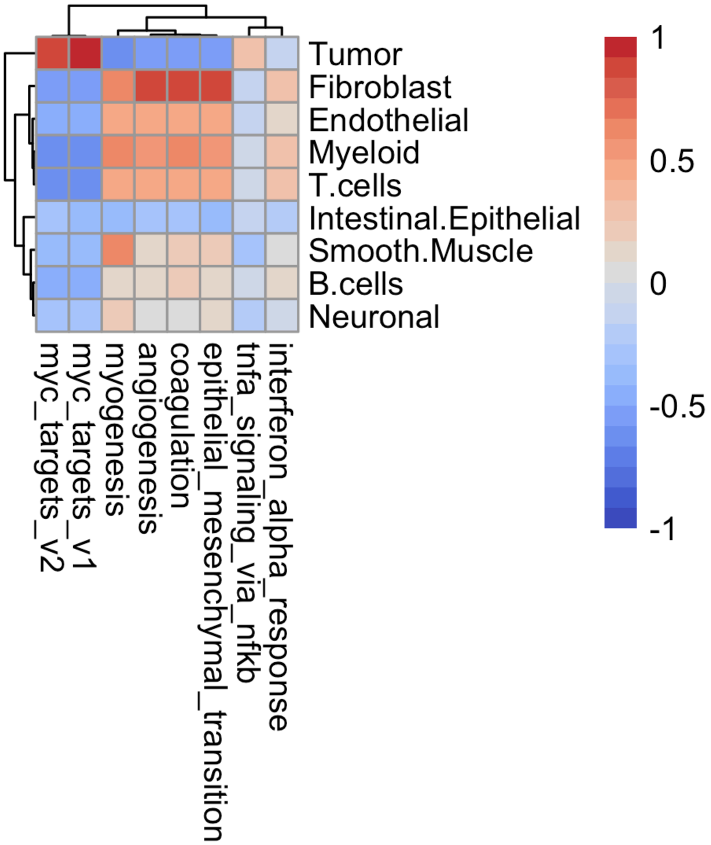

Appendix

References

Aibar, Sara, Carmen Bravo González-Blas, Thomas Moerman, Vân Anh Huynh-Thu, Hana Imrichova, Gert Hulselmans, Florian Rambow, et al. 2017. "SCENIC: Single-Cell Regulatory Network Inference and Clustering." *Nature Methods* 14: 1083–86. https://doi.org/10.1038/nmeth.4463.

Cable, Dylan M., Evan Murray, Luli S. Zou, Aleksandrina Goeva, Evan Z. Macosko, Fei Chen, and Rafael A. Irizarry. 2022. "Robust Decomposition of Cell Type Mixtures in Spatial Transcriptomics." *Nature Biotechnology* 40: 517–26. https://doi.org/10.1038/s41587-021-00830-w.

de Oliveira, Michelli Faria, Juan Pablo Romero, Meii Chung, Stephen R. Williams, Andrew D. Gottscho, Anushka Gupta, Susan E. Pilipauskas, et al. 2025. "High-Definition Spatial Transcriptomic Profiling of Immune Cell Populations in Colorectal Cancer." *Nature Genetics* 57: 1512–23. https://doi.org/10.1038/s41588-025-02193-3.

Workflow: Visium HD

Introduction

Visium HD is a next-generation spatial transcriptomics technology developed by 10x Genomics, designed to provide single-cell resolution spatial gene expression data across entire tissue sections. Commercially available since 2024, Visium HD advanced the platform's resolution from 55 µm spots to bins of subcellular resolution (2 µm). In this workflow, we will demonstrate common analysis steps with a Visium HD dataset on colorectal cancer (de Oliveira et al. 2025).

Dependencies

```
library(Banksy)
library(BiocParallel)
library(dplyr)
library(DropletUtils)
library(ggplot2)
library(ggspavis)
library(igraph)
library(magick)
library(OSTA.data)
library(patchwork)
library(pheatmap)
library(scater)
library(scran)
library(scuttle)
library(sf)
library(spacexr)
library(SpatialExperiment)
library(SpotSweeper)
library(Statial)
library(tidyr)
library(VisiumIO)
```

In this demo, we perform deconvolution on 16 µm bins and compare the concordance with results on 8 µm bins, provided by 10x Genomics.

```
# retrieve dataset from OSF repo
id <- "VisiumHD_HumanColon_Oliveira"
pa <- OSTA.data_load(id)
dir.create(td <- tempfile())
unzip(pa, exdir=td)
```

Here, we only demonstrate the analysis for patient 2 (P2). Other patients, such as P1 and P5, also have public Chromium, Visium HD, and Xenium data available for the CRC group (see Chapter 1). It would be interesting to investigate the differences in malignant cells/bins (e.g., DEGs

```
# read 8um bins into 'SpatialExperiment'
vhd8 <- TENxVisiumHD(
    spacerangerOut=td,
    processing="filtered",
    format="h5",
    images="lowres",
    bin_size="008") |>
    import()
# use gene symbols as feature names
gs <- rowData(vhd8)$Symbol
rownames(vhd8) <- make.unique(gs)
vhd8
```

```
## class: SpatialExperiment
## dim: 18085 545913
## metadata(2): resources spatialList
## assays(1): counts
## rownames(18085): SAMD11 NOC2L ... MT-ND6 MT-CYB
## rowData names(3): ID Symbol Type
## colnames(545913): s_008um_00301_00321-1 s_008um_00526_00291-1 ...
##   s_008um_00353_00477-1 s_008um_00595_00611-1
## colData names(6): barcode in_tissue ... bin_size sample_id
## reducedDimNames(0):
## mainExpName: Gene Expression
## altExpNames(0):
## spatialCoords names(2) : pxl_col_in_fullres pxl_row_in_fullres
## imgData names(4): sample_id image_id data scaleFactor
```

These data come with bin-level annotations from deconvolution estimates by RCTD (Cable et al. 2022), implemented in *spacexr*. Specifically, two sets of labels are available that correspond to the most and second most frequent of 38 cell types per bin, respectively:

```
# retrieve annotations
gz <- "binned_outputs/square_008um/deconvolution.csv.gz"
df <- read.csv(file.path(td, gz), row.names=2)
head(df <- df[complete.cases(df), -1])
```

```
##                              DeconClass   DeconLabel1          DeconLabel2
## s_008um_00000_00001-1           singlet     Pericytes          Endothelial
## s_008um_00000_00017-1           singlet Enteric Glial           Enterocyte
## s_008um_00000_00018-1 doublet_uncertain Enteric Glial             Tumor II
## s_008um_00000_00019-1           singlet           vSM                 Tuft
## s_008um_00000_00020-1           singlet           vSM CD8 Cytotoxic T cell
## s_008um_00000_00023-1            reject Smooth Muscle                  vSM
```

```
# keep only annotated bins
vhd8 <- vhd8[, rownames(df)]
names(df) <- c("DeconClass", "DeconLabel1", "DeconLabel2")
colData(vhd8)[names(df)] <- df

lab <- grep("Label", names(cd <- colData(vhd8)), value=TRUE)
pal <- hcl.colors(length(unique(unlist(cd[lab]))), "Spectral")
```

```r
lapply(lab, \(.) {
  plotCoords(vhd8, annotate=., point_size=0.2, point_shape = 15) + ggtitle(.)
}) |>
  wrap_plots(nrow=1, guides="collect") &
  scale_color_manual(NULL, values=pal) &
  theme(legend.key.size=unit(0, "lines"))
```

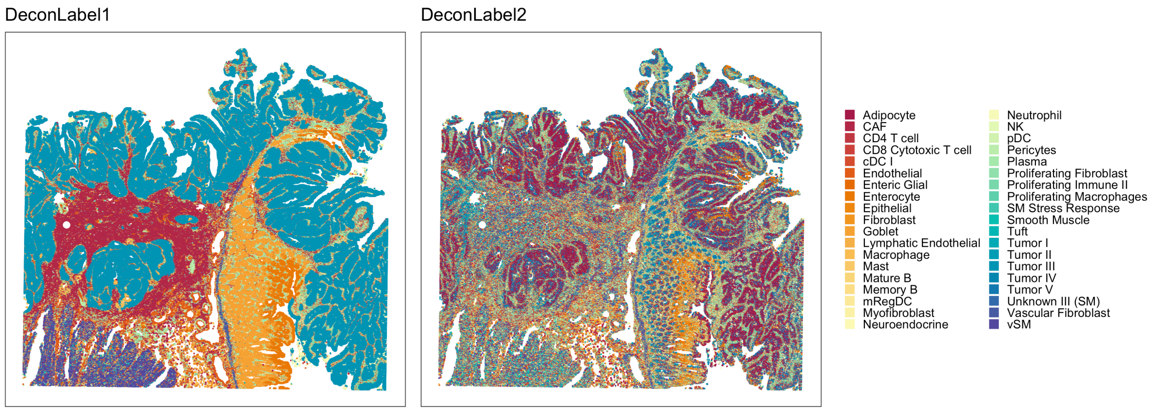

Another resolution of VisiumHD with 16 µm bin can be read in as follow:

```r
# read 16um bins into 'SpatialExperiment'
vhd16 <- TENxVisiumHD(
    spacerangerOut=td,
    processing="filtered",
    format="h5",
    images="lowres",
    bin_size="016") |>
    import()
# use symbols as feature names
gs <- rowData(vhd16)$Symbol
rownames(vhd16) <- make.unique(gs)
vhd16
```

```
## class: SpatialExperiment
## dim: 18085 137051
## metadata(2): resources spatialList
## assays(1): counts
## rownames(18085): SAMD11 NOC2L ... MT-ND6 MT-CYB
## rowData names(3): ID Symbol Type
## colnames(137051): s_016um_00052_00082-1 s_016um_00010_00367-1 ...
##   s_016um_00037_00193-1 s_016um_00144_00329-1
## colData names(6): barcode in_tissue ... bin_size sample_id
## reducedDimNames(0):
## mainExpName: Gene Expression
## altExpNames(0):
```

```
##  spatialCoords names(2) : pxl_col_in_fullres pxl_row_in_fullres
##  imgData names(4): sample_id image_id data scaleFactor
```

Remove bins overlaying empty tissue

Some global filtering based on library size is necessary to remove bins that overlay empty tissue.

```
vhd16$libsize <- colSums(counts(vhd16))
```

We can visualize such bins by zooming into an empty tissue region. On the left-hand side, one region shows a clear gap with low counts. Informed by H&E staining, bins located above tissue gaps should not be interpreted biologically, as the low counts in these bins are likely due to ambient RNA or transcript spillover. Therefore, they should be excluded from downstream analysis.
Based on a discussion with the original author at 10x Genomics, in this dataset, 8 µm bins with a library size below 100 are removed. Since we expect a 4-fold increase in library size across all bins at 16 µm, we can set the filtering threshold to 400.

```
p1 <- plotVisium(vhd16, annotate="libsize", point_shape = 22,
           zoom = TRUE, show_axes = TRUE, point_size = 1.3) +
    xlim(c(422, 442)) + ylim(c(318, 330)) + ggtitle("16 µm - original")

vhd16 <- vhd16[, vhd16$libsize > 400]

p2 <- plotVisium(vhd16, annotate="libsize", point_shape = 22,
           zoom = TRUE, show_axes = TRUE, point_size = 1.3) +
    xlim(c(422, 442)) + ylim(c(318, 330)) + ggtitle("16 µm - post > 400 UMI QC")

p1 | p2
```

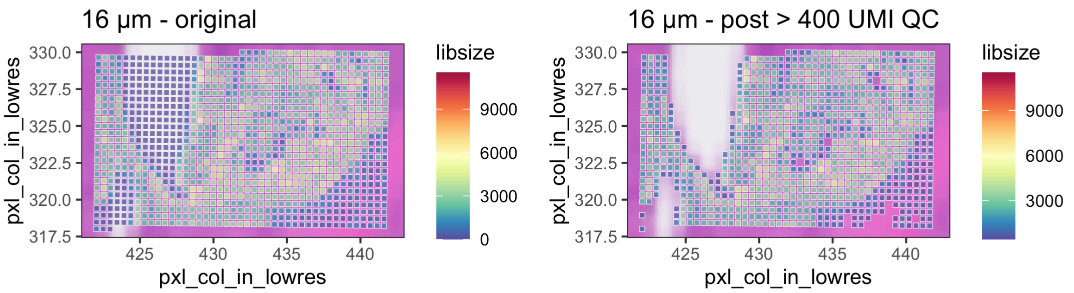

Clustering results generated by Space Ranger are provided for both resolutions.

```
csv <- list.files(td, "clustering.csv.gz", recursive=TRUE, full.names=TRUE)
dfs <- lapply(csv, read.csv, row.names="barcode")

colData(vhd8)$cluster <- factor(dfs[[1]][colnames(vhd8), "cluster"])
colData(vhd16)$cluster <- factor(dfs[[2]][colnames(vhd16), "cluster"])
```

We can visualize the given clustering results at 8 µm and 16 µm resolutions. Later, we will

compare the clustering result at 16 µm with that from Banksy.

```
(plotCoords(vhd8,
    annotate="cluster", point_size=0.05, point_shape=15,
    pal=unname(pals::kelly())) + ggtitle("8 µm")) |
(plotCoords(vhd16[, !is.na(vhd16$cluster)],
    annotate="cluster", point_size=0.1, point_shape=15,
    pal=unname(pals::kelly())) + ggtitle("16 µm"))
```

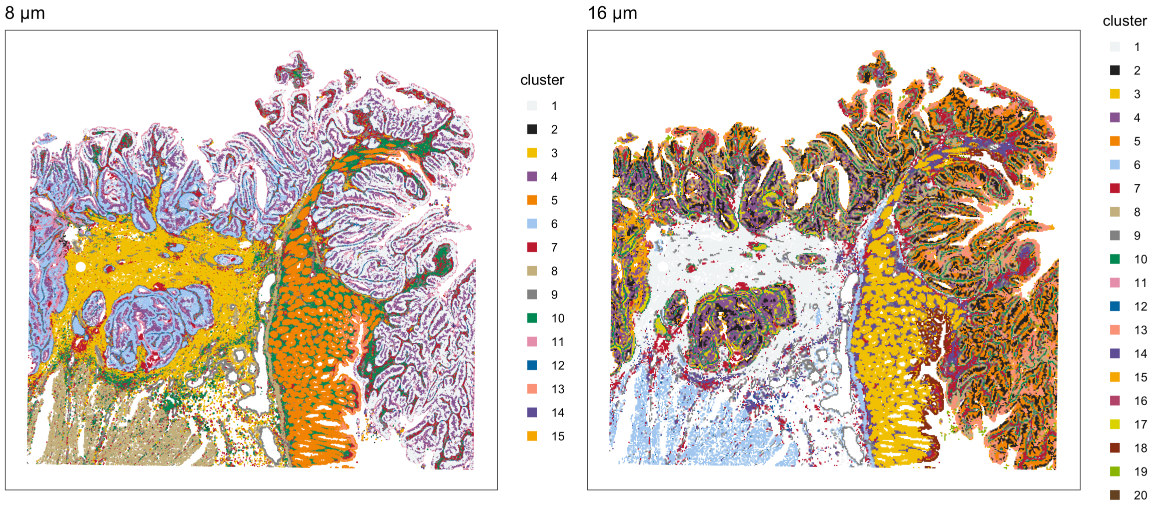

To keep runtimes low, our downstream analysis will be performed on a subset area of 16 µm bins (1/64 of the data coverage area), which we read in here; there are no annotations available for this resolution in the public domain.

Note that 8 and 16 µm binned Visium HD data share the same full-resolution H&E image and scaling factor.

```
.rng <- \(spe) {
    xy <- spatialCoords(spe)*scaleFactors(spe)
    xs <- range(xy[, 1]); ys <- nrow(imgRaster(spe))-range(xy[, 2])
    x1 <- xs[1] + 4*(xs[2]-xs[1])/8; x2 <- xs[2] - 3*(xs[2]-xs[1])/8
    y1 <- ys[1] + 3*(ys[2]-ys[1])/8; y2 <- ys[2] - 4*(ys[2]-ys[1])/8
    list(box=c(x1, x2, y1, y2), cov=c(xs, ys))
}
vhd8r <- .rng(vhd8)
# vhd16r <- .rng(vhd16)
# use 8um bounding boxes to subset spes at both resolutions;
# it is similar to 16um's box range
.box <- \(roi, lty, lwd, col) {
    geom_rect(
        xmin=vhd8r[[roi]][1], xmax=vhd8r[[roi]][2],
        ymin=vhd8r[[roi]][3], ymax=vhd8r[[roi]][4],
        col=col, fill=NA, linetype=lty, linewidth=lwd)
}
cov <- .box(roi="cov", lty=2, lwd=1/2, col="grey")
```

```r
box <- .box(roi="box", lty=4, lwd=2/3, col="black")
# plotting
.lim <- \(spe) list(
    xlim(spe[["box"]][c(1, 2)]),
    ylim(spe[["box"]][c(4, 3)]))

plotVisium(vhd8, spots=FALSE, point_shape=22) + cov + box +
    ggtitle("grey: data coverage\n black: zoomed region") +
plotVisium(vhd8, point_size=0.8, zoom=TRUE, point_shape=22) + .lim(vhd8r) +
    ggtitle("8 µm bins in black box") +
plotVisium(vhd16, point_size=1.6, zoom=TRUE, point_shape=22) + .lim(vhd8r) +
    ggtitle("16 µm bins in black box") +
plot_layout(nrow=1, widths=c(1.5, 1, 1)) & facet_null() &
    theme(plot.title=element_text(hjust=0.5, vjust=0.5))
```

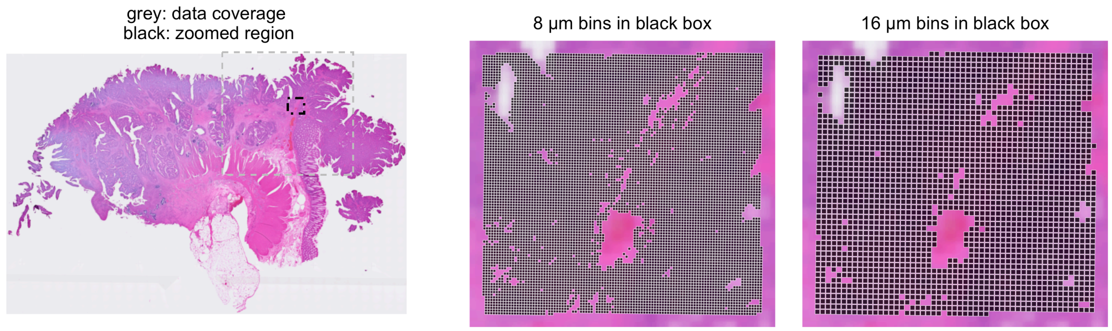

Subset the 16 µm object to bins in the black box:

```r
.sub <- function(spe, rng, roi="box") {
    xs <- rng[[roi]][c(1, 2)]
    ys <- nrow(imgRaster(spe)) - rng[[roi]][c(3,4)]
    xy <- spatialCoords(spe)*scaleFactors(spe)
    spe[, xy[, 1] > xs[1] & xy[, 1] < xs[2] &
          xy[, 2] > ys[1] & xy[, 2] < ys[2] ]
}
.vhd16 <- .sub(spe=vhd16, rng=vhd8r)
dim(.vhd16)
```

```
## [1] 18085  2416
```

From now on, we are operating on 2416 16 µm bins.

Quality control

As detailed in Chapter 2, we use *SpotSweeper* to perform quality control on the subsetted 16 µm data.

```r
# calculate per-cell QC metrics
mt <- grepl("^MT-", rownames(.vhd16))
.vhd16 <- addPerCellQCMetrics(.vhd16, subsets=list(mt=mt))
# determine outliers based on
# - low log-library size
# - few uniquely detected features
# - high mitochondrial count fraction
.vhd16 <- localOutliers(.vhd16, metric="sum", direction="lower", log=TRUE)
.vhd16 <- localOutliers(.vhd16, metric="detected", direction="lower", log=TRUE)
.vhd16 <- localOutliers(.vhd16, metric="subsets_mt_percent", direction="higher", log=TRUE)
.vhd16$discard <-
    .vhd16$sum_outliers |
    .vhd16$detected_outliers |
    .vhd16$subsets_mt_percent_outliers
# tabulate number of bins retained
# vs. removed by any criterion
table(.vhd16$discard)
```

```
##
##  FALSE  TRUE
##   2399    17
```

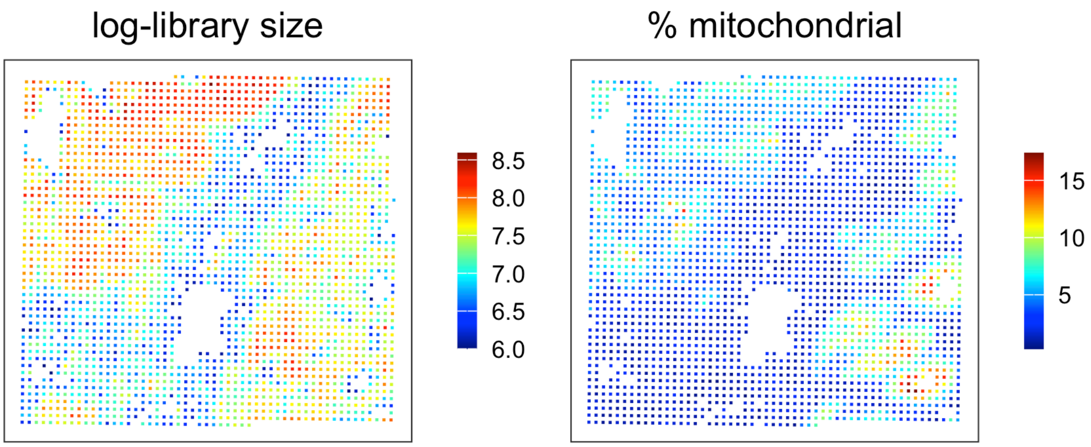

We can visualize the local outliers identified by `SpotSweeper` in space:

```r
plotCoords(.vhd16, point_shape=15, annotate="discard") + ggtitle("discard") +
plotCoords(.vhd16, point_shape=15, annotate="sum_outliers") + ggtitle("low_lib_size") +
plotCoords(.vhd16, point_shape=15, annotate="detected_outliers") + ggtitle("low_n_features") +
plot_layout(nrow=1, guides="collect") &
    theme(
        plot.title=element_text(hjust=0.5),
        legend.key.size=unit(0, "lines")) &
    guides(col=guide_legend(override.aes=list(size=3))) &
    scale_color_manual("discard", values=c("lavender", "purple"))
```

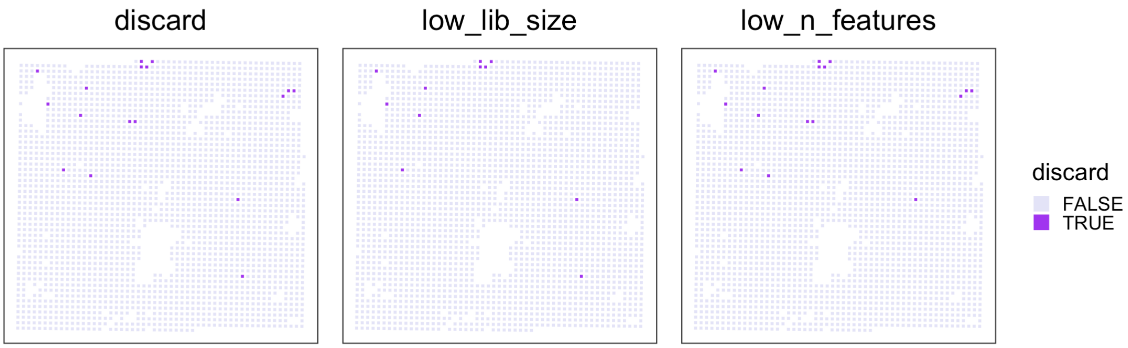

Lastly, we subset the Visium HD 16 µm object to bins that passed QC:

```
.vhd16 <- .vhd16[, !.vhd16$discard]
dim(.vhd16)
```

```
## [1] 18085  2399
```

Clustering

First, we identify highly variable genes (HVGs) in the target area:

Note that spatially variable genes (SVGs) could be used instead; see Chapter 3.

```
.vhd16 <- logNormCounts(.vhd16)
dec <- modelGeneVar(.vhd16)
hvg <- getTopHVGs(dec, n=3e3)
```

As detailed in Chapter 6, *Banksy* (Singhal et al. 2024) utilizes a pair of spatial kernels to capture gene expression variation, followed by dimension reduction and graph-based clustering to identify spatial domains.

```
# set seed for random number generation
# in order to make results reproducible
set.seed(112358)
# 'Banksy' parameter settings
k <- 8   # consider first order neighbors
l <- 0.2 # use little spatial information
a <- "logcounts"
xy <- c("array_row", "array_col")
# restrict to selected features
tmp <- .vhd16[hvg, ]
# compute spatially aware 'Banksy' PCs
tmp <- computeBanksy(tmp, assay_name=a, coord_names=xy, k_geom=k)
tmp <- runBanksyPCA(tmp, lambda=l, npcs=20)
reducedDim(.vhd16, "PCA") <- reducedDim(tmp)
## run UMAP (for visualization purposes only)
# .vhd16 <- runUMAP(.vhd16, dimred="PCA")
# build cellular shared nearest-neighbor (SNN) graph
g <- buildSNNGraph(.vhd16, use.dimred="PCA", type="jaccard", k=20)
```

```
# cluster using Leiden community detection algorithm
k <- cluster_leiden(g, objective_function="modularity", resolution=1.2)
table(.vhd16$Banksy <- factor(k$membership))
```

```
##
##    1   2   3   4   5   6   7   8   9  10  11  12  13
##  153 184 317 186 228 181 215 152 178 113 147 163 182
```

Next, we can perform differential gene expresison (DGE) analysis to identify markers for each cluster; c.f. Chapter 7. We also compute the cluster-wise mean expression of selected markers:

```
# differential gene expression analysis
mgs <- findMarkers(.vhd16, groups=.vhd16$Banksy, direction="up")
# select for a few markers per cluster
top <- lapply(mgs, \(df) rownames(df)[df$Top <= 2])
top <- unique(unlist(top))
# average expression by clusters
pbs <- aggregateAcrossCells(.vhd16,
    ids=.vhd16$Banksy, subset.row=top,
    use.assay.type="logcounts", statistics="mean")
```

The marker genes in each cluster can be visualized with a heatmap:

```
# visualize averages z-scaled across clusters
pheatmap(
    mat=t(assay(pbs)), scale="column", breaks=seq(-2, 2, length=101),
    cellwidth=10, cellheight=10, treeheight_row=5, treeheight_col=5)
```

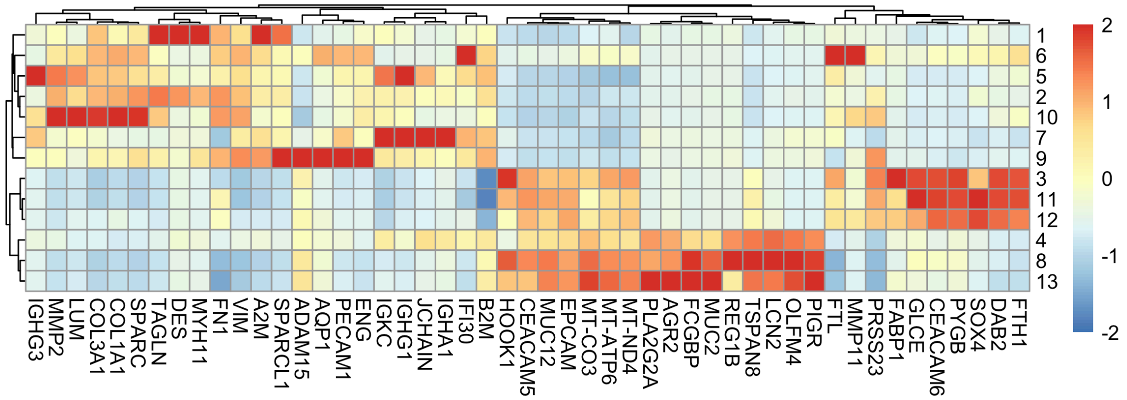

Or, we can visualize the bin-wise expression of selected markers in space:

```
gs <- c("MMP2", "PIGR", "IGHG1")
ps <- lapply(gs, \(.) plotCoords(.vhd16, annotate=., point_shape=15,
                                 point_size=0.8, assay_name="logcounts"))
wrap_plots(ps, nrow=1) & theme(
  legend.key.width=unit(0.5, "lines"),
  legend.key.height=unit(1, "lines")) &
  scale_color_gradientn(colors=rev(hcl.colors(9, "Rocket")))
```

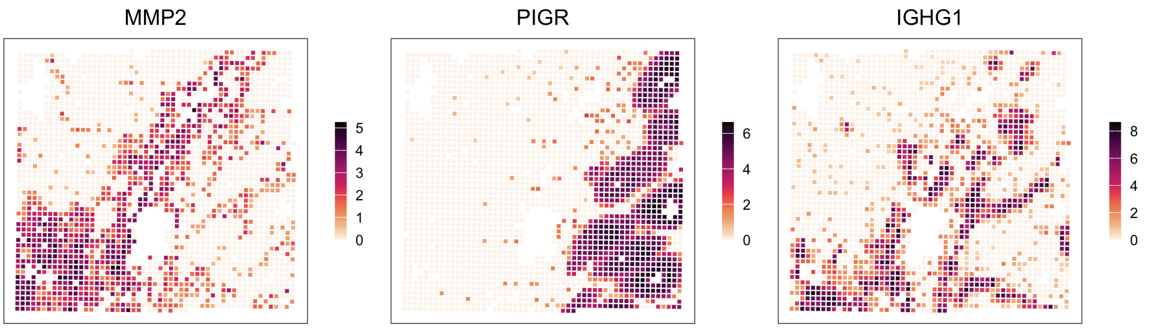

Deconvolution

Load single-cell reference

We will now perform deconvolution on the 16 µm binned Visium HD data. First, we retrieve matching (Chromium) scRNA-seq data, which includes low- (`Level1`) and high-resolution (`Level2`) annotations of cells into 9 respectively 31 clusters. In order to ensure similar transcriptional profile across technologies, we filter the reference population to patient 2 only.

```
# retrieve dataset from OSF repository
id <- "Chromium_HumanColon_Oliveira"
pa <- OSTA.data_load(id)
dir.create(td <- tempfile())
unzip(pa, exdir=td)
# read into `SingleCellExperiment`
fs <- list.files(td, full.names=TRUE)
h5 <- grep("h5$", fs, value=TRUE)
sce <- read10xCounts(h5, col.names=TRUE)
# add cell metadata
csv <- grep("csv$", fs, value=TRUE)
cd <- read.csv(csv, row.names=1)
colData(sce)[names(cd)] <- cd[colnames(sce), ]
# use gene symbols as feature names
gs <- rowData(sce)$Symbol
rownames(sce) <- make.unique(gs)
# exclude cells deemed to be of low-quality
sce <- sce[, sce$QCFilter == "Keep"]
# subset cells from same patient
sce <- sce[, grepl("P2", sce$Patient)]
sce
```

```
## class: SingleCellExperiment
## dim: 18082 67568
## metadata(1): Samples
## assays(1): counts
## rownames(18082): SAMD11 NOC2L ... MT-ND6 MT-CYB
## rowData names(3): ID Symbol Type
```

```
##   colnames(67568): AAACAAGCAACAGCTAACTTTAGG-1
##     AAACAAGCAACTGTTCACTTTAGG-1 ... TTTGTGAGTGCGTACCATGTTGAC-24
##     TTTGTGAGTGGAAGCTATGTTGAC-24
##   colData names(7): Sample Barcode ... Level1 Level2
##   reducedDimNames(0):
##   mainExpName: NULL
##   altExpNames(0):
```

Single-cell reference annotation mapping

We can visualize the mapping between low- and high-resolution annotations in the single-cell reference data with a contingency table:

```
fq <- prop.table(table(sce$Level1, sce$Level2), 2)
pheatmap(fq, cellwidth=10, cellheight=10, treeheight_row=5, treeheight_col=5)
```

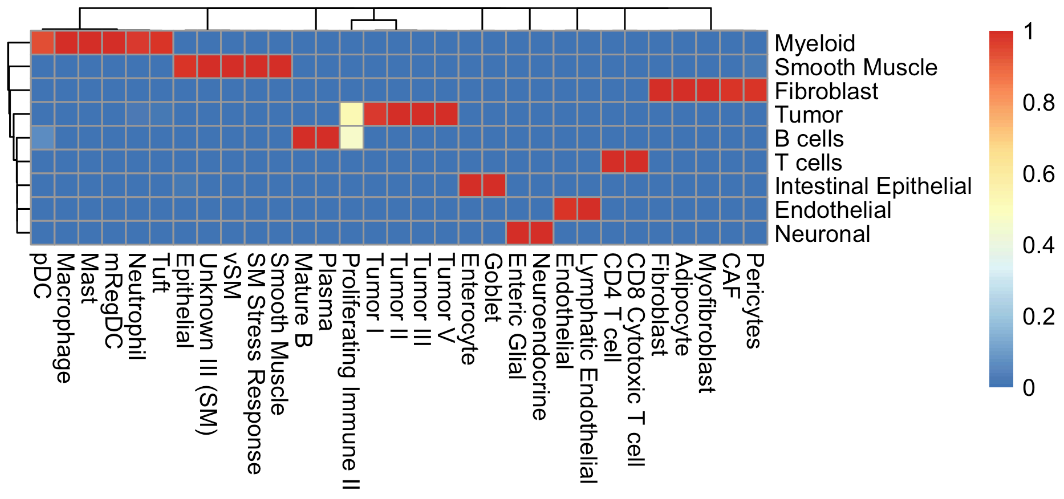

Note that "**Proliferating Immune II**" spans evenly between "**B cells**" and "**Tumor**", so we will make it a separate class, "**Prolif. Immune**" in the low-resolution annotations:

```
sce$Level1 <- ifelse(grepl("Prolif", sce$Level2), "Prolif. Immune", sce$Level1)
table(sce$Level1) # tabulate low-res. labels
```

```
##
##                B cells           Endothelial            Fibroblast
##                   6233                  1969                  7355
##  Intestinal Epithelial               Myeloid              Neuronal
##                   5949                  6353                  1216
##         Prolif. Immune        Smooth Muscle               T cells
##                    810                 16428                  6658
##                  Tumor
##                  14597
```

There are some differences in the provided deconvolution labels at 8 µm resolution and the high-resolution single-cell reference labels:

```r
# all single-cell reference labels
# are present in the Visium HD data
setdiff(sce$Level2, vhd8$DeconLabel1)
```

```
##  character(0)
```

```r
setdiff(sce$Level2, vhd8$DeconLabel2)
```

```
##  character(0)
```

```r
# but not vice versa
setdiff(vhd8$DeconLabel1, sce$Level2)
```

```
##  [1] "Proliferating Macrophages" "Proliferating Fibroblast"
##  [3] "Memory B"                  "Vascular Fibroblast"
##  [5] "cDC I"                     "NK"
##  [7] "Tumor IV"
```

```r
setdiff(vhd8$DeconLabel2, sce$Level2)
```

```
##  [1] "Proliferating Fibroblast"  "Memory B"
##  [3] "cDC I"                     "Proliferating Macrophages"
##  [5] "Vascular Fibroblast"       "Tumor IV"
##  [7] "NK"
```

Below, these extra classes in the Visium HD 8 m bin annotations will be ignored.

Simplify 8 µm annotations

We can merge the provided deconvolution annotation "DeconLabel1" (most likely cell type for singlets, most dominant type for doublets) into a lower resolution using the single-cell reference annotations:

```r
i <- match(vhd8$DeconLabel1, sce$Level2)
j <- match(vhd8$DeconLabel2, sce$Level2)
vhd8$.DeconLabel1 <- sce$Level1[i]
vhd8$.DeconLabel2 <- sce$Level1[j]
vhd8 <- vhd8[, !is.na(vhd8$.DeconLabel1)]
table(vhd8$.DeconLabel1)
```

```
##
##                 B cells            Endothelial             Fibroblast
##                   12279                  19978                  75080
##  Intestinal Epithelial                Myeloid               Neuronal
##                   40683                  20811                   1200
##         Prolif. Immune          Smooth Muscle                T cells
##                    3660                  15913                   6158
##                  Tumor
##                  215865
```

According to `RCTD`, approximately 75% of the 8 µm bins are singlets:

```
round(100*mean(vhd8$DeconClass == "singlet"), 2)
```

```
## [1] 77.39
```

Let us check the top-5 common doublet pairs according to the `"doublet_certain"` class:

```
dbl <- vhd8$DeconClass == "doublet_certain"
lab <- c(".DeconLabel1", ".DeconLabel2")
df <- data.frame(colData(vhd8)[dbl, lab])
# sort as to ignore order
df <- apply(df, 1, sort)
df <- do.call(rbind, df)
names(df) <- lab
# count unique pairs
ij <- paste(df[, 1], df[, 2], sep=";")
head(sort(table(ij), decreasing=TRUE), 5)
```

```
## ij
##      Myeloid;Tumor B cells;Fibroblast Fibroblast;Myeloid        Tumor;Tumor
##               3799               3670               3453               2742
##   Fibroblast;Tumor
##               2532
```

We can see that myeloid are commonly co-localized with tumor and fibroblasts cells; most doublets are homotypic, i.e., they are (mostly) composed of one low-resolution type.

ℹ Subset 8um bins within black bounding box

We can perform the same subsetting procedure to 8 µm bins and compare the difference in the number of bins in the zoomed (black box) area.

```
# subset zoomed (black box) area
.vhd8 <- .sub(spe=vhd8, rng=vhd8r)
# compare number of bins between resolutions
(n <- ncol(.vhd8))
```

```
## [1] 8933
```

```
(m <- ncol(.vhd16))
```

```
## [1] 2399
```

```
round(n/m, 2)
```

```
## [1] 3.72
```

As expected, the number of 8 µm bins is about 4 times the number of 16 µm bins. We will visualize the same subset for both bin sizes in any downstream analyses.

Run RCTD on 16 µm bins

We also assume there are at most two cell types in a 16 µm bin as in 8 µm, and thus we would expect a smaller proportion of singlets.

```
# downsample to at most 4,000 cells per cluster for 'sce'
# (this is done only to keep runtime/memory low)
cs <- split(seq_len(ncol(sce)), sce$Level1)
cs <- lapply(cs, \(.) sample(., min(length(.), 4e3)))
ncol(.sce <- sce[, unlist(cs)])
rctd_data <- createRctd(.vhd16, .sce, cell_type_col="Level1")
(res <- runRctd(rctd_data, max_cores=4, rctd_mode="doublet"))
```

Weights inferred by RCTD correspond to proportions of cell types, such that they sum to 1 for a given observation (expect for rejected ones, which sum to 0):

```
# counts rejected observations
ws <- assay(res, "weights")
table(colSums(ws) == 0)
```

```
##
##  FALSE  TRUE
##   2388    11
```

```
# add proportion estimates as metadata
ws <- data.frame(t(as.matrix(ws)))
colData(.vhd16)[names(ws)] <- ws[colnames(.vhd16), ]
```

Next, we can spatially visualize the deconvolution proportions estimates:

```
lapply(names(ws), \(.)
    plotCoords(.vhd16, annotate=., point_size=0.3, point_shape=15)) |>
    wrap_plots(nrow=2, guides="collect") & theme(
    legend.key.width=unit(0.5, "lines"),
    legend.key.height=unit(1, "lines")) &
    scale_color_gradientn(colors=rev(hcl.colors(9, "Rocket")))
```

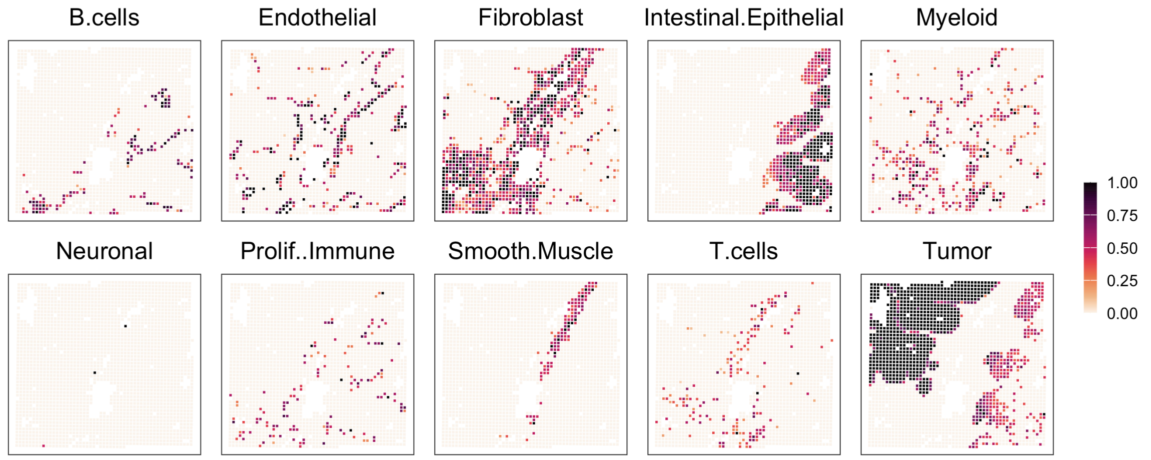

We may also obtain the majority vote class for 16 µm bins:

This type of analysis procedure is underlying the annotations provided by the authors.

```
# derive majority vote labels
ids <- names(ws)[apply(ws, 1, which.max)]
ids <- gsub("\\.([A-z])", " \\1", ids)
idx <- match(colnames(.vhd16), rownames(ws))
table(.vhd16$.DeconLabel1 <- factor(ids[idx]))
```

```
##
##                 B cells          Endothelial           Fibroblast
##                     121                  223                  506
##   Intestinal Epithelial              Myeloid             Neuronal
##                     368                  180                    2
##          Prolif. Immune        Smooth Muscle              T cells
##                      77                   60                   49
##                   Tumor
##                     813
```

Majority voted cell type frequency difference between 8 and 16 µm bins

We can also check the proportional frequency difference between 8 and 16 µm bins in our region of interest:

```
n8 <- table(.vhd8$.DeconLabel1)
n16 <- table(.vhd16$.DeconLabel1)
df <- data.frame(x=as.numeric(n8/n16))
ggplot(df, aes(x)) + geom_density() +
    xlab("Cell type frequency ratio") +
    ggtitle("distribution of cell type frequency\nratio between 8 vs. 16 µm bins") +
    scale_x_continuous(breaks=seq(0, max(df$x), by=2)) +
    theme_classic() + theme(plot.title=element_text(hjust=0.5))
```

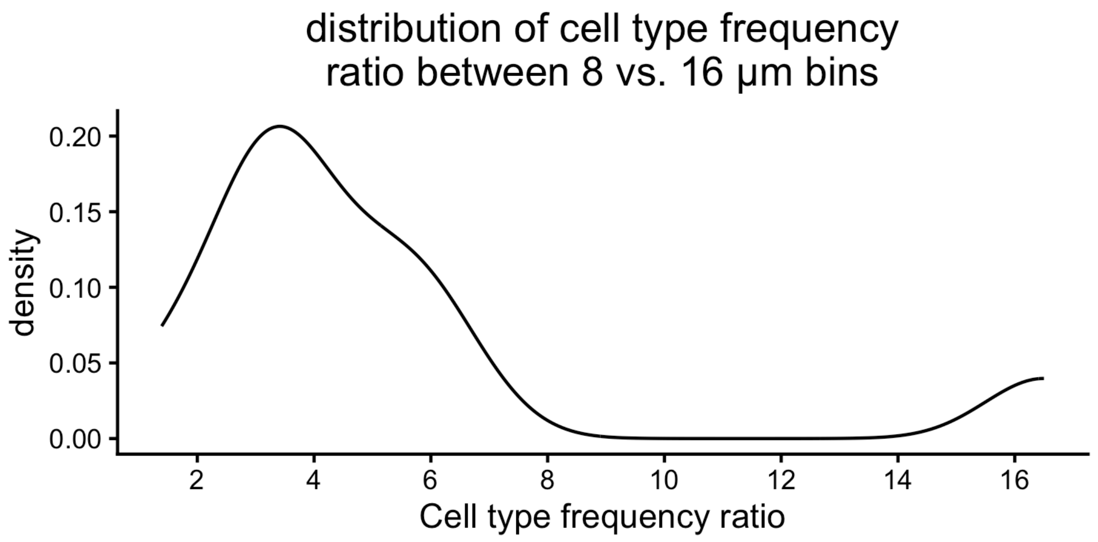

Most cell types appear around 3-4 times more in 8 compared to 16 µm bins, which is expected given the difference in bin size.

Deconvolution results at different resolutions

Let's visualize deconvolution result for 8 and 16 µm bins in the zoomed area:

```
plotVisium(.vhd8,
    annotate=".DeconLabel1", zoom=TRUE,
    point_size=0.8, point_shape=22) +
    ggtitle("8 µm") +
plot_spacer() +
plotVisium(.vhd16,
    annotate=".DeconLabel1", zoom=TRUE,
    point_size=1.6, point_shape=22) +
    ggtitle("16 µm") +
plot_layout(nrow=1, guides="collect", widths=c(1, 0.05, 1)) &
    guides(col=guide_legend(override.aes=list(size=2))) &
    scale_fill_manual(values=unname(pals::trubetskoy())) &
    facet_null() & theme(
        plot.title=element_text(hjust=0.5),
        legend.key.size=unit(0, "lines"))
```

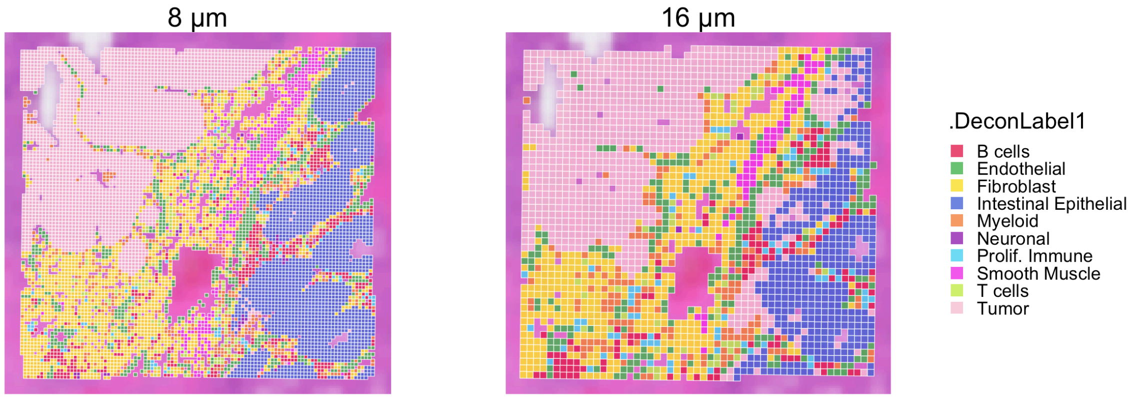

We observe clear agreement in deconvolution at both 8 µm and 16 µm resolutions; however, the 8 µm resolution better captures fine structures, such as the string-like shape of endothelial and fibroblast bins extending into the upper left tumor region.

Concordance of results

We can visualize the results spatially:

```
plotVisium(.vhd16,
    annotate="cluster", zoom=TRUE,
    point_shape=22, point_size=1.6,
    pal=unname(pals::kelly())) +
plot_spacer() +
plotVisium(.vhd16,
    annotate="Banksy", zoom=TRUE,
```

```
    point_shape=22, point_size=1.6,
    pal=unname(pals::kelly())) +
plot_spacer() +
plotVisium(.vhd16,
    annotate=".DeconLabel1", zoom=TRUE,
    point_shape=22, point_size=1.6,
    pal=unname(pals::trubetskoy())) +
plot_layout(nrow=1, widths=c(1, 0.05, 1, 0.05, 1)) &
    facet_null() & theme(legend.key.size=unit(0, "lines"))
```

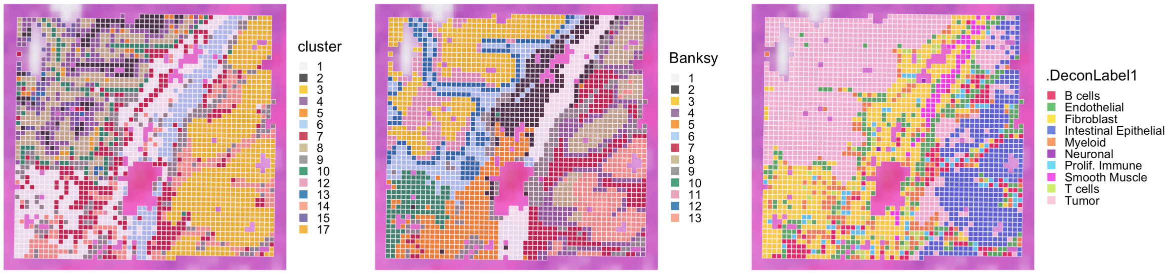

We observe that Banksy delineates tissue borders very well, while deconvolution provides direct biological insight into the underlying clusters. Their concordance can be visualized using a heatmap:

```
fq <- prop.table(table(.vhd16$Banksy, .vhd16$.DeconLabel1), 1)
pheatmap(fq, cellwidth=10, cellheight=10, treeheight_row=5, treeheight_col=5)
```

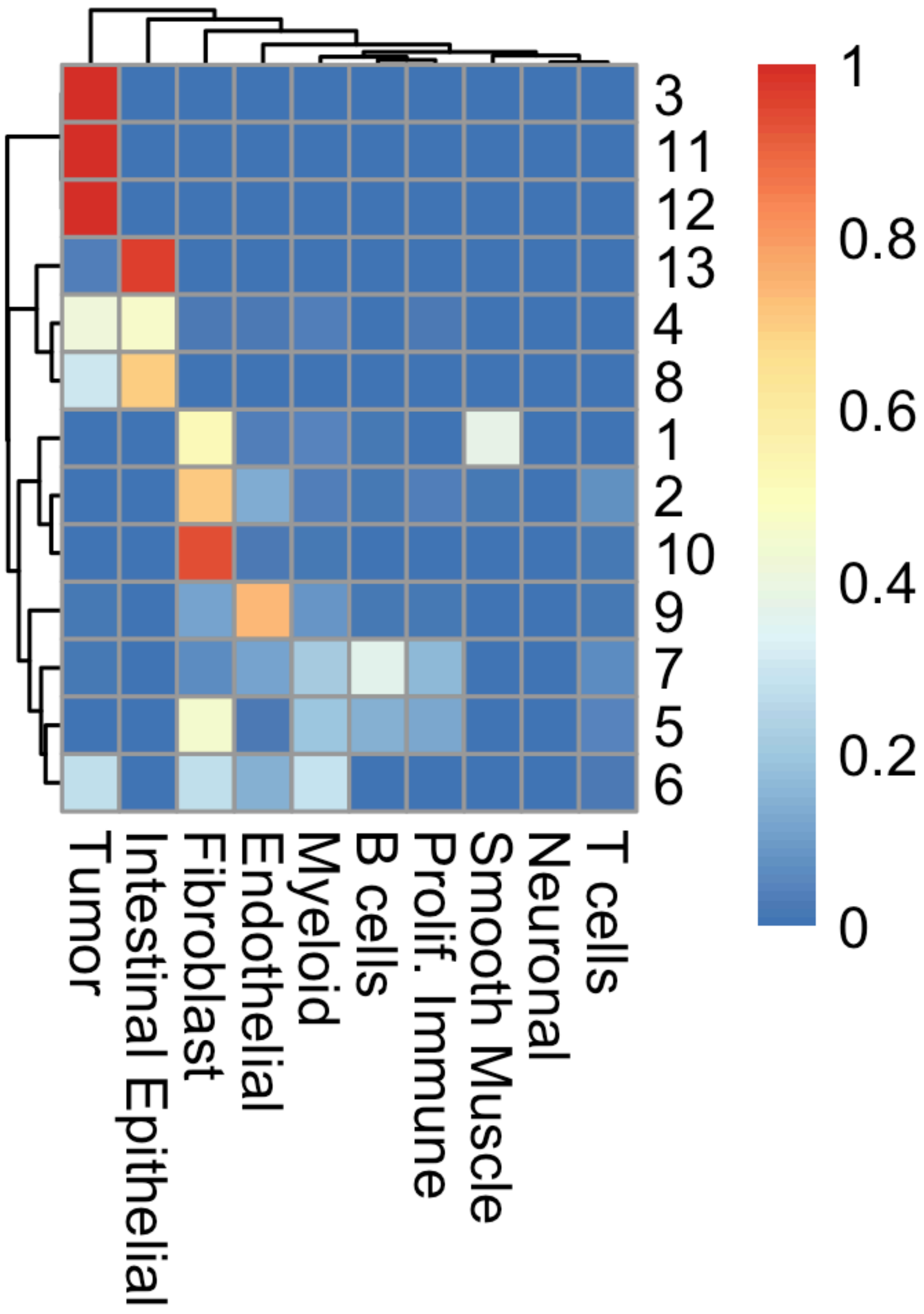

From the above heatmap, cluster 5 and 7 are mostly immune cells; cluster 9 maps to endothelial; cluster 1 and 13 correspond to intestinal epithelial and smooth muscle cells, respectively; fibroblasts map to cluster 10; tumor spans clusters 3, 11 and 12.

Neighborhood analysis

Here, we want to quantify the abundance between *T cells*, *B cells*, or *Myeloid* bins to *Fibroblast* or *Tumor* bins in 8 µm zoomed region. We use `getAbundances()` from *Statial* (Ameen et al. 2024) to calculate, for each bin, the abundance of other cell types within a radius of 200 px; the resulting (bins × types) matrix is stored as `reducedDim` slot `"abundances"`.

```
.vhd8 <- .vhd8[, !is.na(.vhd8$.DeconLabel1)]
xy <- data.frame(spatialCoords(.vhd8))
colData(.vhd8)[names(xy)] <- xy
.vhd8 <- getAbundances(.vhd8,
    spatialCoords=names(xy),
    cellType=".DeconLabel1",
    imageID="sample_id",
    r=200, nCores=4)
```

Let's have a look at the abundance and distance results, also including deconvolution results:

```
df <- reducedDim(.vhd8, "abundances")
df$CT <- .vhd8$.DeconLabel1
df[1:5, 1:5]
```

```
##                          Fibroblast T cells B cells Prolif. Immune Myeloid
##   s_008um_00388_00417-1          38       0       3              0       1
##   s_008um_00388_00418-1          43       0       3              1       0
##   s_008um_00388_00419-1          44       0       4              1       1
##   s_008um_00388_00420-1          47       0       8              1       1
##   s_008um_00388_00421-1          50       0      11              1       1
```

Now, we filter to only *T cells*, *B cells* and *Myeloid* bins, and investigate – within their neighborhood – the spatial proximity differences to *Fibroblast* or *Tumor* bins in terms of abundance.

```
source <- df$CT %in% c("T cells", "B cells", "Myeloid")
target <- c("Tumor", "Fibroblast", "CT")
fd <- pivot_longer(
    df[source, target], cols=-CT,
    names_to="target", values_to="n")
head(fd)
```

```
##   # A tibble: 6 x 3
##     CT      target         n
##     <chr>   <chr>      <int>
##   1 Myeloid Tumor          0
##   2 Myeloid Fibroblast    43
##   3 B cells Tumor          0
##   4 B cells Fibroblast    50
##   5 B cells Tumor          0
##   6 B cells Fibroblast    50
```

In this region, we observe more fibroblast than tumor bins around each of the immune bins. However, note that in this zoomed region, we have nearly 1.4 times more fibroblast bins compared to tumor bins. Thus, we need to scale abundance values by the number of target cell type bins observed in the region.

```
n_tum <- table(.vhd8$.DeconLabel1)["Tumor"]
n_fib <- table(.vhd8$.DeconLabel1)["Fibroblast"]
fd$p <- ifelse(fd$target == "Tumor", fd$n/n_tum, fd$n/n_fib)
ggplot(fd, aes(x=CT, y=n, fill=target)) +
    labs(y="# cells within 200px") +
ggplot(fd, aes(x=CT, y=p, fill=target)) +
    labs(y="relative abundance") +
plot_layout(nrow=1, guides="collect") &
    geom_boxplot(key_glyph="point") &
    scale_fill_manual(values=c("yellow", "pink")) &
    guides(fill=guide_legend(override.aes=list(shape=21, size=2))) &
    theme_classic() & theme(
        axis.title.x=element_blank(),
        legend.key.size=unit(0, "lines"))
```

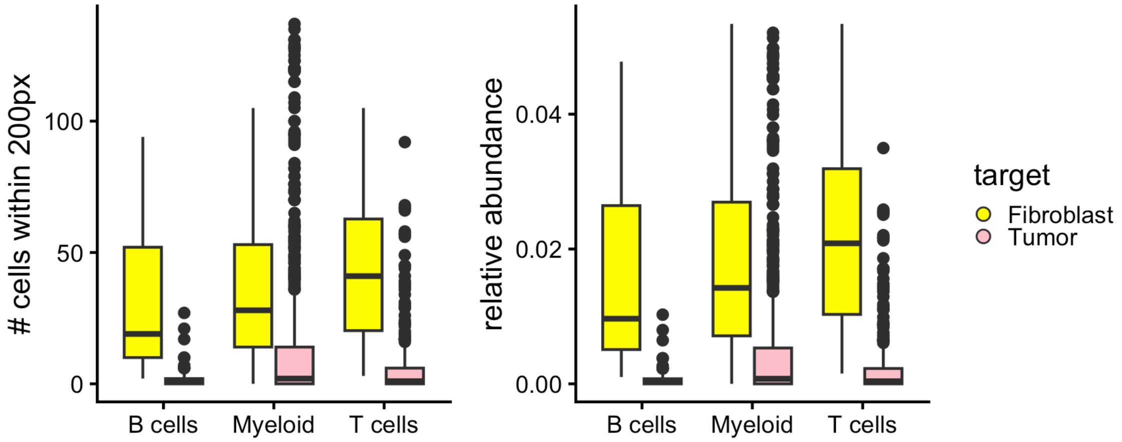

After correction, we see amplified differences in the relative abundance of fibroblast bins compared to tumor bins around each immune cell type in the zoomed region.

We can also investigate the "cell-cell-marker" relationship based on the minimum distance between two cell types within a given radius. The matrix of minimum distance between all pairwise cell types is stored in the reduced dimension, similar to how the abundance matrix was previously stored.

```
# NOTE: conversion to SCE is a temporary fix for a 'Statial' bug
# reported here: https://github.com/SydneyBioX/Statial/issues/16
tmp <- as(.vhd8, "SingleCellExperiment")
tmp <- getDistances(tmp,
    cellType=".DeconLabel1", imageID="sample_id",
    spatialCoords=names(xy), maxDist=200, nCores=4)
reducedDim(.vhd8, "distances") <- reducedDim(tmp, "distances")
```

Here, we aim to determine whether the expression level of the tumor marker changes in proximity to fibroblast. Using FTH1 as an example, we observe a decrease in FTH1 expression in tumor bins as the distance to fibroblast increase. This trend could be visualized using a scatter plot.

```
p <- plotStateChanges(
    cells=.vhd8,
    image="sample01",
    from="Tumor",
    to="Fibroblast",
    marker="FTH1",
    cellType=".DeconLabel1",
    imageID="sample_id",
    spatialCoords=c("array_col", "array_row"))
p$image + facet_null() | p$scatter
```

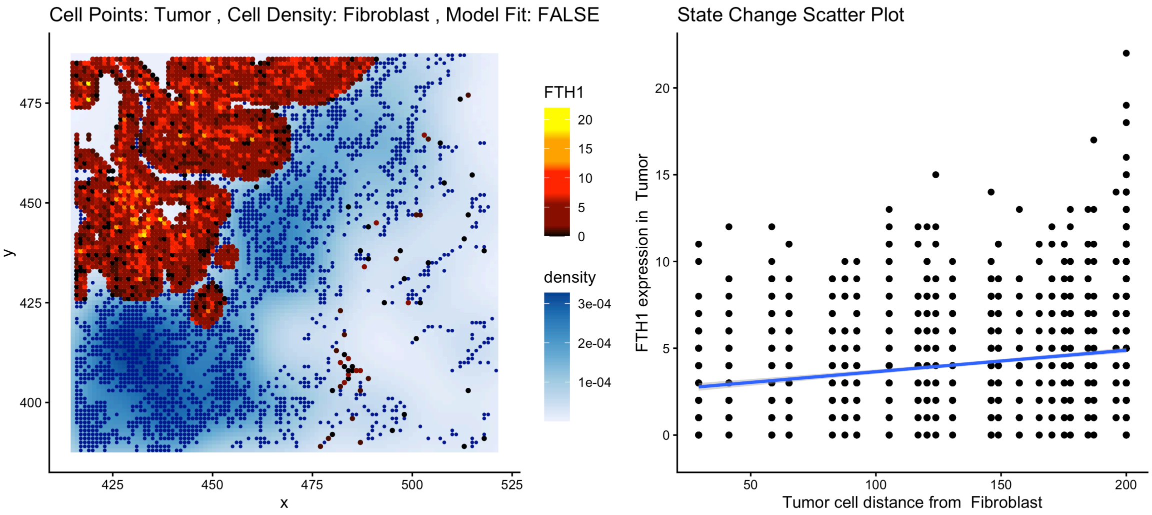

The same analysis steps could be applied to the (unfiltered) 16 m resolution data with the corresponding deconvolution results.

Conclusion

In this workflow, we demonstrated how to perform a standard analysis pipeline on a subset of Visium HD data binned at 16 µm. Most methods and visualization techniques developed for Visium can be applied to Visium HD. However, more sparsity is expected, as each data point (e.g. 2, 8, or 16 µm) has smaller area compared to Visium (55 µm). Each bin cannot be interpreted as a cell, but rather, a segmentation-free approach is applied here. Below are some resources for Visium HD to consider and incorporate into your pipeline:

Please see the following section for some cell-level Visium HD analysis guide.

- GitHub repository of `bin2cell` for H&E segmentation and obtaining cell boundaries.

- Analysis pipeline by 10x Genomics (April 2024). The analysis consists of three steps: run `StarDist` nuclei segmentation in Python, sum gene expression into nuclei based on segmentation mask, and downstream analysis with `scanpy` and `anndata`.
- Pipeline on cell typing for Visium HD in Python by Sanofi.

Cell-level Visium HD analysis

As of June, 2025, Visium HD enables direct output of H&E-segmented cell-level data, in addition to the previous `binned_outputs` at 2, 8, and 16 µm, from SpaceRanger v4. This update simplifies the additional cell segmentation required when using `bin2cell` or `StarDist`. However, a comparative study of segmentation methods on Visium HD data has yet to be conducted.

The morphology-driven, nucleus-based segmentation produces polygons representing nuclei and cells across the entire tissue, as illustrated in the schematics provided by 10x Genomics.

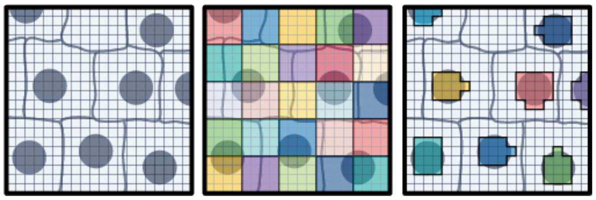

Figure 3.1: Two approaches for binning the 2x2 µm barcode squares in Visium HD data

Compared to binned analysis, Visium HD with cell-level segmentation is more similar to Xenium data, but with full-transcriptome coverage. Most analytical methods are transferable; however, at single-cell resolution, deconvolving each data point into multiple cell types should be adapted using label transfer or unsupervised clustering to assign single-cell type labels. For example, after clustering, we identified three differentially expressed genes:

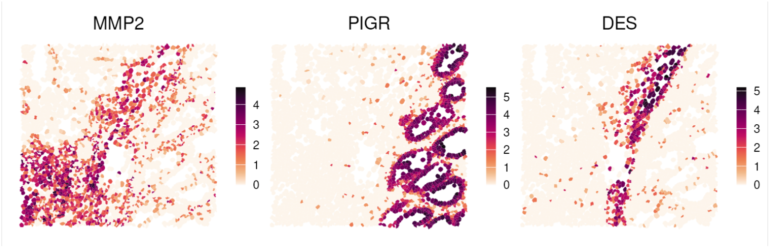

Figure 3.2: Differential marker gene expressions spatially visualized as polygons

For detailed cell-level Visium HD workflow, please refer to the EuroBioC2025 OSTA workshop.

Appendix

References

Aibar, Sara, Carmen Bravo González-Blas, Thomas Moerman, Vân Anh Huynh-Thu, Hana Imrichova, Gert Hulselmans, Florian Rambow, et al. 2017. "SCENIC: Single-Cell Regu-

latory Network Inference and Clustering." *Nature Methods* 14: 1083–86. https://doi.org/10.1038/nmeth.4463.

Ameen, Farhan, Nick Robertson, David M. Lin, Shila Ghazanfar, and Ellis Patrick. 2024. "Kontextual: Reframing Analysis of Spatial Omics Data Reveals Consistent Cell Relationships Across Images." *bioRxiv*. https://doi.org/10.1101/2024.09.03.611109.

Atta, Lyla, Kalen Clifton, Manjari Anant, Gohta Aihara, and Jean Fan. 2024. "Gene Count Normalization in Single-Cell Imaging-Based Spatially Resolved Transcriptomics." *Genome Biology* 25 (153). https://doi.org/10.1186/s13059-024-03303-w.

Bhuva, Dharmesh D., Chin Wee Tan, Agus Salim, Claire Marceaux, Marie A. Pickering, Jinjin Chen, Malvika Kharbanda, et al. 2024. "Library Size Confounds Biology in Spatial Transcriptomics Data." *Genome Biology* 25 (99). https://doi.org/10.1186/s13059-024-03241-7.

Cable, Dylan M., Evan Murray, Luli S. Zou, Aleksandrina Goeva, Evan Z. Macosko, Fei Chen, and Rafael A. Irizarry. 2022. "Robust Decomposition of Cell Type Mixtures in Spatial Transcriptomics." *Nature Biotechnology* 40: 517–26. https://doi.org/10.1038/s41587-021-00830-w.

de Oliveira, Michelli Faria, Juan Pablo Romero, Meii Chung, Stephen R. Williams, Andrew D. Gottscho, Anushka Gupta, Susan E. Pilipauskas, et al. 2025. "High-Definition Spatial Transcriptomic Profiling of Immune Cell Populations in Colorectal Cancer." *Nature Genetics* 57: 1512–23. https://doi.org/10.1038/s41588-025-02193-3.

Dong, Kangning, and Shihua Zhang. 2022. "Deciphering Spatial Domains from Spatially Resolved Transcriptomics with an Adaptive Graph Attention Auto-Encoder." *Nature Communications* 13 (1739). https://doi.org/10.1038/s41467-022-29439-6.

Hu, Yunfei, Manfei Xie, Yikang Li, Mingxing Rao, Wenjun Shen, Can Luo, Haoran Qin, Jihoon Baek, and Xin Maizie Zhou. 2024. "Benchmarking Clustering, Alignment, and Integration Methods for Spatial Transcriptomics." *Genome Biology* 25 (212). https://doi.org/10.1186/s13059-024-03361-0.

Janesick, Amanda, Robert Shelansky, Andrew D. Gottscho, Florian Wagner, Stephen R. Williams, Morgane Rouault, Ghezal Beliakoff, et al. 2023. "High Resolution Mapping of the Tumor Microenvironment Using Integrated Single-Cell, Spatial and in Situ Analysis." *Nature Communications* 14 (8353). https://doi.org/10.1038/s41467-023-43458-x.

Liang, Yuchen, Guowei Shi, Runlin Cai, Yuchen Yuan, Ziying Xie, Long Yu, Yingjian Huang, et al. 2024. "PROST: Quantitative Identification of Spatially Variable Genes and Domain Detection in Spatial Transcriptomics." *Nature Communications* 15 (600). https://doi.org/10.1038/s41467-024-44835-w.

Liu, Teng, Zhao-Yu Fang, Zongbo Zhang, Yongxiang Yu, Min Li, and Ming-Zhu Yin. 2024. "A Comprehensive Overview of Graph Neural Network-Based Approaches to Clustering for Spatial Transcriptomics." *Computational and Structural Biotechnology Journal* 23: 106–28. https://doi.org/10.1016/j.csbj.2023.11.055.

Liu, Wei, Xu Liao, Ziye Luo, Yi Yang, Mai Chan Lau, Yuling Jiao, Xingjie Shi, et al. 2023. "Probabilistic Embedding, Clustering, and Alignment for Integrating Spatial Transcriptomics Data with PRECAST." *Nature Communications* 14 (296). https://doi.org/10.1038/s41467-023-35947-w.

Lun, Aaron T. L., Davis J. McCarthy, and John C. Marioni. 2016. "A Step-by-Step Workflow for Low-Level Analysis of Single-Cell RNA-Seq Data with Bioconductor." *F1000Research* 5 (2122). https://doi.org/10.12688/f1000research.9501.2.

Maynard, Kristen R., Leonardo Collado-Torres, Lukas M. Weber, Cedric Uytingco, Brianna K. Barry, Stephen R. Williams, Joseph L. Catallini II, et al. 2021. "Transcriptome-Scale Spatial Gene Expression in the Human Dorsolateral Prefrontal Cortex." *Nature Neuroscience* 24: 425–36. https://doi.org/10.1038/s41593-020-00787-0.

McCarthy, Davis J, Kieran R Campbell, Aaron T L Lun, and Quin F Wills. 2017. "Scater: Pre-Processing, Quality Control, Normalization and Visualization of Single-Cell RNA-Seq Data in r." *Bioinformatics* 33: 1179–86. https://doi.org/10.1093/bioinformatics/btw777.

Ren, Honglei, Benjamin L. Walker, Zixuan Cang, and Qing Nie. 2022. "Identifying Multicellular Spatiotemporal Organization of Cells with SpaceFlow." *Nature Communications* 13 (4076). https://doi.org/10.1038/s41467-022-31739-w.

Salim, Agus, Dharmesh D. Bhuva, Carissa Chen, Chin Wee Tan, Pengyi Yang, Melissa J. Davis, and Jean Y. H. Yang. 2025. "SpaNorm: Spatially-Aware Normalization for Spatial Transcriptomics Data." *Genome Biology* 26 (109). https://doi.org/10.1186/s13059-025-03565-y.

Singhal, Vipul, Nigel Chou, Joseph Lee, Yifei Yue, Jinyue Liu, Wan Kee Chock, Li Lin, et al. 2024. "BANKSY Unifies Cell Typing and Tissue Domain Segmentation for Scalable Spatial Omics Data Analysis." *Nature Genetics* 56: 431–41. https://doi.org/10.1038/s41588-024-01664-3.

Totty, Michael, Stephanie C. Hicks, and Boyi Guo. 2025. "SpotSweeper: Spatially Aware Quality Control for Spatial Transcriptomics." *Nature Methods* 22: 1520–30. https://doi.org/10.1038/s41592-025-02713-3.

Traag, V. A., L. Waltman, and N. J. van Eck. 2019. "From Louvain to Leiden: Guaranteeing Well-Connected Communities." *Scientific Reports* 9 (5233). https://doi.org/10.1038/s41598-019-41695-z.

Varrone, Marco, Daniele Tavernari, Albert Santamaria-Martínez, Logan A. Walsh, and Giovanni Ciriello. 2024. "CellCharter Reveals Spatial Cell Niches Associated with Tissue Remodeling and Cell Plasticity." *Nature Genetics* 56: 74–84. https://doi.org/10.1038/s41588-023-01588-4.

Wang, Ziyi, Aoyun Geng, Hao Duan, Feifei Cui, Quan Zou, and Zilong Zhang. 2024. "A Comprehensive Review of Approaches for Spatial Domain Recognition of Spatial Transcriptomes." *Briefings in Functional Genomics* 23: 702–12. https://doi.org/10.1093/bfgp/elae040.

Zhao, Edward, Matthew R. Stone, Xing Ren, Jamie Guenthoer, Kimberly S. Smythe, Thomas Pulliam, Stephen R. Williams, et al. 2021. "Spatial Transcriptomics at Subspot Resolution with BayesSpace." *Nature Biotechnology* 39: 1375–84. https://doi.org/10.1038/s41587-021-00935-2.

Workflow: Xenium

Preamble

Introduction

In this demo, we will analyze a 313-plex Xenium dataset on human breast cancer tissue (Janesick et al. 2023). Following very basic quality control and preprocessing, we will perform both (non-spatial) unsupervised clustering as well as fully supervised label-transfer based on scRNA-seq reference data, and then compare the obtained cluster assignments to those provided by the authors.

Dependencies

```
library(dplyr)
library(tidyr)
library(scran)
library(igraph)
library(scater)
library(scuttle)
library(SingleR)
library(ggplot2)
library(patchwork)
library(OSTA.data)
library(BayesSpace)
library(BiocParallel)
library(DropletUtils)
library(SpatialExperiment)
library(SpatialExperimentIO)
# set parallelization
bp <- MulticoreParam(4)
# set seed for random number generation
# in order to make results reproducible
set.seed(112358)
```

```
# retrieve dataset from OSF repository
id <- "Xenium_HumanColon_Oliveira"
pa <- OSTA.data_load(id)
dir.create(td <- tempfile())
unzip(pa, exdir=td)
(spe <- readXeniumSXE(td))
```

```
## class: SpatialExperiment
## dim: 422 340837
## metadata(4): experiment.xenium transcripts cell_boundaries
##   nucleus_boundaries
## assays(1): counts
## rownames(422): ABCC8 ACP5 ... WFDC2 XCL2
## rowData names(3): ID Symbol Type
## colnames(340837): aaaadaba-1 aaaadgga-1 ... oikdmkkf-1 oikeebja-1
## colData names(10): cell_id transcript_counts ... nucleus_area
##   sample_id
## reducedDimNames(0):
## mainExpName: NULL
## altExpNames(3): NegControlProbe UnassignedCodeword
##   NegControlCodeword
## spatialCoords names(2) : x_centroid y_centroid
## imgData names(0):
```

```
.plt_xy <- \(spe, col) {
    df <- data.frame(colData(spe), spatialCoords(spe))
    aes <- if (is.numeric(df[[col]])) {
        theme(
            legend.key.height=unit(1, "lines"),
            legend.key.width=unit(0.5, "lines"))
    } else {
        list(
            theme(legend.key.size=unit(0, "lines")),
            guides(col=guide_legend(override.aes=list(alpha=1, size=2))))
    }
    ggplot(df, aes(x_centroid, y_centroid, col=.data[[col]])) +
        coord_equal() + theme_void() + aes +
        geom_point(stroke=0, size=1/3)
}
```

Quality control

```
# compute cell-level QC metrics
spe <- addPerCellQCMetrics(spe)
# identify low-quality cells by thresholding on
# median absolute deviation (MAD) from the median
ol <- perCellQCFilters(spe)
# tabulate # and % of cells discarded
# due to few counts/detected features
data.frame(
    check.names=FALSE,
    `#`=apply(ol, 2, sum),
    `%`=round(100*apply(ol, 2, mean), 2))
```

```
##                    #    %
## low_lib_size    8374 2.46
```

```
##  low_n_features 15313 4.49
##  discard        15313 4.49
```

Before proceeding to exclude any cells from downstream analyses, let's first visualize the cells deemed to be of low quality in space alongside the underlying quality control metrics (total counts and detected features):

```
spe$ol <- ol$discard
.plt_xy(spe, "sum") +
    scale_color_viridis_c(
        "# counts",
        trans="log1p",
        breaks=range(spe$sum),
        labels=c("low", "high")) +
.plt_xy(spe, "detected") +
    scale_color_viridis_c(
        "# features",
        breaks=range(spe$detected),
        labels=c("low", "high")) +
.plt_xy(spe[, order(spe$ol)], "ol") +
    scale_color_manual(
        "low-quality",
        labels=c("no", "yes"),
        values=c("lavender", "purple"))
```

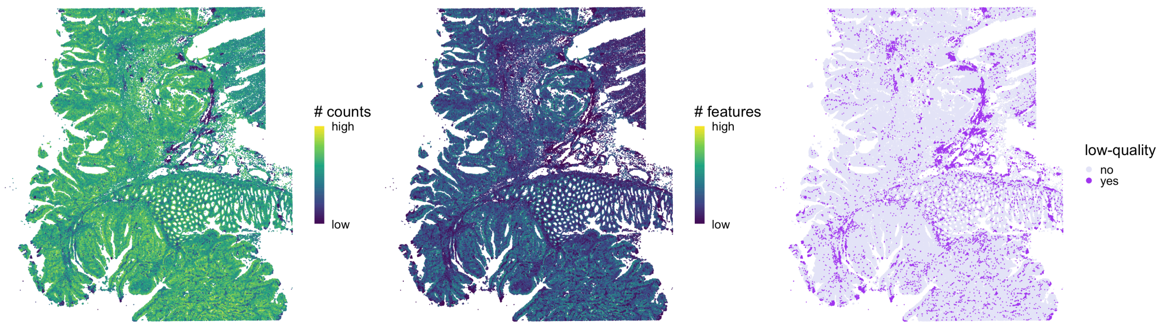


```
# discard low-quality cells
ncol(spe <- spe[, !ol$discard])

##  [1] 325524
```

Processing

For the sake of runtime, we will perform downstream analyses only on a square crop of the tissue, defined by the following px coordinates:

```
box <- list(xmin=2e3, xmax=5e3, ymin=1e3, ymax=4e3)
```

Cropping to this region, we retain fewer than 90,000 cells:

```
xy <- spatialCoords(spe)
i <-
    xy[, 1] > box$xmin &
    xy[, 1] < box$xmax &
    xy[, 2] > box$ymin &
    xy[, 2] < box$ymax
ncol(sub <- spe[, i])
```

```
## [1] 88863
```

```
df <- data.frame(xy, i)
p <- ggplot(df,
    aes(x_centroid, y_centroid)) +
    coord_equal() + theme_void() +
    theme(legend.position="none")
p + geom_point(aes(col=i), stroke=0, size=0.1) |
p + geom_point(data=df[i, ], stroke=0, size=0.2)
```

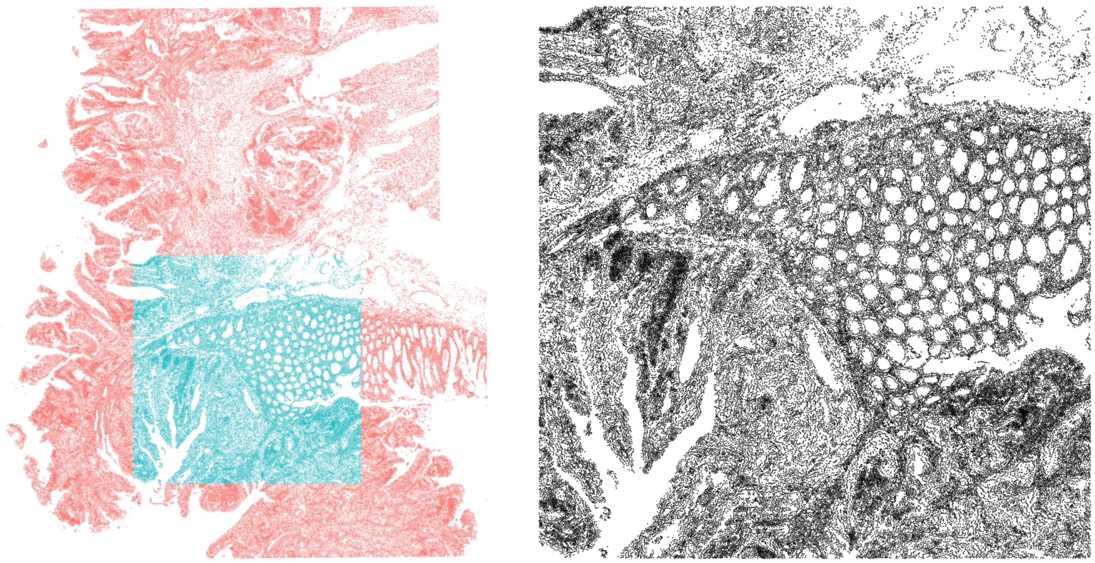

Next, we'll log-normalize counts by area, and perform principal component analysis (PCA) on all 422 RNA targets:

```
# cell area-based normalization
logcounts(sub) <- sweep(assay(sub), 2, sub$cell_area, `/`)
# principal component analysis
sub <- runPCA(sub) #, BPPARAM=bp)
```

Let's visualize the expression of some genes in space; e.g., PIGR, IGHG3 and CEACAM6, which should mark epithelial, plasma and tumor cells, respectively:

```r
gs <- c("PIGR", "IGHG3", "CEACAM6")
es <- scale(logcounts(sub))
es <- t(as.matrix(es[gs, ]))
colData(sub) <- cbind(colData(sub), es)
ps <- lapply(gs, \(.) .plt_xy(sub, .) + ggtitle(.))
wrap_plots(ps, nrow=1) &
    scale_color_gradientn(NULL,
        labels=c("low", "high"),
        colors=rev(hcl.colors(9, "PuRd")),
        limits=rng, breaks=rng <- range(es)) &
    theme(plot.title=element_text(hjust=0.5))
```

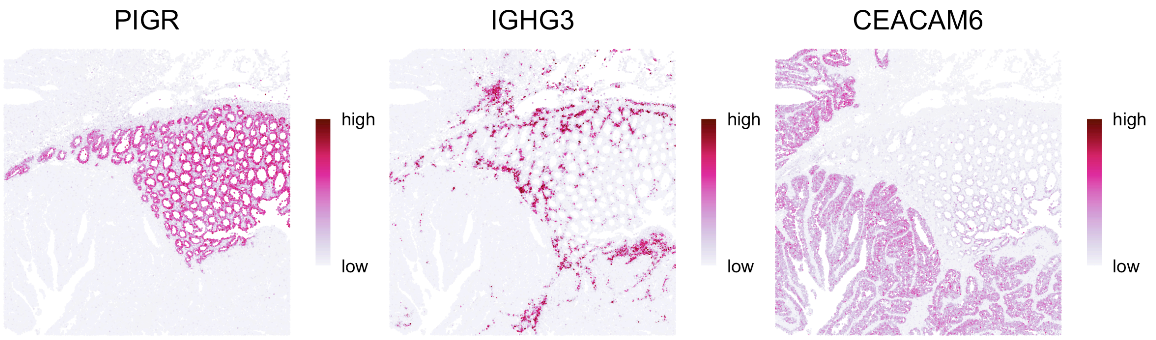

Annotation

Unsupervised

```r
# shared nearest-neighbor (SNN) graph based on
# cell-to-cell Jaccard similarity in PC space
g <- buildSNNGraph(sub, use.dimred="PCA", type="jaccard", BPPARAM=bp)
# community detection using Leiden algorithm
k <- cluster_leiden(g, objective_function="modularity", resolution=0.7)
table(sub$Leiden <- factor(. <- k$membership, labels=letters[seq_along(unique(.))]))
```

```
##
##      a     b     c     d     e     f     g     h     i     j
##   5853 30425  4105  7709  7409  3877 11252  6026  9937  2270
```

Supervised

For comparison, we annotate the Xenium data using a label transfer approach, *SingleR*, which relies on labeled scRNA-seq data to compute references profiles and transfers labels based on the rank correlation between observed (here, Xenium) and reference (scRNA-seq) expression profiles.

First, we retrieve a matching (Chromium) scRNA-seq dataset, which includes low- (`Level1`) and high-resolution (`Level2`) annotations of cells into 9 and 31 subpopulations, respectively:

```
# retrieve dataset from OSF repository
id <- "Chromium_HumanColon_Oliveira"
pa <- OSTA.data_load(id)
dir.create(td <- tempfile())
unzip(pa, exdir=td)
# read into 'SingleCellExperiment'
sce <- read10xCounts(list.files(td, "h5$", full.names=TRUE))
cd <- read.csv(list.files(td, "cell_meta", full.names=TRUE))
colData(sce) <- cbind(colData(sce), cd[, -1])
table(sce$Level1) # tabulate low-res. labels
ncol(sce) # overall number of cells
```

```
##
##                 B cells           Endothelial            Fibroblast
##                   33611                  7969                 28653
##  Intestinal Epithelial               Myeloid              Neuronal
##                   22763                 25105                  4199
##             QC_Filtered        Smooth Muscle               T cells
##                   19103                 43308                 29272
##                   Tumor
##                   65626
##  [1] 279609
```

Here, we run `SingleR` using `Level2` (high-resolution) annotations and with argument `aggr.ref=TRUE`, such that reference profiles will be aggregated (per cluster) prior to annotation. In this way, every Xenium cell will be assigned a label based on which pseudo-bulk scRNA-seq profile represents the best match.

Note that we filter the reference data to contain only cells from the same patient. This is not strictly necessary, assuming that clusters are transcriptionally stable across patients, but is done here to reduce runtime.

```
# exclude cells deemed to be of low-quality
sce <- sce[, sce$QCFilter == "Keep"]
# subset cells from same patient
sce <- sce[, grepl("P2", sce$Patient)]
# realize count matrix
assay(sce) <- as(assay(sce), "dgCMatrix")
# log-library size normalization
sce <- logNormCounts(sce)
# restrict to Xenium targets
sce <- sce[rowData(sce)$ID %in% rowData(sub)$ID, ]
# set gene symbols as feature names
rownames(sce) <- rowData(sce)$Symbol
# perform label transfer at the single cell-level,
# using pseudo-bulk Chromium profiles as reference
res <- SingleR(
    test=sub, ref=sce,
    labels=sce$Level2,
    aggr.ref=TRUE, BPPARAM=bp)
sub$Level2 <- factor(res$pruned.labels)
```

Based on these predictions, we can also propagate `Level1` (low-resolution) annotations:

```
idx <- match(sub$Level2, sce$Level2)
table(sub$Level1 <- factor(sce$Level1[idx]))
```

```
##
##               B cells           Endothelial            Fibroblast
##                  6375                  4799                 10136
##  Intestinal Epithelial               Myeloid              Neuronal
##                 11947                  7229                   418
##          Smooth Muscle               T cells                 Tumor
##                  4404                  5040                 38113
```

Simplifying further, we can group cells into different compartments, namely, (malignant) tumor, immune, epithelial and stromal cells; we'll see below that visualizing cells in this way nicely captures the general tissue structure.

```
lab <- list(
    tum=c("Tumor"),
    epi=c("Intestinal Epithelial"),
    imm=c("B cells", "T cells", "Myeloid"),
    str=c("Endothelial", "Fibroblast", "Smooth Muscle"))
idx <- match(sub$Level1, unlist(lab))
lab <- rep.int(names(lab), sapply(lab, length))
table(sub$Level0 <- factor(lab[idx]))
```

```
##
##    epi   imm   str   tum
##  11947 18644 19339 38113
```

Comparison

Tabulating the cluster assignments between Leiden (unsupervised) and `SingleR` (supervised), we can observe overall high concordance; i.e., most clusters have a one-to-one mapping between both approaches. However, some subpopulations are split between clusters; e.g., cells labeled as cluster fibroblasts, endothelia and smooth muscle cells by `SingleR` tend to intermix in the Leiden clusters. This is not unexpected, given that these are all stromal subpopulations with comparatively similar transcriptional profiles. (Note that we are observing a mere fraction of the whole transcriptome with the Xenium panel employed here.)

```
# contingency table & number of clusters
round(100*prop.table(table(sub$Level1, sub$Leiden), 2), 1)
```

```
##
##                            a    b    c    d    e    f    g    h    i    j
##    B cells               2.0  0.0  0.0 75.0  3.2  1.2  0.5  2.3  0.1  0.0
##    Endothelial           1.1  0.0  0.1  2.3  3.7  1.5 35.1  4.7  0.1  0.0
##    Fibroblast            6.1  0.0  0.1  6.5 15.0 47.9 50.5 10.1  0.4  0.0
##    Intestinal Epithelial 0.2  0.1  0.1  0.2  0.9  0.0  0.1  7.4 92.2 99.3
##    Myeloid               4.6  0.1  0.3  5.5 65.6  1.1  1.3 22.7  1.4  0.1
##    Neuronal              0.1  0.0  0.0  0.4  0.7  0.1  0.3  1.7  1.9  0.1
```

```
##    Smooth Muscle           2.6  0.0  0.1  2.8  5.8 45.1 10.1 10.8  0.9  0.0
##    T cells                69.2  0.0  0.1  3.4  2.8  2.6  0.8  4.1  0.8  0.1
##    Tumor                  14.1 99.7 99.2  4.0  2.4  0.4  1.1 36.2  2.3  0.4
```

```
c(SingleR=nlevels(sub$Level1), Leiden=nlevels(sub$Leiden))
```

```
##  SingleR  Leiden
##        9      10
```

```
lapply(c("Leiden", "Level0", "Level1"), \(.) {
    pal <- if (. == "Level0") {
        c("gold", "cyan", "magenta", "black")
    } else {
        hcl.colors(nlevels(sub[[.]]), "Spectral")
    }
    .plt_xy(sub, .) + scale_color_manual(values=pal)
}) |> wrap_plots()
```

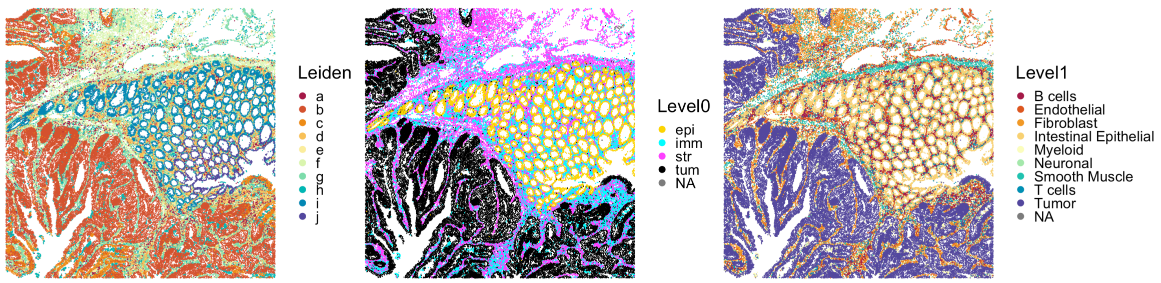

Downstream

Marker genes

Below, we test for differential expression between `Level1` clusters, and visualize selected markers as a heatmap of (z-scaled) average expression. It's comforting to see that we pick up on many classics, e.g., endothelia are marked by VWF and PECAM1, T cells by CD2 and TRAC, etc.

```
# test for differential expression between clusters
mgs <- findMarkers(sub, groups=sub$Level1, direction="up")
# select top-ranked genes for every cluster
top <- unique(unlist(lapply(mgs, \(df) rownames(df)[df$Top <= 3])))
plotGroupedHeatmap(sub,
    features=top, group="Level1",
    scale=TRUE, center=TRUE, fontsize=6)
```

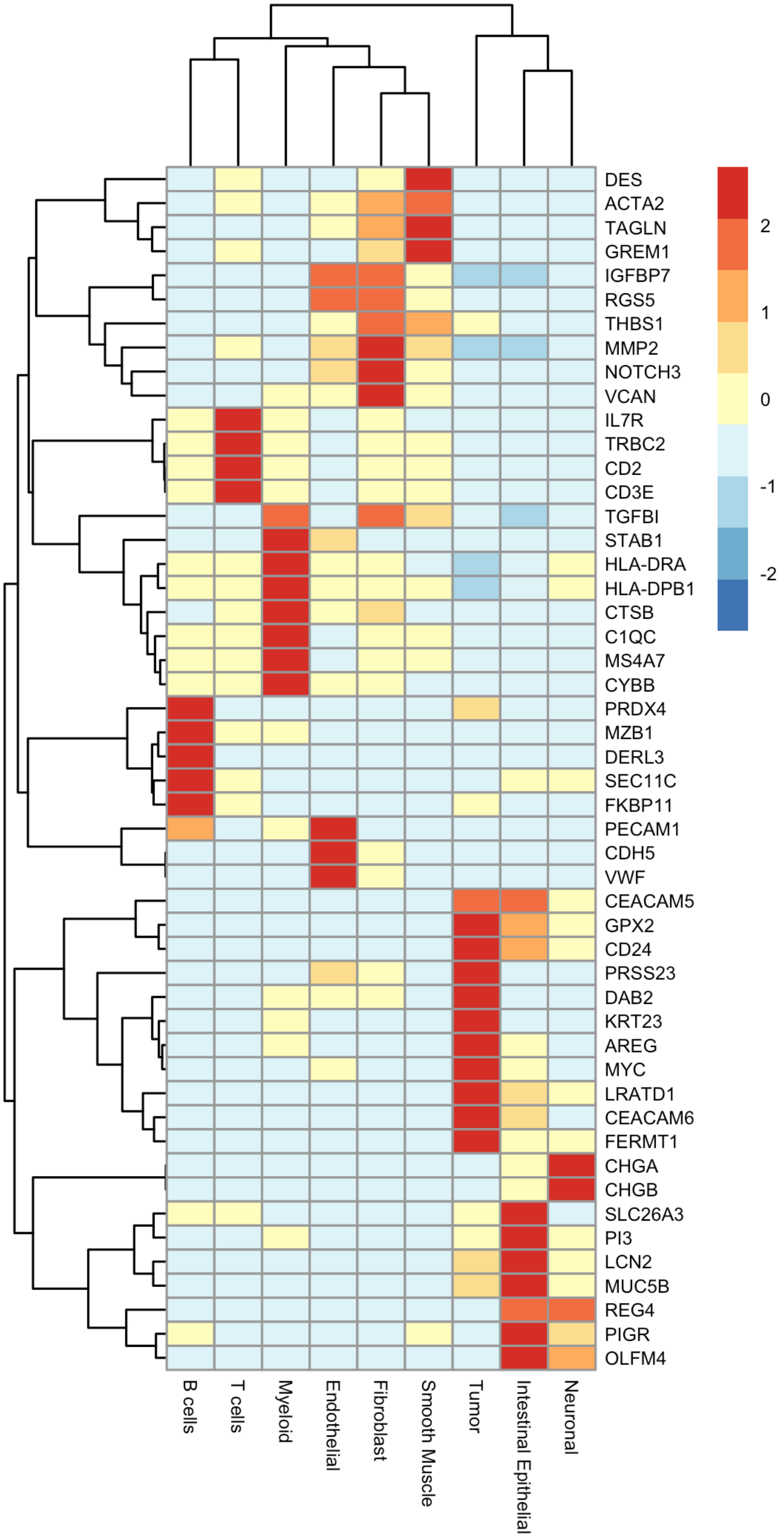

DES
ACTA2
TAGLN
GREM1
IGFBP7
RGS5
THBS1
MMP2
NOTCH3
VCAN
IL7R
TRBC2
CD2
CD3E
TGFBI
STAB1
HLA-DRA
HLA-DPB1
CTSB
C1QC
MS4A7
CYBB
PRDX4
MZB1
DERL3
SEC11C
FKBP11
PECAM1
CDH5
VWF
CEACAM5
GPX2
CD24
PRSS23
DAB2
KRT23
AREG
MYC
LRATD1
CEACAM6
FERMT1
CHGA
CHGB
SLC26A3
PI3
LCN2
MUC5B
REG4
PIGR
OLFM4
B cells
T cells
Myeloid
Endothelial
Fibroblast
Smooth Muscle
Tumor
Intestinal Epithelial
Neuronal
2
1
0
-1
-2

Appendix

References

Janesick, Amanda, Robert Shelansky, Andrew D. Gottscho, Florian Wagner, Stephen R. Williams, Morgane Rouault, Ghezal Beliakoff, et al. 2023. "High Resolution Mapping of the Tumor Microenvironment Using Integrated Single-Cell, Spatial and in Situ Analysis." *Nature Communications* 14 (8353). https://doi.org/10.1038/s41467-023-43458-x.

Workflow: CosMx

Preamble

Introduction

In this demo, we will analyze a 1k-plex CosMx data (Bruker) of a mouse brain sample; see Chapter 1. Following quality control and preprocessing, we will perform cell type annotation, identification of marker genes, and cell-cell interaction analysis.

Dependencies

```r
library(DESpace)
library(ggplot2)
library(OSTA.data)
library(patchwork)
library(pheatmap)
library(scater)
library(scrapper)
library(scRNAseq)
library(sf)
library(SingleR)
library(SpaceTrooper)
library(SpatialExperiment)
library(SpatialExperimentIO)
library(spatialFDA)
library(SpatialFeatureExperiment)
library(spdep)
library(Voyager)
# set seed for random number generation
# in order to make results reproducible
set.seed(112358)
```

```r
# retrieve CosMx dataset from OSF repo
id <- "CosMx1k_MouseBrain2"
pa <- OSTA.data_load(id, mol=FALSE)
dir.create(td <- tempfile())
unzip(pa, exdir=td)
cos <- readCosmxSXE(td, addTx=FALSE)
# prepare data for 'SpaceTrooper'
cos <- updateCosmxSPE(cos, td, sampleName="CosMx")
cos <- readAndAddPolygonsToSPE(cos)
```

```
cos$in_tissue <- TRUE
cos
```

```
##  class: SpatialExperiment
##  dim: 950 48556
##  metadata(4): fov_positions fov_dim polygons technology
##  assays(1): counts
##  rownames(950): Chrna4 Slc6a1 ... Cck Aqp4
##  rowData names(0):
##  colnames(48556): f1_c1 f1_c10 ... f99_c98 f99_c99
##  colData names(22): fov cellID ... polygons in_tissue
##  reducedDimNames(0):
##  mainExpName: NULL
##  altExpNames(1): NegPrb
##  spatialCoords names(2) : CenterX_global_px CenterY_global_px
##  imgData names(1): sample_id
```

Quality control

We can have an overview of the data, by plotting the spatial distribution of the cells, represented by their centroids. This is useful to have a global bird's-eye-view of the tissue.

```
plotCentroids(cos)
```

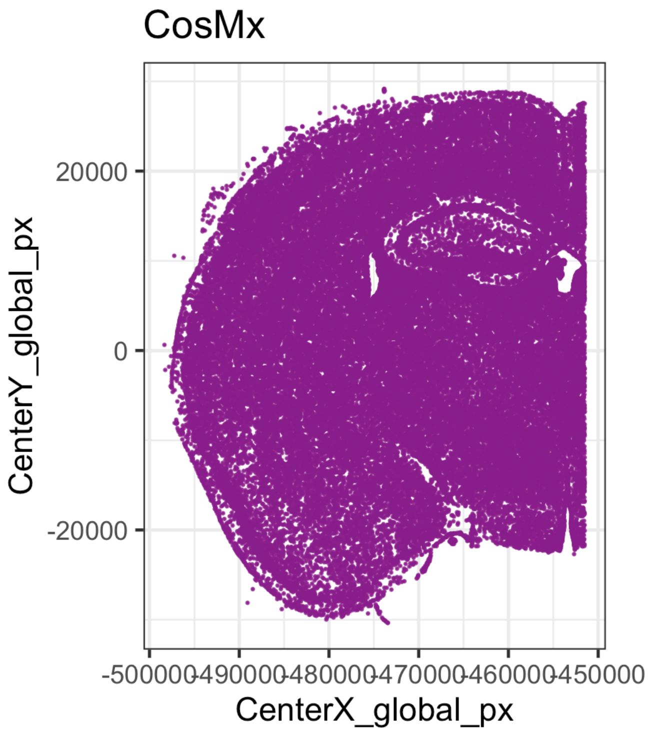

Unlike, Xenium and MERSCOPE, CosMx does not stitch fields of view (FOVs) in a single image, but rather treats each FOV independently. It is therefore useful to visualize a map of the FOVs to understand their spatial arrangement.

```
plotCellsFovs(cos)
```

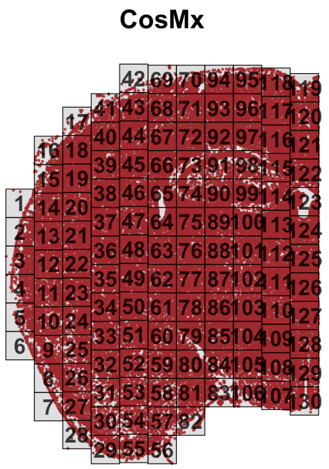

Chapter 2 gives a thorough overview of quality control steps for imaging-based spatial transcriptomics data, including CosMX-specific metrics. Here, we will simply compute *SpaceTrooper*'s aggregated QC score and keep the cells that pass the threshold.

```
lys <- c("NegPrb", "Negative", "SystemControl")
cos <- spatialPerCellQC(cos, rmZeros=TRUE, negProbList=lys)
cos <- computeQCScore(cos)
plotCentroids(cos, colourBy="QC_score")
```

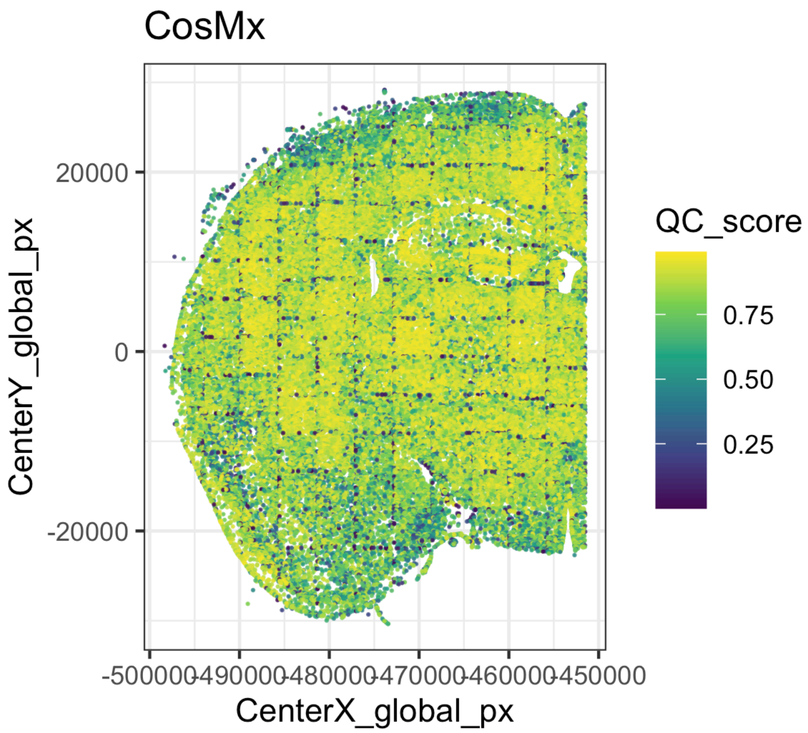


```
cos <- computeQCScoreFlags(cos, qsThreshold=0.5)
plotCentroids(cos, colourBy="low_qcscore") +
```

```
    theme(legend.key.size=rel(0)) +
    guides(col=guide_legend(override.aes=list(size=2)))
```

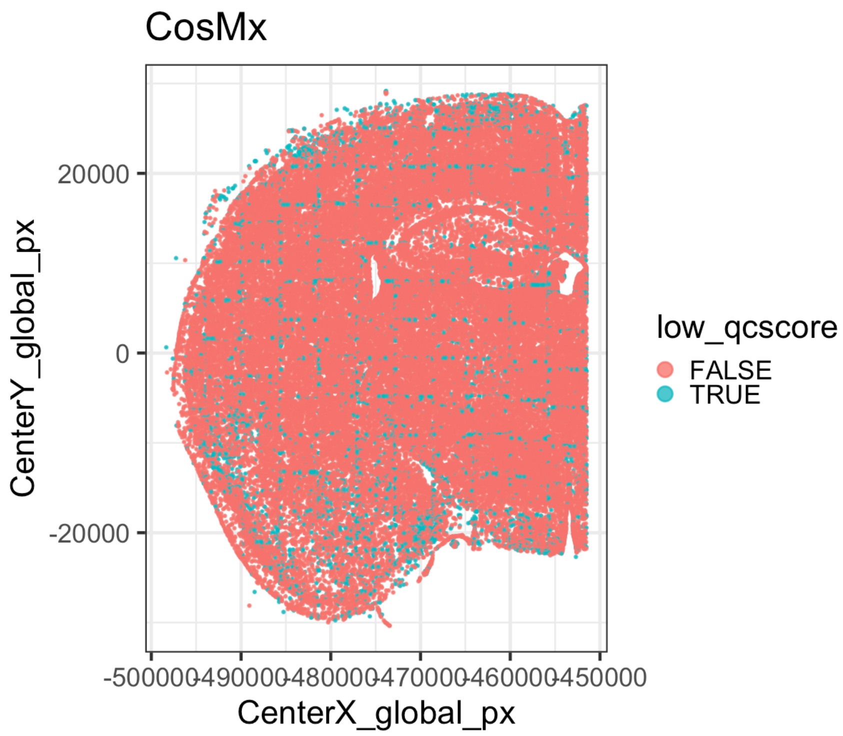


```
cos <- cos[, !cos$low_qcscore]
```

Processing

For the sake of runtime, we will perform downstream analyses only on a small portion of the tissue, defined by the following FOVs:

```
fs <- c(72:74, 90:92, 97:99, 114:116)
plotZoomFovsMap(cos, fovs=fs)
```

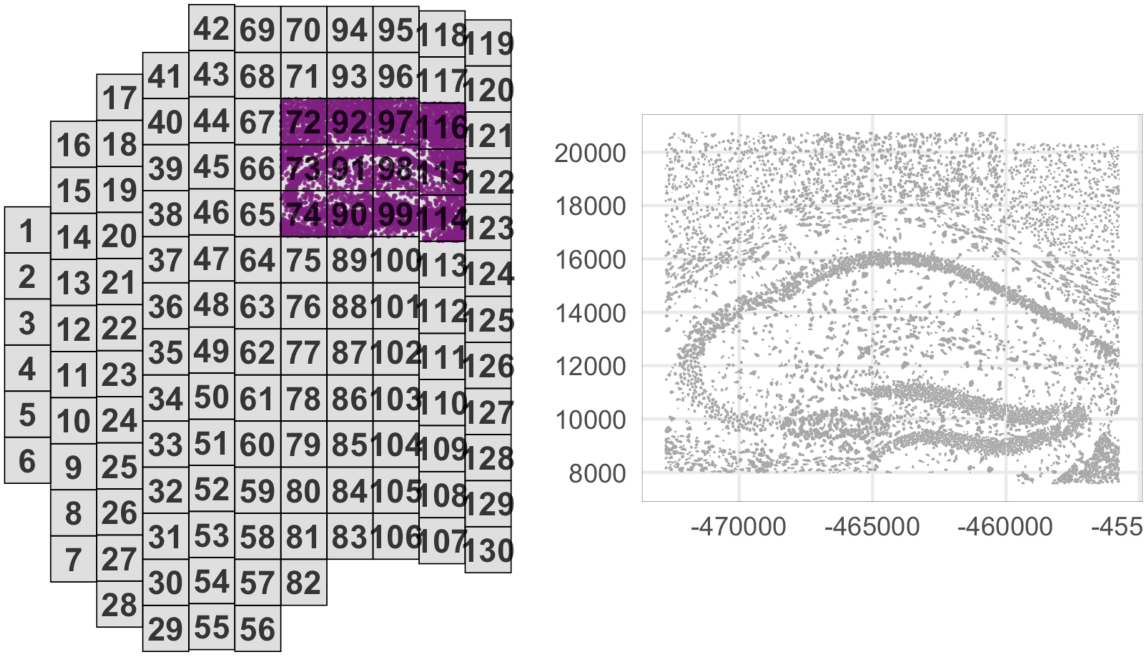


```
sub <- cos[, cos$fov %in% fs]
```

Next, we'll log-normalize counts by area, and perform principal component analysis (PCA) on all 950 RNA targets:

See Chapter 3 for more details on normalization.

```
# cell area-based normalization
logcounts(sub) <- sweep(assay(sub), 2, sub$Area, `/`)
# principal component analysis
sub <- runPCA(sub)
```

Let's visualize the expression of the first two principal components (PCs) in the spatial context.

```
# add PCs as cell metadata
pcs <- reducedDim(sub, "PCA")
colData(sub) <- cbind(colData(sub), pcs)
# visualize PCs 1 & 2 in space
plotCentroids(sub, colourBy="PC1") +
plotCentroids(sub, colourBy="PC2")
```

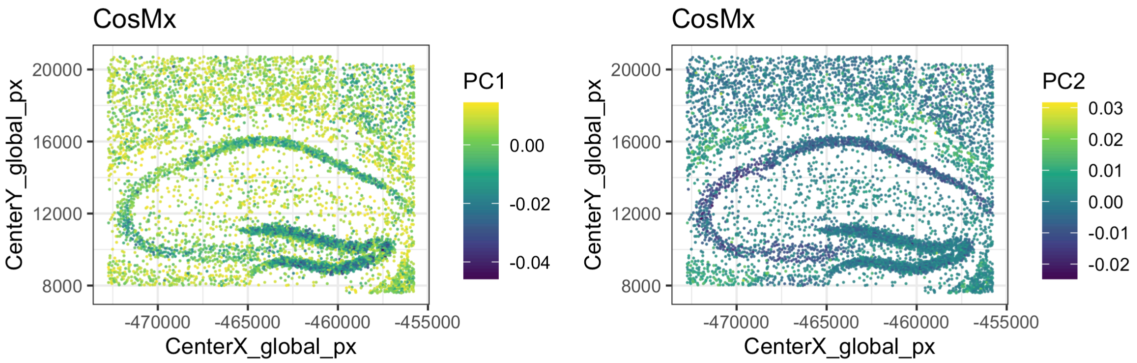

Annotation

Here, we use the Zeisel et al. (2015) dataset as a reference to annotate the cell types with *SingleR*.

```
sceZ <- ZeiselBrainData()
sceZ <- logNormCounts(sceZ)
```

```
pred <- SingleR(test=sub, ref=sceZ, labels=sceZ$level1class, de.method="wilcox")
sub$SingleR_label <- factor(pred$pruned.labels)

nk <- nlevels(sub$SingleR_label)
pal <- hcl.colors(nk, "Spectral")

plotPolygons(sub,
    colourBy="SingleR_label") +
    scale_fill_manual(values=pal)
```

CosMx

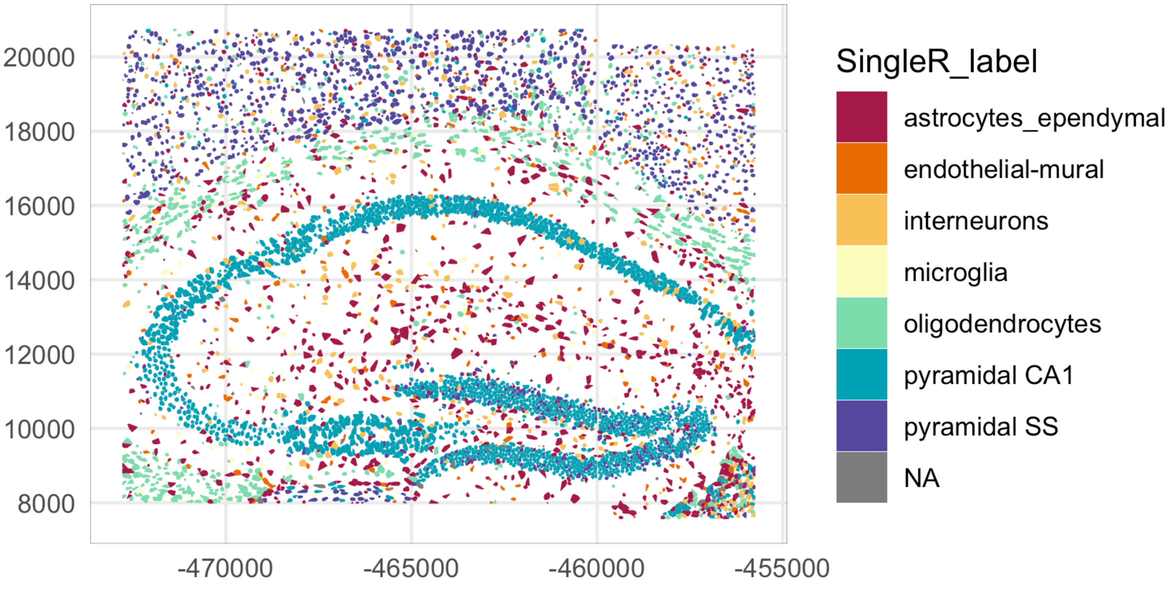

Note that the annotation performed here is a transfer label from a reference dataset that only partially matches with the tissue under study and purposely annotated at a coarse level (level 1). Ideally, one can use a more appropriate reference dataset, or better yet, build a custom reference from single-cell RNA-seq data of the same tissue.

Marker genes

Here, we identify cluster-related spatially-variable genes using the *DESpace* package (Cai, Robinson, and Tiberi 2024). In this workflow, we use this as a way to identify marker genes for the cell types annotated above.

A different, often preferable, approach consists in using, in addition to the gene expression profiles, the image data and the spatial context of the cells. *InSituType* (Danaher et al. 2022) provides a framework to perform such analysis in unsupervised, semi-supervised, or supervised manners. Please refer to its documentation for more details.

```
res <- svg_test(spe=sub, cluster_col="SingleR_label")
head(res$gene_results)
```

```
##          gene_id       LR   logCPM PValue FDR
## Itm2a      Itm2a 2596.806 11.03858      0   0
## Flt1        Flt1 2601.591 11.05385      0   0
## Cldn5      Cldn5 2825.316 11.11884      0   0
## Gja1        Gja1 4982.209 11.29054      0   0
## Gpr37l1 Gpr37l1 4989.937 11.30343      0   0
## Bcan        Bcan 3567.078 11.61162      0   0
```

We focus on the genes with the smallest p-values, and compute the average expression of each gene in each cell type. We can visualize this with a heatmap.

```
df <- res$gene_results
gs <- df$gene_id[rank(df$PValue, ties.method="min") == 1]
mu <- t(apply(counts(sub)[gs, ], 1, tapply, sub$SingleR_label, mean))

pheatmap(t(mu),
    scale="column", show_colnames=FALSE,
    cluster_rows=TRUE, cluster_cols=TRUE,
    main="Average expression of top SVGs per cell type")
```

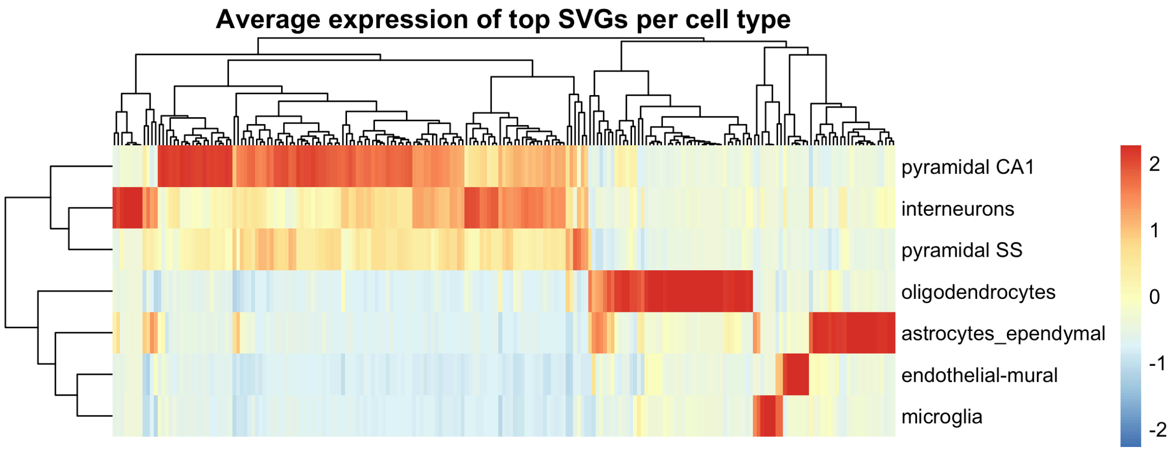

We can also visualize the spatial distribution of some of these genes, e.g., Mbp that marks oligodendrocytes, and Calm1, Calm2, and Snap25 that mark different subsets of neurons. Note also how some genes show a difference of expression within the pyramidal neuron population, suggesting the presence of subtypes.

```
gs <- c("Mbp", "Calm1", "Calm2", "Snap25")
ps <- lapply(gs, \(g) {
    sub[[g]] <- logcounts(sub)[g, ]
    plotPolygons(sub, colourBy=g) + ggtitle(g)
})
dy <- range(logcounts(sub)[gs, ])
wrap_plots(ps, guides="collect") &
    scale_fill_viridis_c(
        "expression",
        limits=dy, breaks=dy,
        labels=c("low", "high")) &
    theme(plot.title=element_text(hjust=0.5))
```

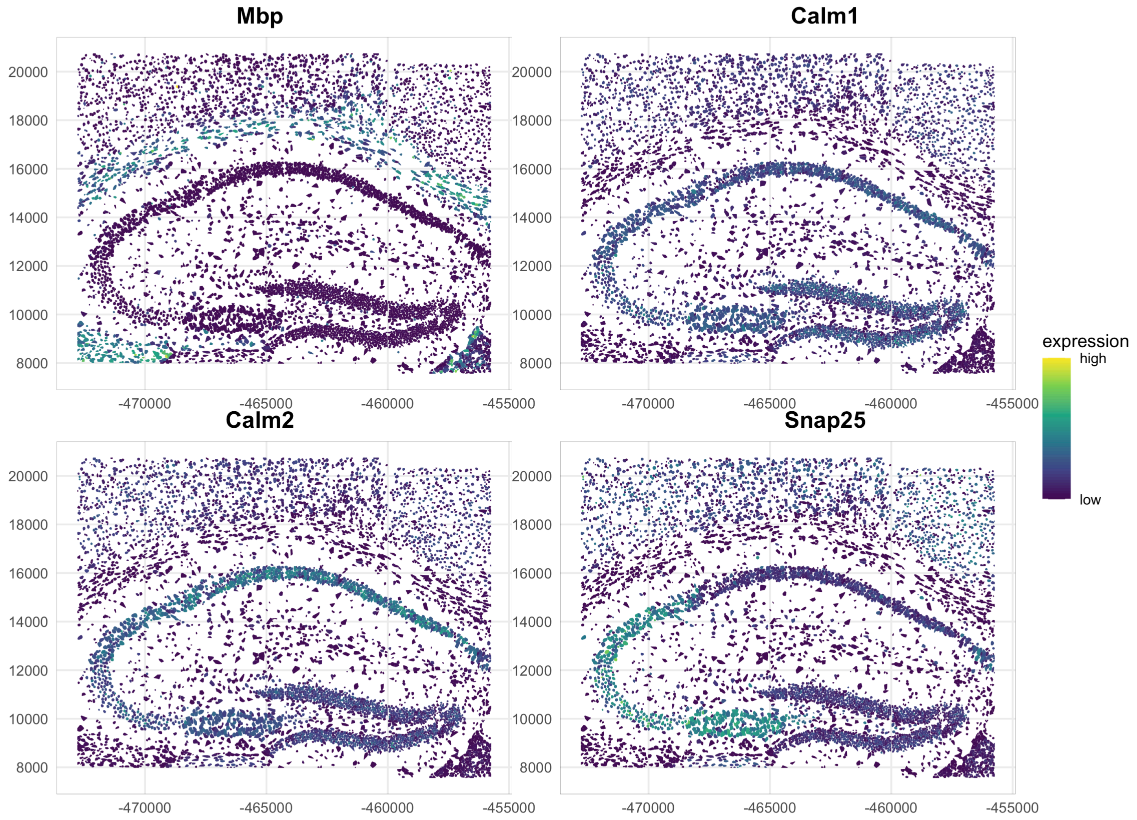

Neighborhood-based gene expression analyses

We start by creating a cell neighborhood graph, based on a distance threshold of 50 microns. To do so, we use the *SpatialFeatureExperiment* package (Moses et al. 2023).

Note that an alternative approach uses the k nearest neighbors (kNN) method to define the neighborhood graph. Here, we prefer to use a distance-based approach, as it better captures the morphological structure of this tissue area.

```
# convert to SFE & remove cells with NA labels
sfe <- as(sub, "SpatialFeatureExperiment")
sfe <- sfe[, !is.na(sfe$SingleR_label)]

# this is needed for Voyager's plotting functions
colnames(sfe$polygons)[6] <- "geometry"
st_geometry(sfe$polygons) <- "geometry"
colGeometry(sfe, "cellSeg") <- sfe$polygons

colGraph(sfe, "poly2nb") <-
    findSpatialNeighbors(sfe,
        type="cellSeg",
        method="poly2nb",
        style="W", snap=50)

plotColGraph(sfe,
    colGraphName="poly2nb",
    colGeometryName="cellSeg") +
    theme_void()
```

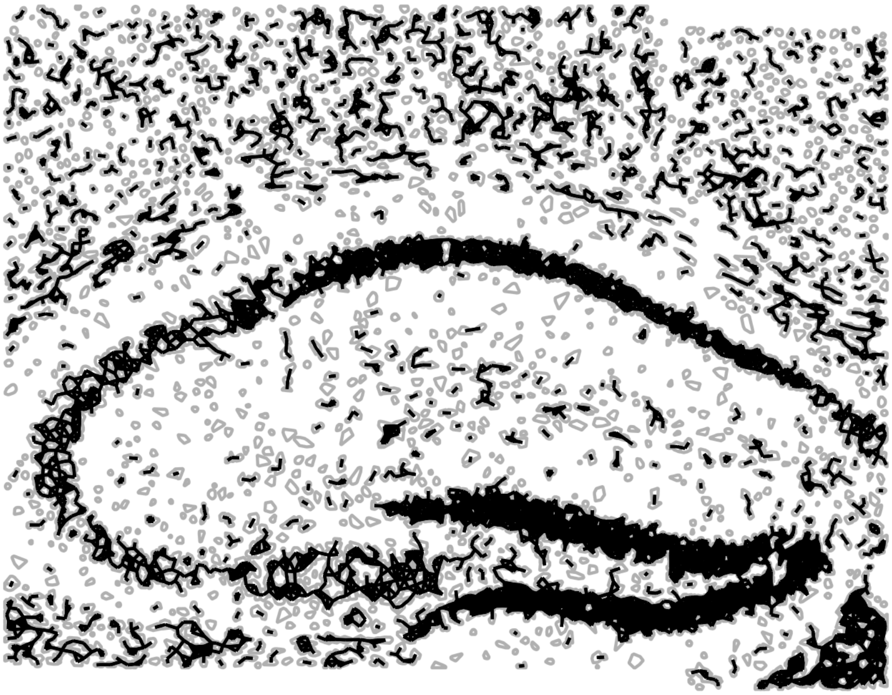

Given the neighborhood graph, we can calculate local measures of spatial autocorrelation for specific genes in the dataset.

Here, we focus on Snap25 and Calm1, neuronal marker genes identified above that show interesting spatial expression distributions (see above). First, we compute the local Moran's I coefficient.

```
gs <- c("Snap25", "Calm1")
sfe <- runUnivariate(sfe,
    features=gs,
    type="localmoran",
    zero.policy=TRUE,
    colGraphName="poly2nb",
    colGeometryName="cellSeg")

plotLocalResult(sfe,
    name="localmoran",
    features=gs, ncol=2,
    colGeometryName="cellSeg",
    divergent=TRUE, diverge_center=0)
```

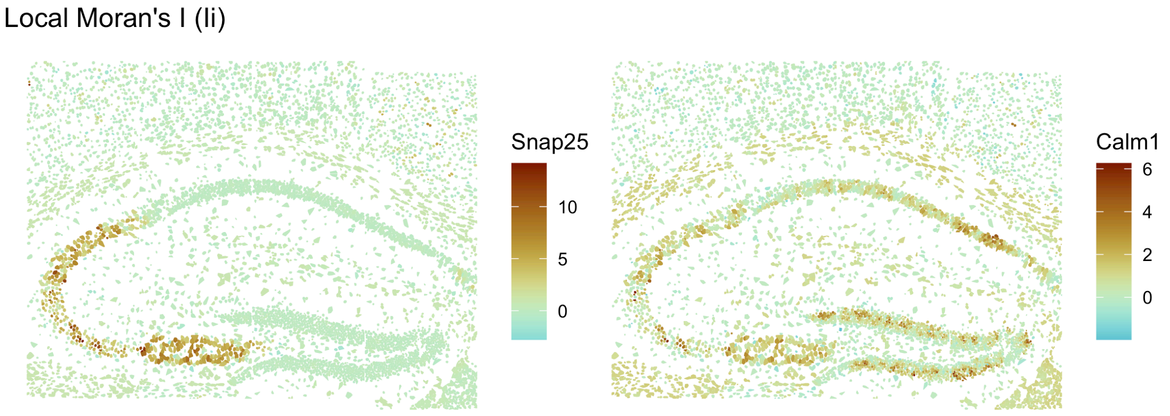

From the plots, we can see that Snap25 shows positive spatial autocorrelation in a very localized part of the tissue, while Calm1 shows a more diffuse autocorrelation structure.

We focus on Snap25 and visualize its autocorrelation structure using a Moran scatter plot, which shows the relationship between the expression of the gene in each cell and the average expression in its neighbors.

```
sfe <- runUnivariate(sfe,
    feature=gs[1],
    zero.policy=TRUE,
    type="moran.plot",
    colGraphName="poly2nb",
    colGeometryName="cellSeg")

moranPlot(sfe,
    feature=gs[1],
    graphName="poly2nb",
    swap_rownames="symbol")
```

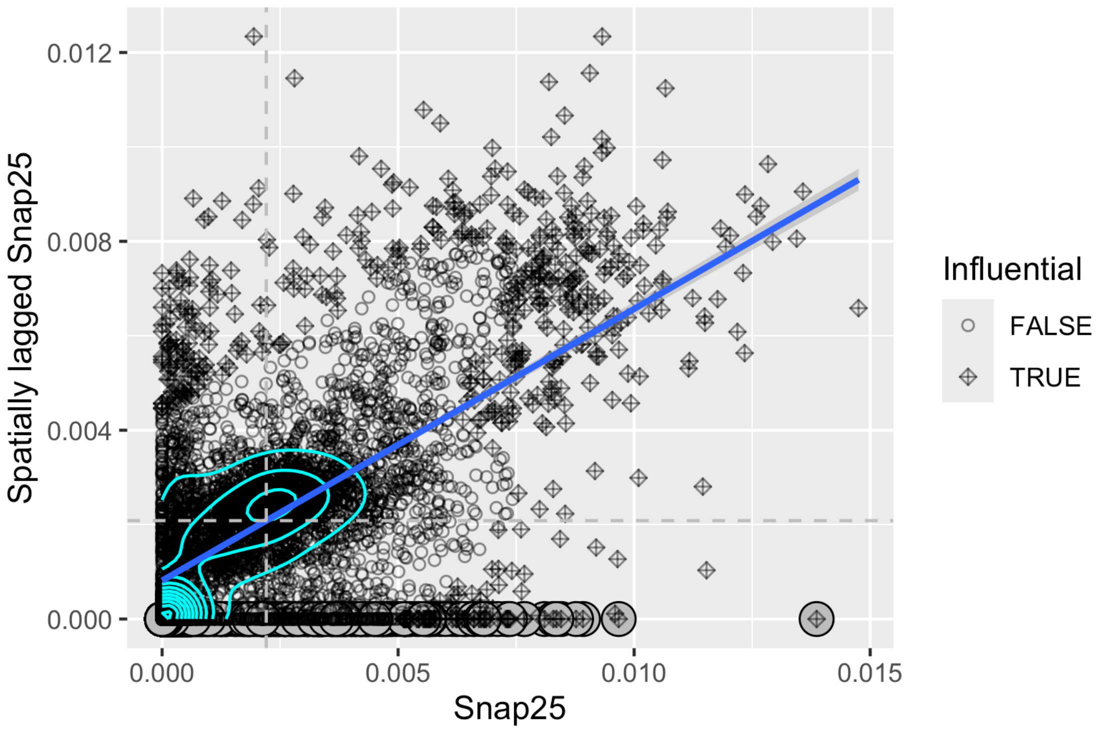

We can then classify cells based on the significance of their local Moran's I values, and visualize the resulting clusters in the spatial context.

```
res <- localResults(sfe)[["localmoran"]][[1]]
res$locClust <- with(res, ifelse(
    `-log10p_adj` > -log10(0.05),
    as.character(mean), "non-siginificant"))
localResults(sfe)[["localmoran"]][[1]] <- res

plotLocalResult(sfe,
    features=gs[1],
    name="localmoran",
    attribute="locClust",
    colGeometryName="cellSeg")
```

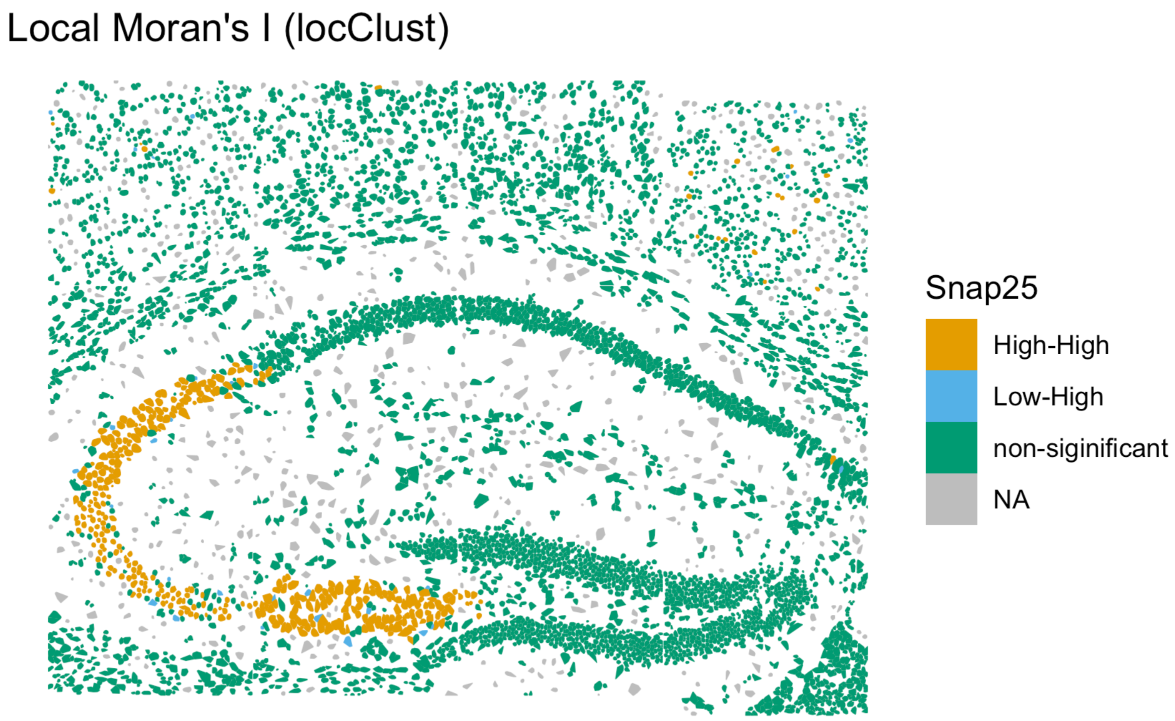

In this case, we can see a significant positive autocorrelation in the lower left part of the structure, while the rest of the tissue does not show significant autocorrelation.

Note that other local spatial autocorrelation measures, such as Geary's C coefficient and Getis-Ord statistic, as well as their global versions can be used for similar analyses Moses et al. (2023).

Cell-cell interactions

Joint counts

We can also use the neighborhood graph to investigate cell-cell interactions between different cell types annotated above. Here, we use the join count statistic implemented in the *spdep* package to quantify the tendency of cells of different types to be neighbors.

```
jc <- joincount.multi(sfe$SingleR_label, colGraph(sfe, "poly2nb"), zero.policy=TRUE)
head(jc[order(abs(jc[, "z-value"]), decreasing=TRUE), ])
```

```
##                                      Joincount   Expected  Variance   z-value
## Jtot                                1132.15707 2494.52543 265.87369 -83.55200
## pyramidal CA1:pyramidal CA1          735.66944  351.21205  62.92504  48.46592
## pyramidal CA1:oligodendrocytes        22.39901  303.28546  75.62290 -32.30012
## oligodendrocytes:oligodendrocytes    196.71667   65.35907  19.34973  29.86192
## pyramidal CA1:astrocytes_ependymal    97.55301  379.29404  92.67327 -29.26664
## pyramidal SS:pyramidal CA1           195.38757  445.19310 106.83889 -24.16781
```

We can see that there is a strong tendency for cells of the same type to be neighbors (high positive z-values). There are also some interesting negative interactions between different cell types: e.g., pyramidal CA1 tend to be away from oligodendrocytes and astrocytes. This highlights the compartimentalized structure of the brain tissue.

Point-pattern analysis

A similar result can be obtained using point-pattern analysis, such as Ripley's K function, and similar methods, using for instance the *spatialFDA* package.

Here, we use Besag's L function to quantify the interaction between pyramidal CA1 neurons, and the interaction between them and oligodendrocytes (cross-L function).

Other methods are available, such as Ripley's K function, and the pair correlation function (Emons et al. 2025). Note that here we are making the quite strong assuption of homogeneity of the point pattern, which is irrealistic in tissue biology. In particular, CSR is a naïve null model that does not take into account tissue structure and cell density variations across the tissue. Hence, these plots should be considered as exploratory analyses rather than formal hypothesis tests.

```
res <- calcMetricPerFov(
    spe=sub, fun="Lest",
    subsetby="sample_id",
    marks="SingleR_label",
    selection="pyramidal CA1",
    rSeq=seq(0, 500, l=100),
    by="sample_id", ncores=1)

plotMetricPerFov(res,
    theo=TRUE, correction="iso",
    x="r", imageId="sample_id")
```

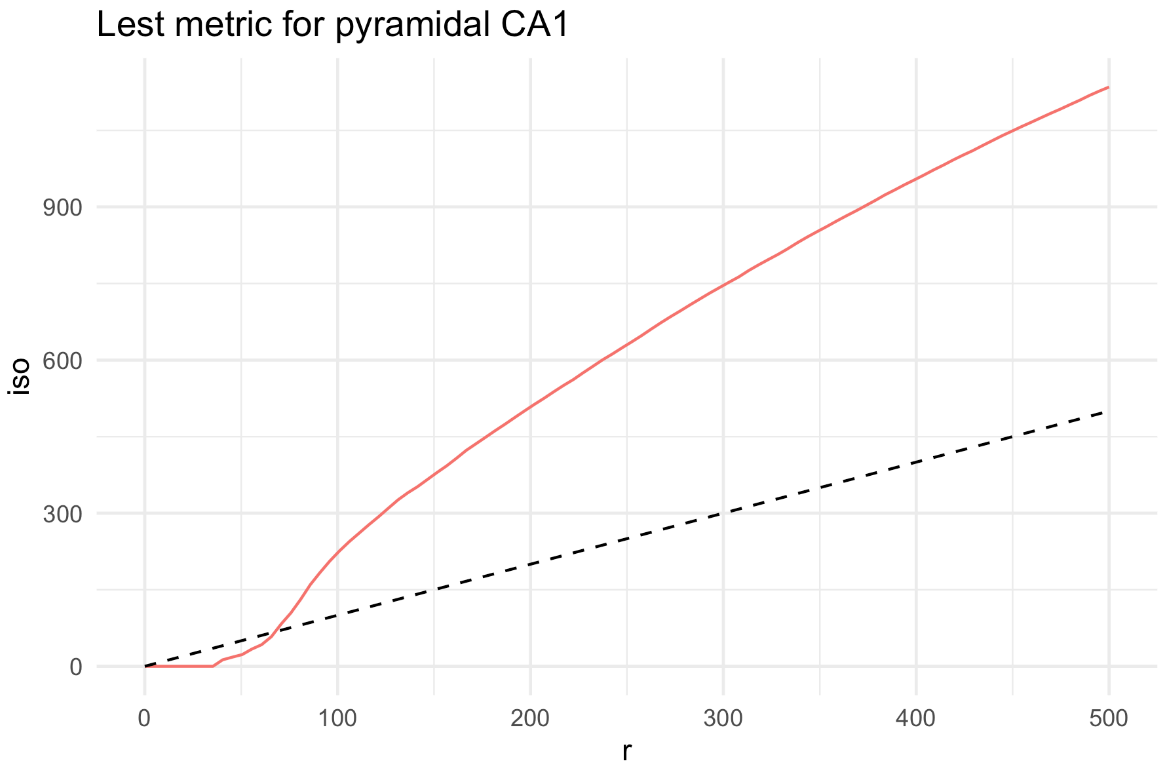

In this type of plots, the dashed line represents the theoretical value of the metric under complete spatial randomness (CSR). Values above this line indicate clustering, while values below indicate dispersion. Here, we can see that pyramidal CA1 neurons tend to cluster together, as expected.

Note that the L-function lays below the CSR line for the first 50 microns, which is a consequence of the cell segmentation that does not

While in the plot above we focused on a single cell type, we can also investigate the interaction between two different cell types, e.g., pyramidal CA1 neurons and oligodendrocytes, using the cross-L function.

```
res <- calcMetricPerFov(
    spe=sub, fun="Lcross",
    subsetby="sample_id",
    marks="SingleR_label",
    selection=c("pyramidal CA1", "oligodendrocytes"),
    rSeq=seq(0, 500, l=100),
    by="sample_id", ncores=1)

plotMetricPerFov(res,
    theo=TRUE, correction="iso",
    x="r", imageId="sample_id")
```

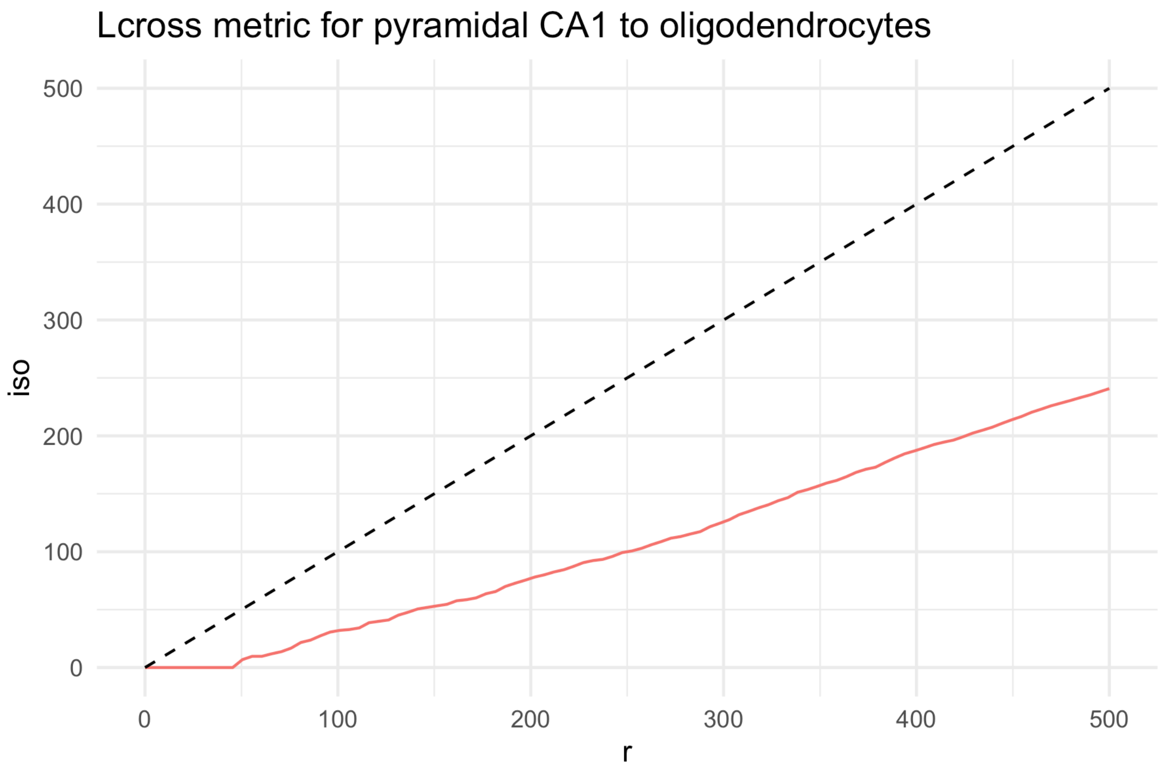

In this case, we can see that pyramidal CA1 neurons and oligodendrocytes tend to be spatially segregated, as indicated by the cross-L function being below the CSR line.

We only briefly discussed cell-cell interactions and spatial exploratory analyses here: we refer interested readers to the comprehensive documentation and tutorials available at the pasta and Voyager websites.

Appendix

References

Atta, Lyla, Kalen Clifton, Manjari Anant, Gohta Aihara, and Jean Fan. 2024. "Gene Count Normalization in Single-Cell Imaging-Based Spatially Resolved Transcriptomics." *Genome Biology* 25 (153). https://doi.org/10.1186/s13059-024-03303-w.

Bhuva, Dharmesh D., Chin Wee Tan, Agus Salim, Claire Marceaux, Marie A. Pickering, Jinjin Chen, Malvika Kharbanda, et al. 2024. "Library Size Confounds Biology in Spatial Transcriptomics Data." *Genome Biology* 25 (99). https://doi.org/10.1186/s13059-024-03241-7.

Cai, Peiying, Mark D Robinson, and Simone Tiberi. 2024. "DESpace: Spatially Variable Gene Detection via Differential Expression Testing of Spatial Clusters." *Bioinformatics* 40 (btae027, 2). https://doi.org/10.1093/bioinformatics/btae027.

Danaher, Patrick, Edward Zhao, Zhi Yang, David Ross, Mark Gregory, Zach Reitz, Tae K Kim, et al. 2022. "Insitutype: Likelihood-Based Cell Typing for Single Cell Spatial Transcriptomics." *BioRxiv*, 2022–10.

de Oliveira, Michelli Faria, Juan Pablo Romero, Meii Chung, Stephen R. Williams, Andrew D. Gottscho, Anushka Gupta, Susan E. Pilipauskas, et al. 2025. "High-Definition Spatial Transcriptomic Profiling of Immune Cell Populations in Colorectal Cancer." *Nature Genetics* 57: 1512–23. https://doi.org/10.1038/s41588-025-02193-3.

Emons, Martin, Samuel Gunz, Helena L. Crowell, Izaskun Mallona, Reinhard Furrer, and Mark D. Robinson. 2025. "Harnessing the Potential of Spatial Statistics for Spatial Omics Data with Pasta." *Nucleic Acids Research* 53 (17): gkaf870. https://doi.org/10.1038/s41467-022-28020-5.

Janesick, Amanda, Robert Shelansky, Andrew D. Gottscho, Florian Wagner, Stephen R. Williams, Morgane Rouault, Ghezal Beliakoff, et al. 2023. "High Resolution Mapping of the Tumor Microenvironment Using Integrated Single-Cell, Spatial and in Situ Analysis." *Nature Communications* 14 (8353). https://doi.org/10.1038/s41467-023-43458-x.

McCarthy, Davis J, Kieran R Campbell, Aaron T L Lun, and Quin F Wills. 2017. "Scater: Pre-Processing, Quality Control, Normalization and Visualization of Single-Cell RNA-Seq Data in r." *Bioinformatics* 33: 1179–86. https://doi.org/10.1093/bioinformatics/btw777.

Moses, Lambda, Pétur Helgi Einarsson, Kayla Jackson, Laura Luebbert, A. Sina Booeshaghi, Sindri Antonsson, Nicolas Bray, Páll Melsted, and Lior Pachter. 2023. "Voyager: Exploratory Single-Cell Genomics Data Analysis with Geospatial Statistics." *bioRxiv*. https://doi.org/10.1101/2023.07.20.549945.

Salim, Agus, Dharmesh D. Bhuva, Carissa Chen, Chin Wee Tan, Pengyi Yang, Melissa J. Davis, and Jean Y. H. Yang. 2025. "SpaNorm: Spatially-Aware Normalization for Spatial Transcriptomics Data." *Genome Biology* 26 (109). https://doi.org/10.1186/s13059-025-03565-y.

Zeisel, Amit, Ana B Muñoz-Manchado, Simone Codeluppi, Peter Lönnerberg, Gioele La Manno, Anna Juréus, Sueli Marques, et al. 2015. "Cell Types in the Mouse Cortex and Hippocampus Revealed by Single-Cell RNA-Seq." *Science* 347 (6226): 1138–42.
